# Supplementary material for: Targeted insertion of partial CFTR cDNA replicates native CFTR expression with restored channel function in CF airway epithelium
Source: Mol Ther Nucleic Acids. 2026 Oct 1;37(4):103123. doi: 10.1016/j.omtn.2026.103123 (PMC13717110; doi:10.1016/j.omtn.2026.103123)
Supplement: Supplementary file 1 [file mmc1.pdf]

OMTN, Volume *37*

Supplemental information

Targeted insertion of partial *CFTR* cDNA replicates native *CFTR* expression with restored channel function in CF airway epithelium

Cristina Barillà, Shingo Suzuki, So Hyun Park, Divyasri Nalluri, Hui Wen, Mohamed A. Hanafy, Daniel Betancourth, Ayushi Umrigar, Shih-Hsing Leir, Katreya Lovrenert, Andrey Sivachenko, Barbara Tabak, Zhong Liu, Rui Zhao, Hillary C. Valley, Andras Rab, Eric J. Sorscher, George M. Solomon, Martin Mense, John P. Carulli, Ann Harris, Gang Bao, and Brian R. Davis

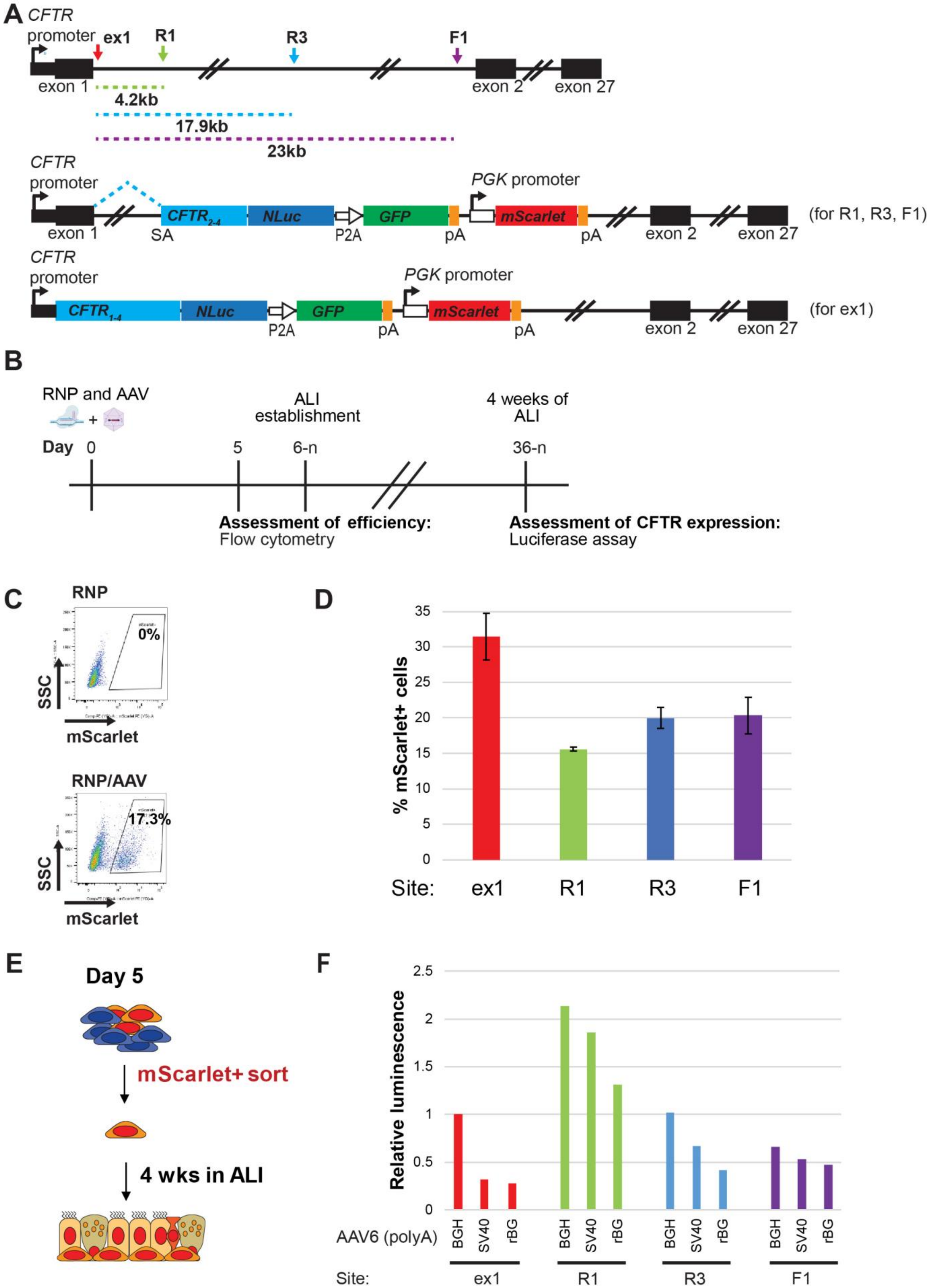
A
CFTR promoter
ex1
R1
R3
F1
exon 1
exon 2
exon 27
4.2kb
17.9kb
23kb
CFTR promoter
PGK promoter
CFTR2-4
NLuc
GFP
mScarlet
SA
P2A
pA
(for R1, R3, F1)
CFTR1-4
(for ex1)
B
RNP and AAV
ALI establishment
4 weeks of ALI
Day 0
5
6-n
36-n
Assessment of efficiency:
Flow cytometry
Assessment of CFTR expression:
Luciferase assay
C
RNP
0%
SSC
mScarlet
RNP/AAV
17.3%
D
% mScarlet+ cells
Site: ex1 R1 R3 F1
E
Day 5
mScarlet+ sort
4 wks in ALI
F
Relative luminescence
AAV6 (polyA)
BGH SV40 rBG
Site: ex1 R1 R3 F1

Figure S1: **Evaluation of *CFTR* intron 1 insertion sites and polyA sequences**. A) Schematics of *CFTR* with focused view on intron 1. The top schematic shows the native structure of intron 1 with vertical arrows marking the position of different gRNAs tested. The middle schematic shows the DNA structure after insertion of the reporter at one of the intron 1 sites. The blue dashed line represents the splicing event required to achieve expression of the *CFTR-NanoLuc* chimera. The bottom schematic shows the DNA structure after insertion of the same reporter but inserted at exon 1. B) Experiment workflow and timeline to assess different reporter transgenes. C) Flow cytometric gating to identify mScarlet+ targeted cells after selection of single and live cells. D) Frequency of mScarlet+ cells at each insertion site measured seven days after transduction. Mean±SD for each insertion site (ex1, R1, R3, F1) reflects the pooled results of three donors -- differing in their polyA signal -- targeted via homology arms to each insertion site. E) Strategy to generate ALI cultures derived from enriched edited cells. F) Summary of luciferase assay, performed at 4 weeks of ALI culture, showing the activity at each insertion site (ex1, R1, R3, F1) for donors DNA differing in their polyA signal culture. The data are reported relative to ex1 site with BGH.

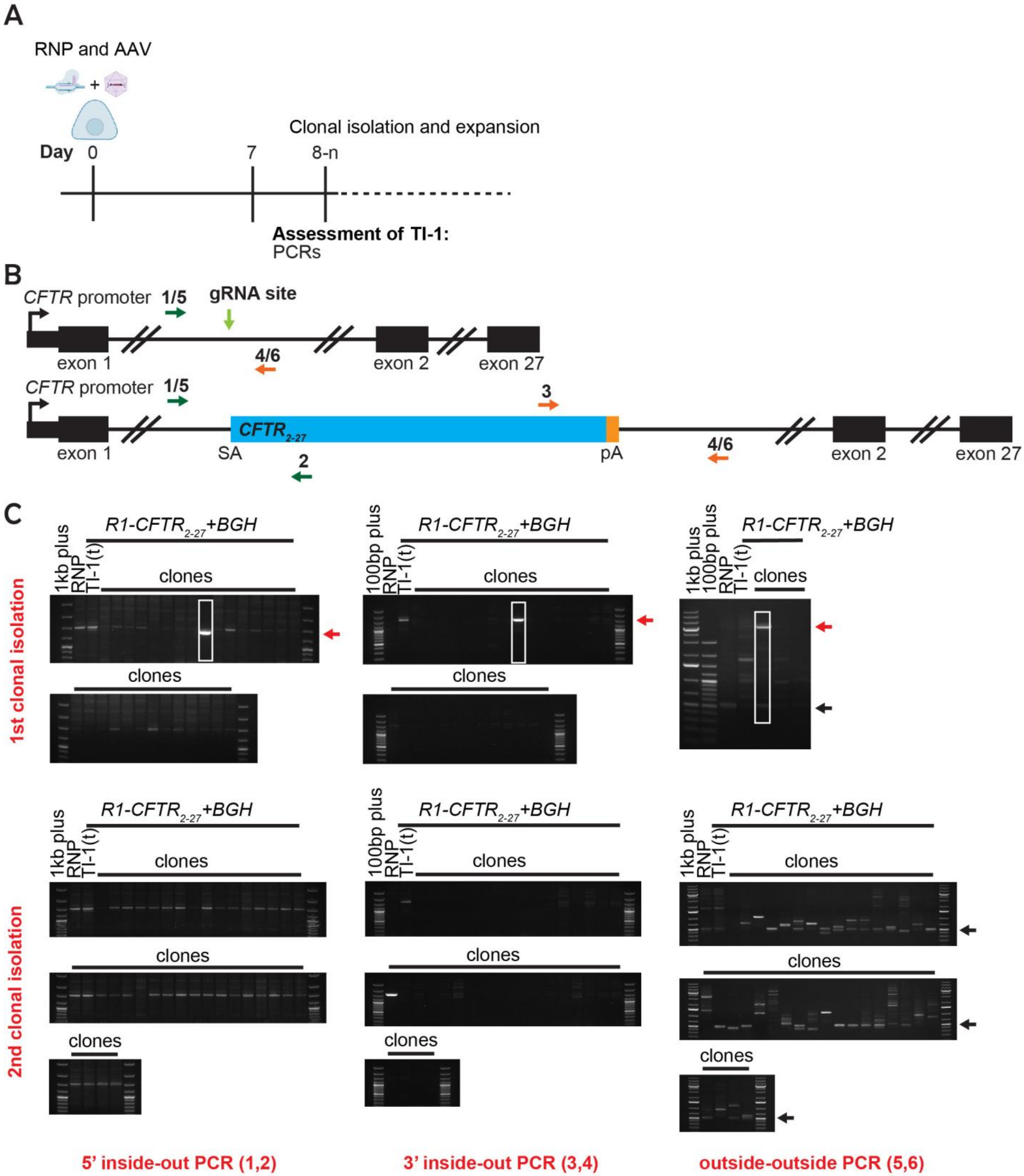

Figure S2: **Inefficient targeted insertion of AAV-6 delivered *CFTR*$_{2\text{-}27}$.** A) Experiment workflow and timeline for AAV-6 delivery for assessing insertion efficiency through AAV-6 delivery of the *CFTR*$_{2\text{-}27}$ transgene. B) Schematic of *CFTR* exon 1, intron 1, and exon 2 highlighting the PCR primers used for the molecular characterization (see Fig 2A). The green arrows represent primers used for the 5' inside-outside PCR; orange arrows represent primers used for the 3' inside-outside PCR; the

green arrow most left and the orange arrow at the most right represent primers used outside-outside PCR. C) Agarose gels showing PCR amplification of either prior or after clonal isolation (from two separate rounds of clonal isolation) of RNP/AAV treated basal cells. A red arrow marks amplicon band consistent with TI-1; a black arrow signifies amplified band from unmodified allele or with small indels. The white rectangle highlights the only positive sample (heterozygous for TI-1) from the three PCR analyses.

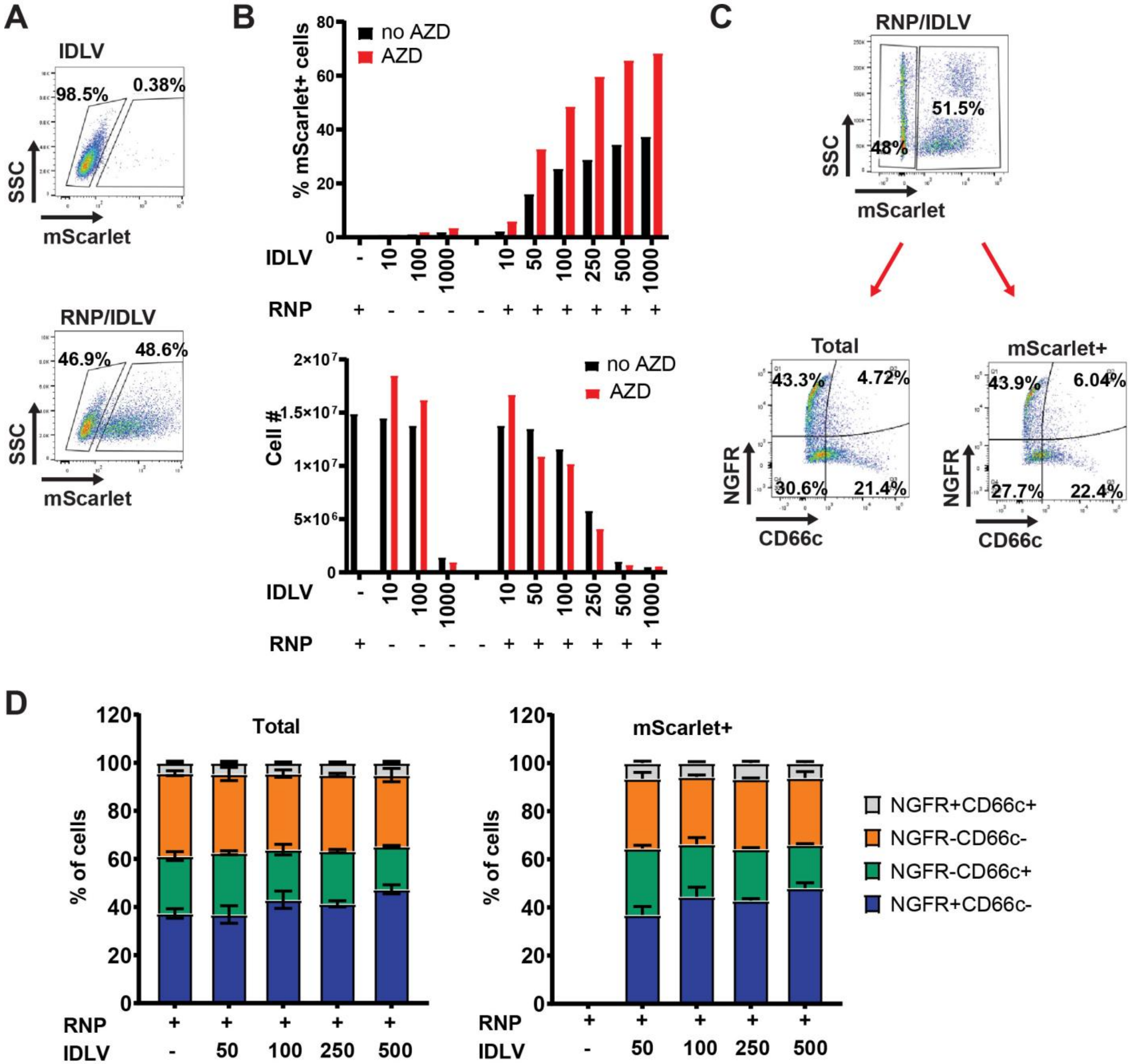

Figure S3: **IDLV delivery facilitates efficient insertion of the reporter construct, in the presence or absence of AZD7648**. A) Flow cytometry gates for identification of mScarlet+ cells after selection of single and live cells. B) Quantification of mScarlet+ cells (%) (top) and cell recovery (#) (bottom) with increasing doses of IDLV nine days post transduction. C) Flow cytometric analysis to examine expression of basal (NGFR+/CD66c-), secretory (NGFR-CD66c+), and other cells (for example, ciliated) (NGFR-CD66c-) markers in either total cells or in the mScarlet+ population. Pre-gating was performed to only show single live cells. D) Quantification of the cell types from C.

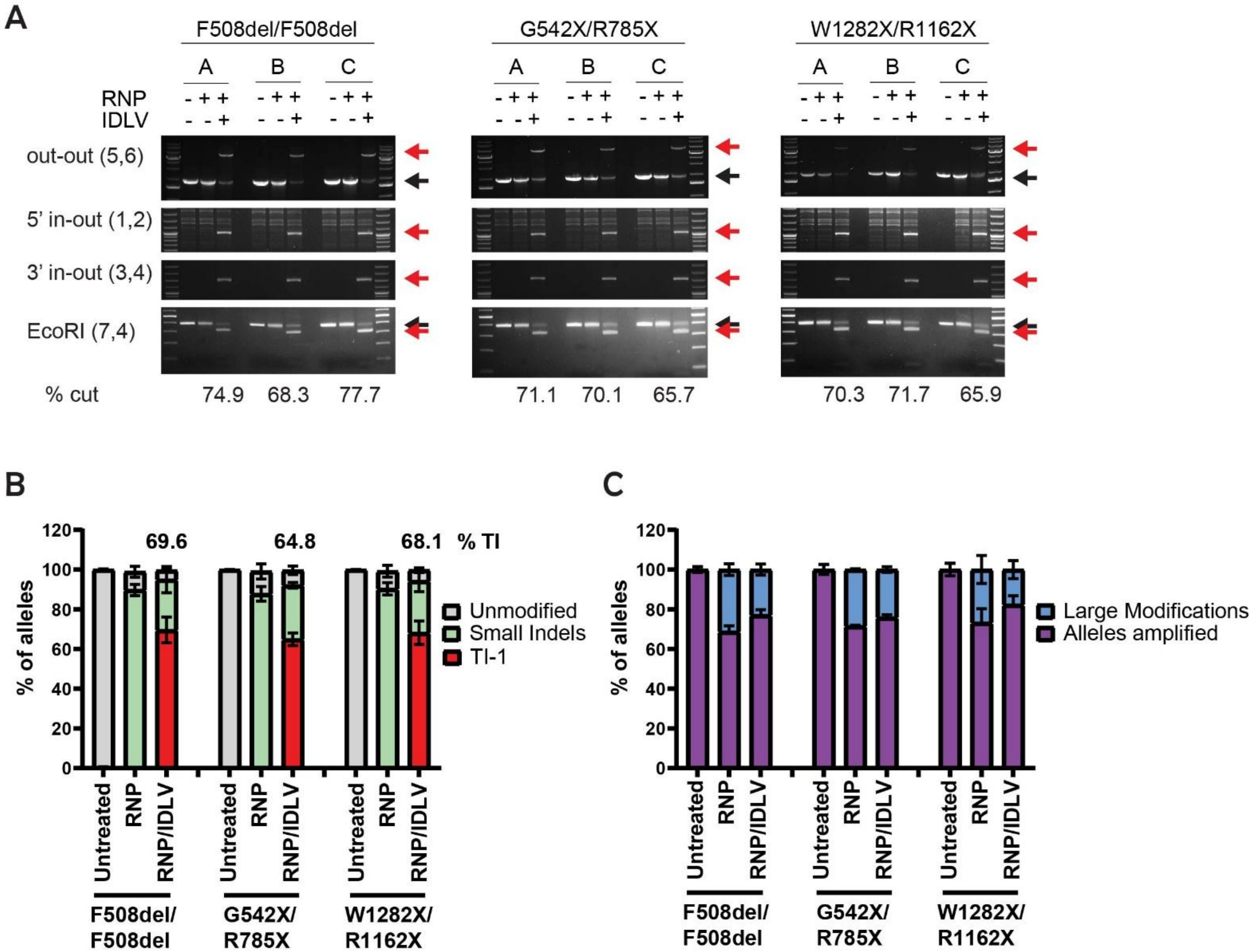

Figure S4: **Evaluation of editing efficiency with IDLV-delivered *CFTR*$_{2\text{-}27}$ transgene**. A) Agarose gels from the three cell donors, showing detection of *CFTR*$_{2\text{-}27}$ insertion in three independent editing experiments (labeled A, B, and C) through PCR using the primers in Fig 2A. Red arrows indicate bands consistent with successful insertion; black arrows indicate bands from unmodified or small indel alleles. B-C) Summary of deep sequencing results (B) and ddPCR copy number assay (C). ddPCR was used to quantify the fraction of PCR-amplifiable alleles measured by NGS. This value was then used to calculate the true proportions of unmodified alleles, small indels, and TI-1 events, as well as the fraction of large genomic modifications that are not amplifiable by PCR, as shown in Fig 2C. Mean±SD of three independent experiments.

A

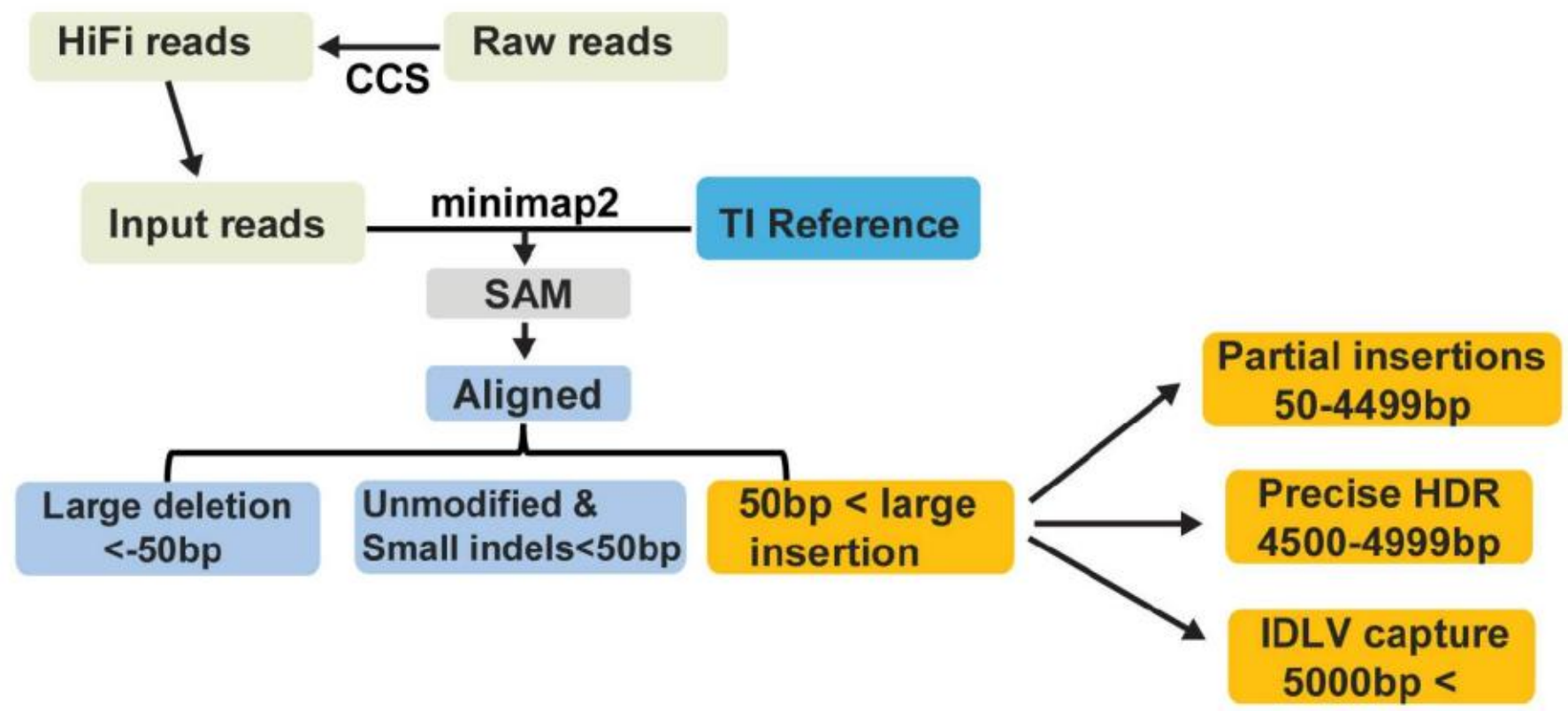

C

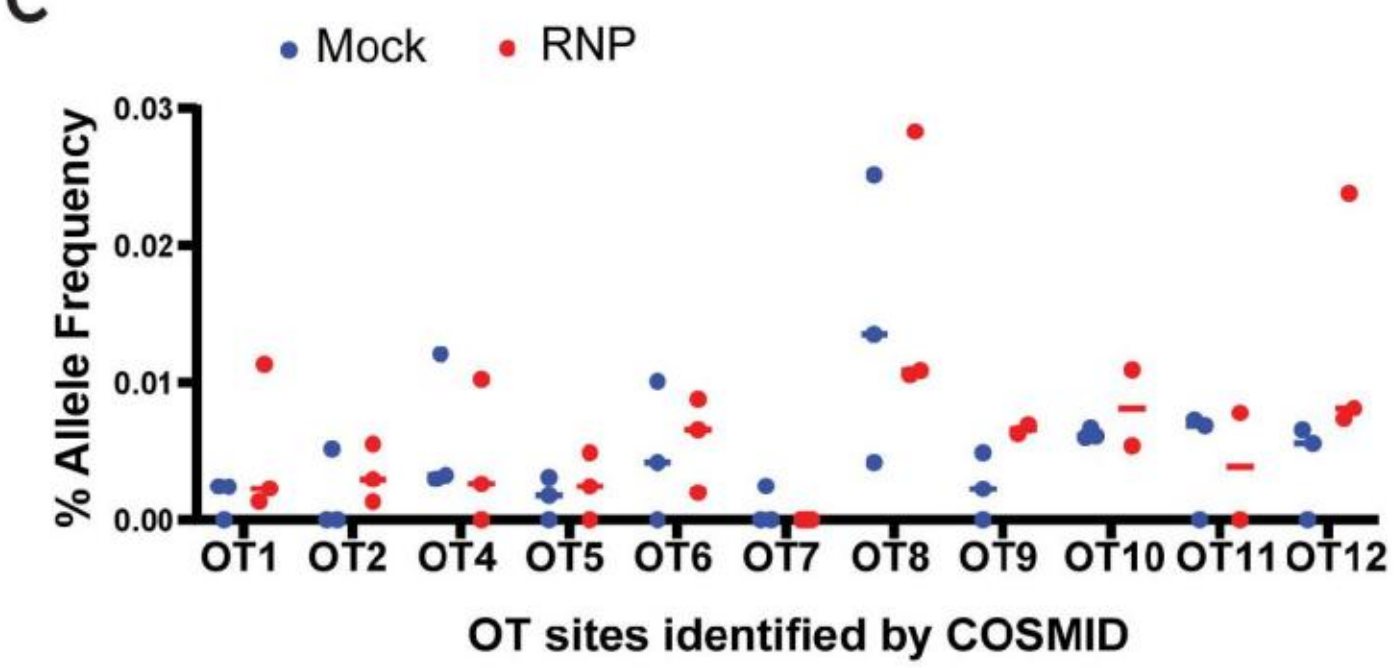

B

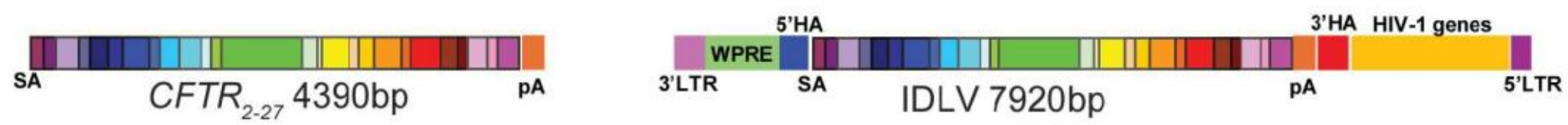

Bin size (bp)	% reads in bin
50-499	3.00
500-999	2.06
1000-1499	2.69
2000-2999	2.69
3000-3999	1.50
4000-4999	64.78
5000-5999	1.28
6000-6999	0.99
7000-7999	0.97
8000-8999	0.29
9000-9999	0.25
10000-10999	0.23
11000-11999	0.10
12000-12999	2.49
13000-13999	0.04
14000-14999	0.01
15000-15999	0.02
20000-20999	0.02

Most common insertion size (bp)	5'	3'	Representative insertion structure
382			
991			$CFTR_{23-27}$
1258			$CFTR_{22-27}$
2321			
3465			
4720 (TI-1)	HDR	HDR	
5889		HDR	
6808	HDR		
7977			
8094	HDR		
9102		HDR	
10061	HDR		
11012	HDR		
12459	HDR		
13626			
14535	HDR		
15015		HDR	
20198	HDR		

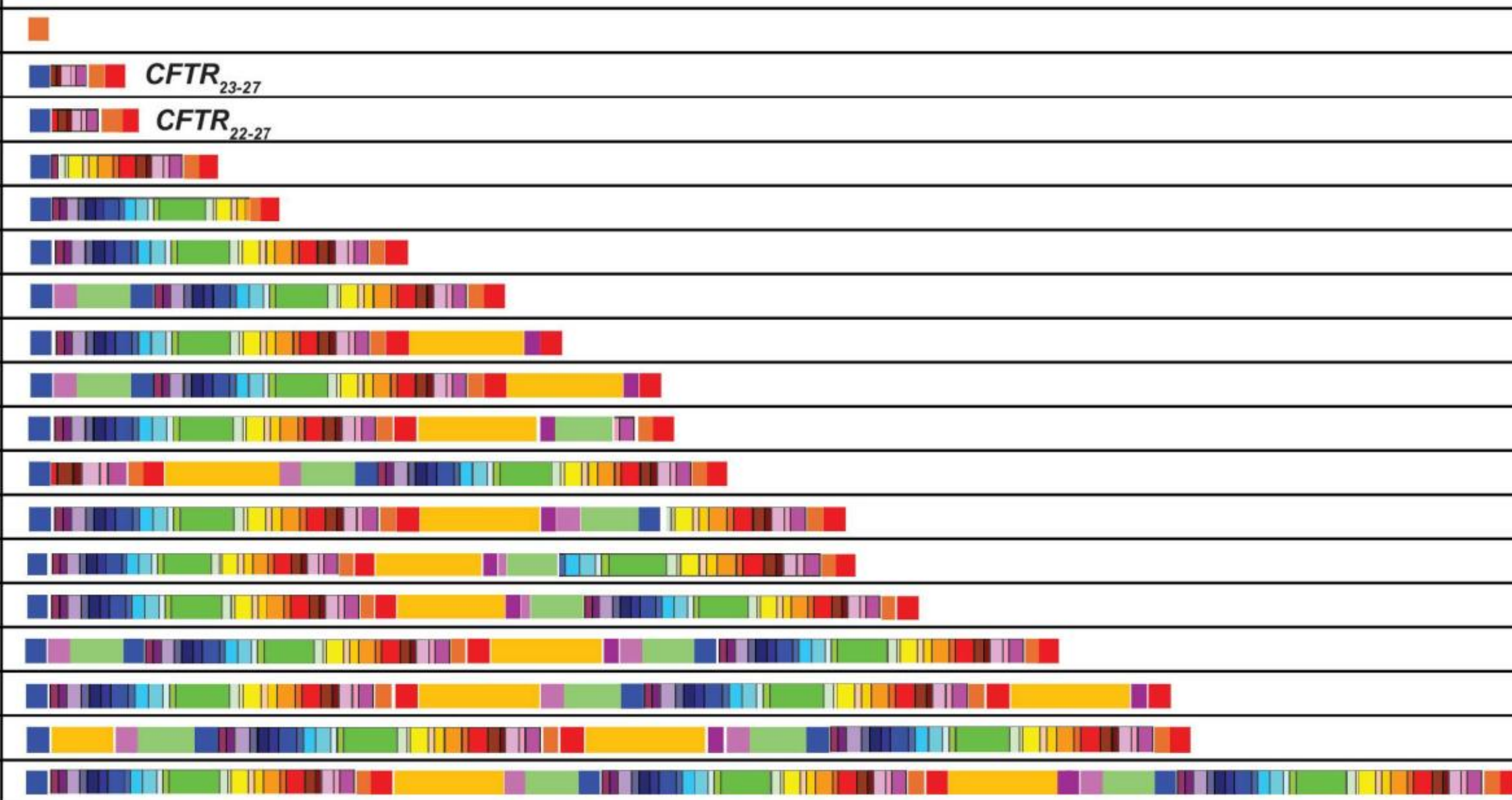

Figure S5: **Analysis of insertion events and undesired outcomes**. A) Variant calling pipeline for SMRT-seq analysis used to identify the modification events in Fig 2D. SMRT-seq generated HiFi reads via Circular Consensus Sequencing (CCS), which were aligned to the TI-1 reference sequence using minimap2. Reads were classified based on insertion and deletion sizes: events ranging from 50 bp deletions to 49 bp insertions were classified as unmodified or indels; deletions > 50 bp were classified as large deletions; and insertions ≥ 50 bp were classified as large insertions. A 4,720 bp insertion corresponds to the TI-1 allele. Large insertions between 50 and 4,499 bp reflect partial IDLV insertions. Reads classified as large insertions were further extracted and analyzed using alignment software to identify diverse IDLV capture patterns, including partial, full-length, and concatemeric IDLV capture. B) Representative insertion structures identified by SMRT-seq. The top schematics show color-coded of *CFTR*$_{2\text{-}27}$ transgene (left; each color represents a different exon) and IDLV donor (right). SMRT-seq reads were grouped by insertion size with a resolution of 1,000 bp bin, and representative structures are shown. The table indicates the size of the most common insertion within a given bin, whether the 5′ and/or 3′ junction retained an HDR structure, and a schematic representation of the most common insertion. Because IDLV capture events were highly heterogeneous, the structures shown represent the most common read within each insertion-size bin rather than all observed configurations. Many IDLV capture events retained a 5′ HDR structure, which, in principle, would allow the allele to express the full-length *CFTR* transgene from the endogenous promoter, even though the correctly targeted cassette is followed by additional IDLV-derived vector sequences. In addition, many IDLV capture events retained the 3′ HDR structure but not the 5′ HDR structure. These alleles would have been classified as TI-1 alleles by the NGS assay because they retain the primer-binding sites and generate the same expected amplicon size as a true TI allele. However, although we detected multiple alleles in this category, their combined frequency was very low. Therefore, these rare outcomes are unlikely to have substantially affected the overall NGS- or ddPCR-based quantification of targeted integration. C) Off-target analysis was performed by deep sequencing to quantify indel frequencies across the top 12 sites identified by COSMID.

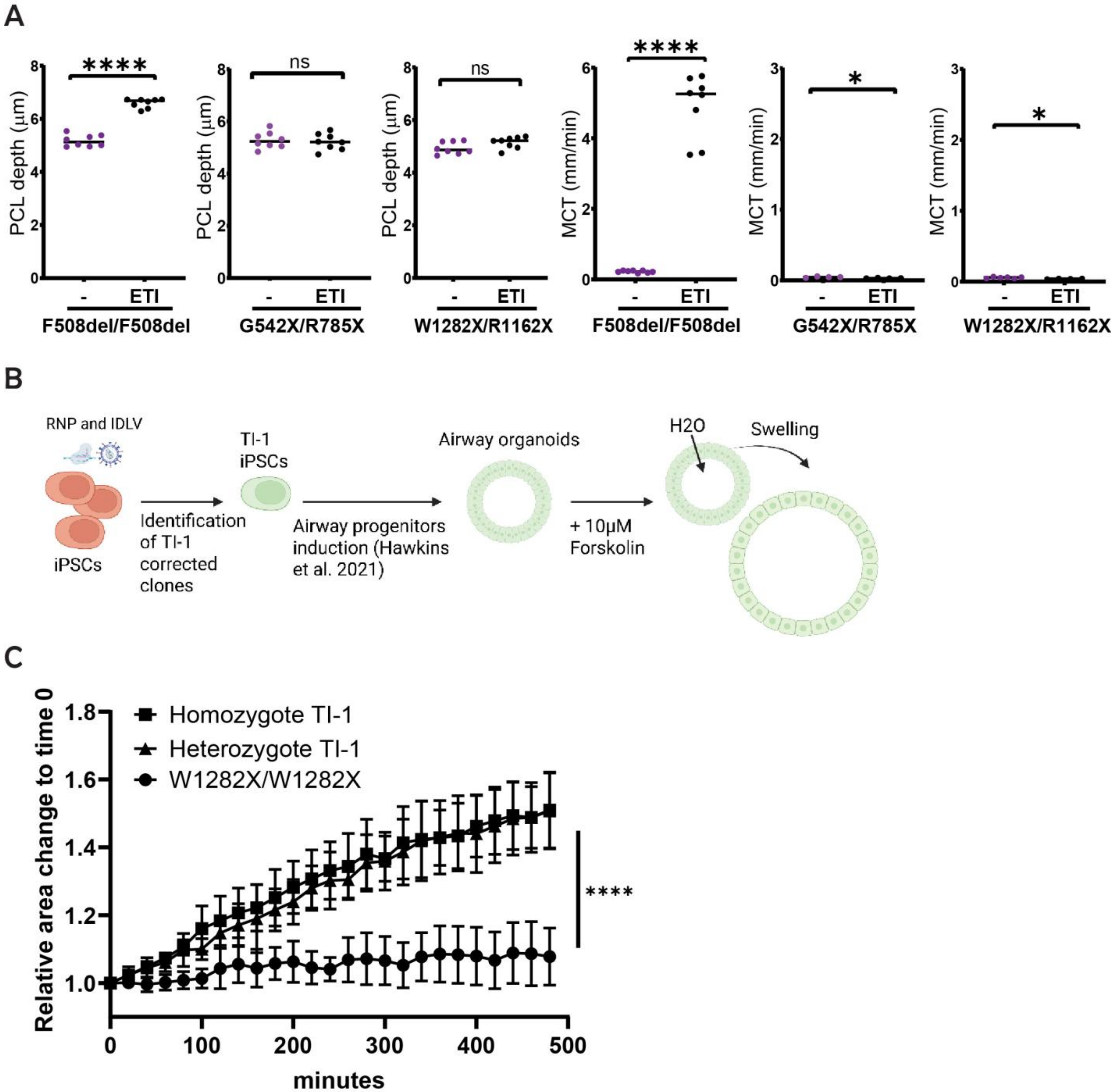

Figure S6: **CFTR functional assays**. A) μOCT summary data for PCL and MCT comparing untreated vs ETI (corresponding to Fig 3D). Data are presented as individual data points from 4 technical replicates (1-2 images each) distributed around the median. B) Schematic for TI-1 and functional evaluation in iPSCs. C) Forskolin induced swelling assay in clonal populations of edited cells vs parental, unedited cells after 30 days of differentiation of iPSCs to airway progenitors. The data are shown relative to time 0. Mean±SD from 5-6 wells.

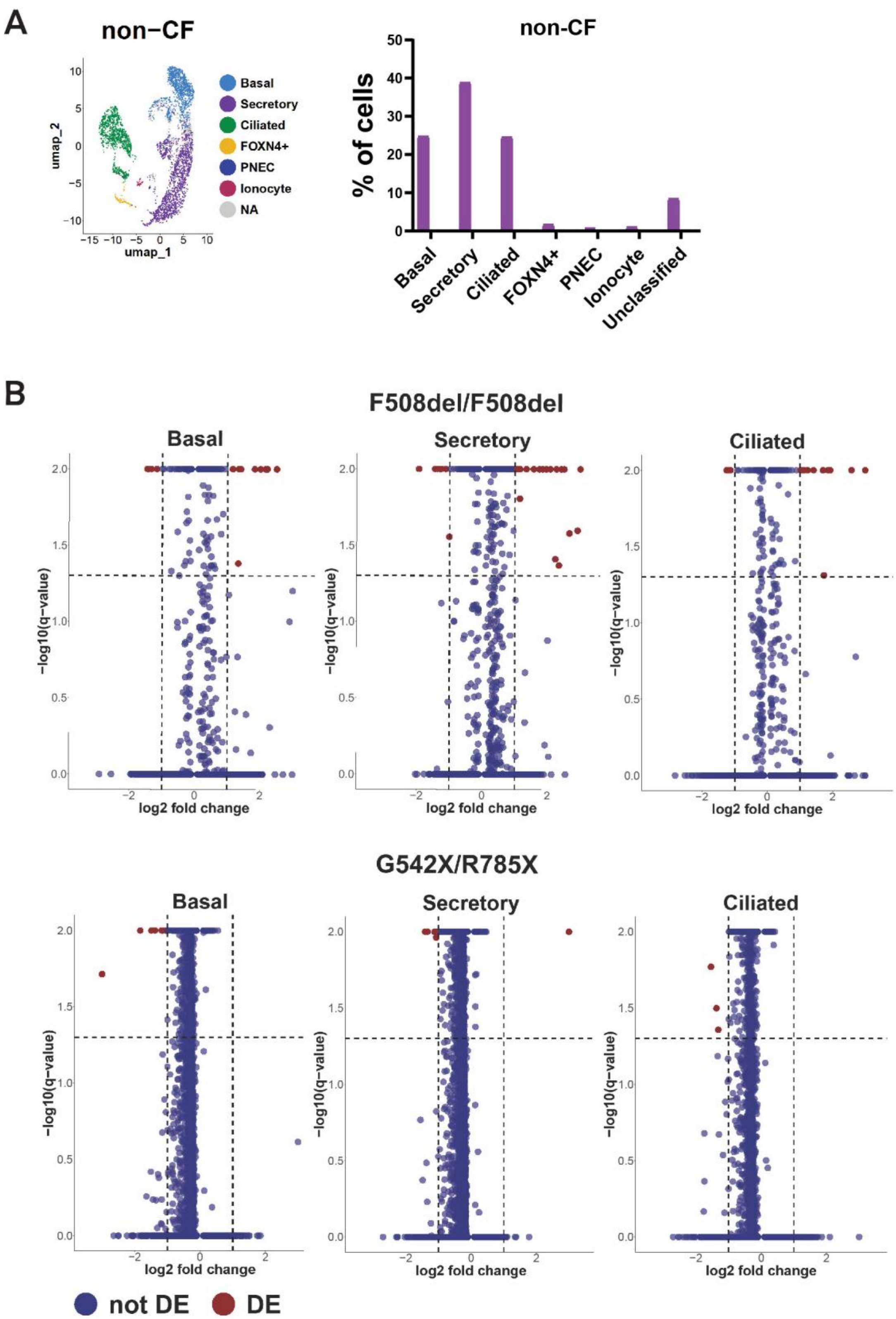

A
non−CF
umap_2
umap_1
Basal
Secretory
Ciliated
FOXN4+
PNEC
Ionocyte
NA
non-CF
% of cells
Basal
Secretory
Ciliated
FOXN4+
PNEC
Ionocyte
Unclassified
B
F508del/F508del
Basal
Secretory
Ciliated
−log10(q−value)
log2 fold change
G542X/R785X
Basal
Secretory
Ciliated
−log10(q−value)
log2 fold change
not DE
DE

Figure S7: **TI-1 treatment in basal cells minimally affects transcriptional profile of derived airway epithelium.** A) Identification and quantification of cell types in non-CF ALI cultures analyzed in parallel to samples in Fig 4B-C. UMAPs (left) and quantification (right) of each cell cluster in non-CF. B) Comparison of gene expression pattern for major cell types in untreated vs bulk edited CF ALI cultures. Volcano plot showing total gene expression in major cell types of ALI cultures comparing untreated and bulk edited primary cells. Genes that are not differentially expressed are in blue, differentially expressed are in red.

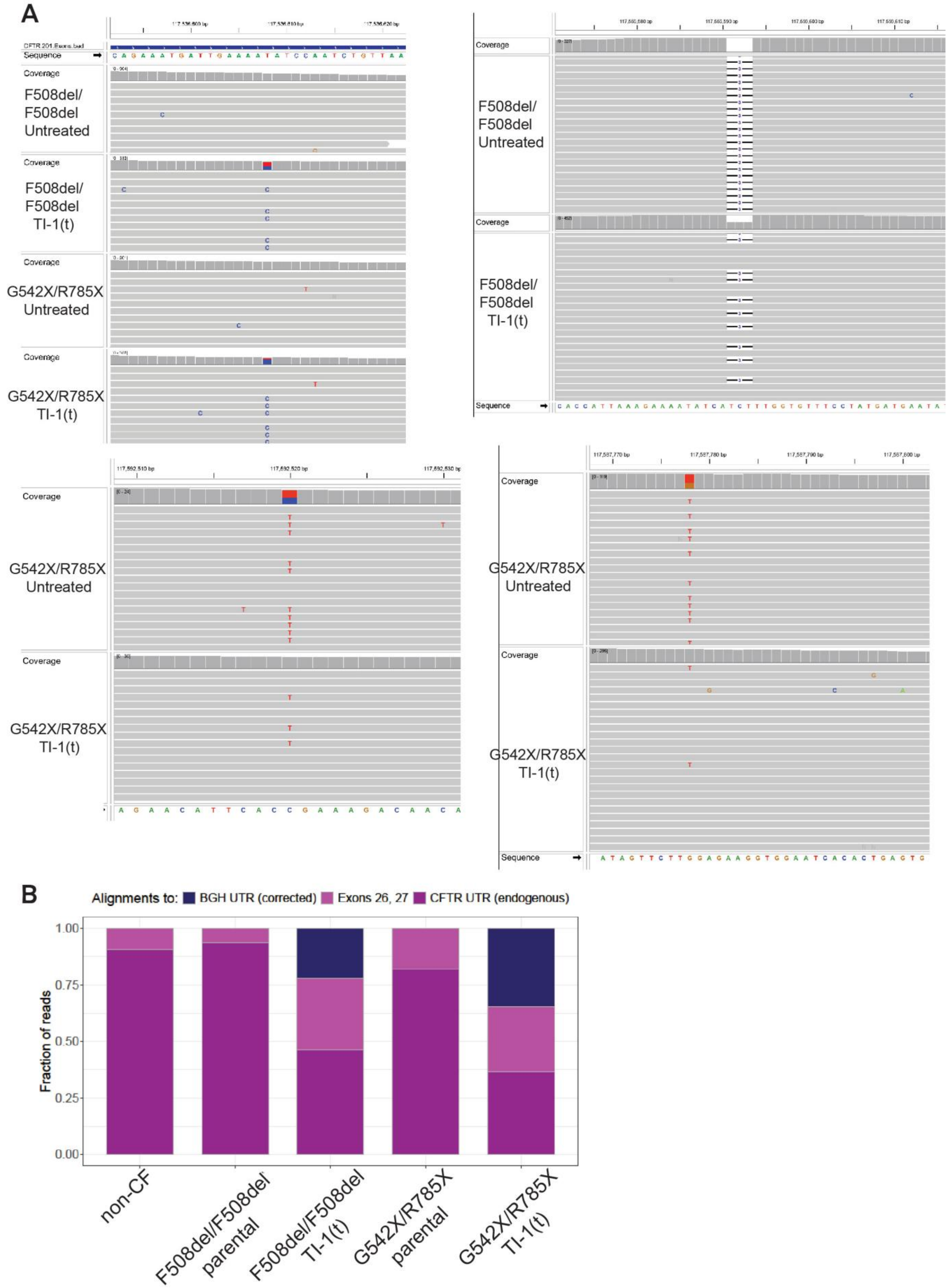

A
F508del/F508del Untreated
F508del/F508del TI-1(t)
G542X/R785X Untreated
G542X/R785X TI-1(t)
Coverage
Sequence
B
Alignments to: BGH UTR (corrected) Exons 26, 27 CFTR UTR (endogenous)
Fraction of reads
0.00
0.25
0.50
0.75
1.00
non-CF
F508del/F508del parental
F508del/F508del TI-1(t)
G542X/R785X parental
G542X/R785X TI-1(t)

Figure S8: A**lignment of sequence reads in regions that distinguish between mutant and corrected *CFTR* mRNAs**. A) Comparison of reads in unedited vs TI-1(t) cells: IGV view of the SNP (T>C) in exon 7 (top left), CTT deletion causing F508del (top right), C>T variant responsible for G542X (bottom left), and G>T variant causing R785X (bottom right). The number and percentage of reads for each are summarized in Fig 7A. B) Fraction of the reads aligning within the native 3'UTR, BGH UTR, and *CFTR* exons 26-27 used for Fig 7B.

Table S1: gRNA sequences used in this study with spCas9.

**gRNA name**	**sequences 5' -> 3'**	**PAM**
CFTRgRNA-ex1	GAAGGTGGCCAACCGAGCTT	CGG
CFTRgRNA-R1	ACTCCATTTAGTCAGTCCAT	GGG
CFTRgRNA-R3	TCCAGGATTTACAACCATAC	AGG
CFTRgRNA-F1	AAAGTTATGATCCTACCCTC	AGG

Table S2: Sequence of primers used in this study.

Table S3: List of antibodies used for immunostaining.

**Antibody**	**Cat#**	**Company**
Mouse monoclonal anti-Acetylated Tubulin (ACT) (6-11B-1)	T7451	Millipore-Sigma
Uteroglobin/SCGB1A1 Antibody (394324)	MAB4218-SP	Novus Biologicals
Rabbit monoclonal anti-MUC5AC (E309I)	61193:	Cell Signaling Technologies
Mouse monoclonal anti-MUC5B (A-3)	Sc-393952	Santa Cruz
Rabbit monoclonal anti-p75NTR/NGFR (D4B3)	8238	Cell Signaling Technologies

Mouse monoclonal anti-TP63 (4A4)	CM163A	Biocare
Rabbit monoclonal anti-KRT5 (D4U8Q)	25807	Cell Signaling Technologies
Donkey anti-mouse IgG Alexa Fluor Plus 647 Highly cross-absorbed	A32787	Thermo Fisher Scientific
Donkey anti-rabbit IgG Alexa Fluor Plus 488 Highly cross-absorbed	A32790	Thermo Fisher Scientific
Donkey anti-rat IgG Alexa Fluor Plus 555 Highly cross-absorbed	A48270	Thermo Fisher Scientific

Video S1. **F508del/F508del TI-1(t) mucus movement**. Video generated by live imaging of a representative ALI culture at 4 weeks of differentiation. Images were captured at 40 frames/second and 512 lines/frame using uOCT and used for quantifications showed in Fig. 3D.

Video S2. **W1282X/R1162X TI-1(t) mucus movement**. Video generated by live imaging of a representative ALI culture at 4 weeks of differentiation. Images were captured at 40 frames/second and 512 lines/frame using uOCT and used for quantifications showed in Fig. 3D.
