# Supplementary material for: Targeted insertion of partial CFTR cDNA replicates native CFTR expression with restored channel function in CF airway epithelium
Source: Mol Ther Nucleic Acids. 2026 Oct 1;37(4):103123. doi: 10.1016/j.omtn.2026.103123 (PMC13717110; doi:10.1016/j.omtn.2026.103123)
Supplement: Supplementary file 2 [file mmc5.pdf]

Molecular Therapy
Nucleic Acids
Original Article

Targeted insertion of partial *CFTR* cDNA replicates native *CFTR* expression with restored channel function in CF airway epithelium

Cristina Barillà,[1,2,9] Shingo Suzuki,[1,2,3,9] So Hyun Park,[4] Divyasri Nalluri,[1] Hui Wen,[3] Mohamed A. Hanafy,[1,3] Daniel Betancourth,[4] Ayushi Umrigar,[5] Shih-Hsing Leir,[5] Katreya Lovrenert,[5] Andrey Sivachenko,[6] Barbara Tabak,[6] Zhong Liu,[3,7] Rui Zhao,[3,7] Hillary C. Valley,[6] Andras Rab,[8] Eric J. Sorscher,[8] George M. Solomon,[1,3] Martin Mense,[6] John P. Carulli,[6] Ann Harris,[5] Gang Bao,[4] and Brian R. Davis[1,2,3]

[1]Department of Medicine, University of Alabama at Birmingham, Birmingham, AL 35294, USA; [2]Center for Stem Cells and Regenerative Medicine, University of Texas Health Science Center at Houston, Houston, TX 77030, USA; [3]Gregory Fleming James Cystic Fibrosis Research Center, University of Alabama at Birmingham, Birmingham, AL 35294, USA; [4]Department of Bioengineering, Rice University, Houston, TX 77030, USA; [5]Department of Genetics and Genome Sciences, Case Western Reserve University, Cleveland, OH 44106, USA; [6]Cystic Fibrosis Foundation Therapeutics Lab, Cystic Fibrosis Foundation, Lexington, MA 02421, USA; [7]Department of Biochemistry and Molecular Genetics, University of Alabama at Birmingham, Birmingham, AL 35294, USA; [8]Department of Pediatrics, School of Medicine, Emory University, Atlanta, GA 30322, USA

Our objective is to develop a universal gene editing therapeutic for individuals with cystic fibrosis (CF). Due to the large number of *CFTR* variants, we sought to develop a correction methodology utilizing targeted integration of *CFTR*$_{2\text{-}27}$ sequence into *CFTR* intron 1 (TI-1). Following electroporation of CF primary airway basal stem cells with intron 1-targeted Cas9/gRNA, we delivered *CFTR*$_{2\text{-}27}$ donor, flanked by a splice acceptor and polyadenylation signal, via an integrase defective lentiviral vector. Molecular characterization revealed highly efficient integration of the *CFTR*$_{2\text{-}27}$ sequence into the native *CFTR* intron 1 with most insertion events reflecting the desired homology-directed repair. Fully differentiated airway epithelium, derived from TI-1 treated basal cells without enrichment for corrected cells, exhibited functional restoration of CFTR channel activity with significant improvement of mucus hydration and transport. ATAC-seq revealed that the native *CFTR* chromatin architecture was unaffected by the *CFTR*$_{2\text{-}27}$ sequence insertion, and importantly, scRNA-seq demonstrated that the cell-type-specific expression of the introduced transgene replicated that of the endogenous *CFTR*. In this report, we demonstrate an almost completely mutation-agnostic therapeutic approach that is capable of functional CFTR restoration. These results may also be relevant to *CFTR*$_{2\text{-}27}$ integration via other editing and delivery methodologies.

INTRODUCTION

In the last decade, significant advances have been made in the treatment of cystic fibrosis (CF), an autosomal recessive disease caused by mutations in the anion channel-encoding *CFTR* gene.[1,2] Largely responsible for this transformation has been the development and approval of small molecule drugs—modulators—that either alone or in combination (e.g., elexacaftor/tezacaftor/ivacaftor [ETI], Trikafta; or vanzacaftor/tezacaftor/deutivacaftor, Aleftryk) are able to rescue the function of CFTR protein in most people with CF (PwCF). However, this therapy is effective only when some level of mutant CFTR protein is produced such that the modulators can act upon it to improve protein folding and thus trafficking to the plasma membrane, and/or to potentiate its channel function. The *CFTR* variant landscape is highly complex, with greater than 1,000 disease causing mutations distributed across the entire gene and additional variants still under investigation.[3] Numerous *CFTR* variants encode premature termination codons (PTCs) or result in severe splicing defects that prevent protein production. Most PwCF with two such variants cannot presently benefit from modulator therapy and are the current focus for development of new treatments and potential cures including delivery of the entire wild-type (WT) *CFTR* either as mRNA or cDNA,[4] or mutation-specific gene editing approaches such as base editors and prime editors.[5,6]

We have primarily focused on the development of universal gene correction approaches that insert a corrective partial *CFTR* cDNA (hereafter referred to as corrective transgene) into the native mutant

Received 21 April 2026; accepted 30 September 2026;
https://doi.org/10.1016/j.omtn.2026.103123.

[9]These authors contributed equally

Correspondence: Shingo Suzuki, Department of Medicine, University of Alabama at Birmingham, Birmingham, AL 35294, USA.
E-mail: shingosuzuki@uabmc.edu
Correspondence: Brian R. Davis, Department of Medicine, University of Alabama at Birmingham, Birmingham, AL 35294, USA.
E-mail: bdavis34@uab.edu

Published by Elsevier Inc. on behalf of The American Society of Gene and Cell Therapy.
This is an open access article under the CC BY license (http://creativecommons.org/licenses/by/4.0/).

CFTR locus, capable, in principle, of correcting any variant downstream of the insertion site. Since CF is an autosomal recessive disease, correction of even one mutant *CFTR* allele will restore the carrier state. In a prior work,[7] we reported efficient targeted integration of the corrective transgene *CFTR* exons 9 through 27 ($CFTR_{9\text{-}27}$) into *CFTR* intron 8 (TI-8) of primary CF patient airway basal stem cells; $CFTR_{9\text{-}27}$ was preceded by a splice acceptor (SA) sequence and followed by a polyadenylation (polyA) signal. In that study, we utilized the homology-directed repair (HDR) pathway for TI, employing sequence-specific zinc finger nucleases to induce a double-strand break (DSB) at the target site in intron 8, and co-delivered via AAV-6 the donor DNA, flanked by homology arms (HAs) to serve as template. Molecular analysis of cells demonstrated that 50%–62% of alleles were successfully targeted.[7] When basal stem cells were treated with the TI-8 methodology and differentiated, without selection for corrected cells, into airway epithelium under air-liquid interface (ALI) culture conditions, successful correction was demonstrated at the level of *CFTR* mRNA transcription, restoration of mature, glycosylated CFTR protein expression, and rescue of CFTR channel function at levels of 34%–56% of non-CF based on Ussing assay.[7] In a later study, we demonstrated that such modified basal stem cells are also able to differentiate into airway epithelium in an *in vivo* xenograft model.[8]

Although the aforementioned methodology would potentially be applicable for PwCF having at least one of their causative *CFTR* mutations downstream of *CFTR* intron 8, our recent focus has been developing an approach potentially beneficial to all, or nearly all, PwCF. Targeted integration of *CFTR* exons 2 through 27 ($CFTR_{2\text{-}27}$) in intron 1 (TI-1) offers this potential. Nonetheless, moving upstream of intron 8 to intron 1 introduces new challenges: (1) the increased size of $CFTR_{2\text{-}27}$ vs. $CFTR_{9\text{-}27}$ required that we identify a vector system still capable of delivering, in a single payload, the corrective transgene; (2) due to the significant expanse (∼24 kb) of *CFTR* intron 1 as well as the presence of regulatory sequences in intron 1 identified by DNase-hypersensitivity mapping,[9,10] it was important for us to determine whether there are significant constraints on where targeted insertion could give rise to expression of the corrective transgene.

In developing an editing strategy for the CF airway epithelium, our focus has primarily been on achieving efficient *CFTR* correction in airway basal cells. Since these cells serve as resident stem cells of the airway, correction would be predicted to be long lived in the self-renewing basal cell compartment and maintained in the tissue as corrected basal stem cells give rise to their progeny (e.g., secretory, ciliated, and ionocyte cells).[8] Our rationale for targeting correction to the endogenous *CFTR* locus, thereby exploiting the native promoter, regulatory elements, and chromatin architecture, was to achieve physiologically appropriate levels of the corrected *CFTR* mRNA in a cell-type-specific manner that replicates native *CFTR* gene expression. Since native *CFTR* is expressed at the highest level in ionocytes, at a moderate level in a significant fraction of secretory cells, and typically at low to negligible level in basal and ciliated cells,[11–13] we were particularly interested in this study to assess whether the same pattern of expression is demonstrated by corrected *CFTR*.

RESULTS

Selection of intron 1 insertion sites

With the goal of targeting integration of a corrective $CFTR_{2\text{-}27}$ transgene into the endogenous *CFTR* intron 1 (TI-1), we first sought to identify candidate sites for transgene insertion and expression. Since sequence-specific targeted insertion through HDR involves induction of a sequence-specific double-strand DNA (dsDNA) break via Cas9/gRNA ribonucleoprotein (RNP) complex as well as homology sequences that flank the donor sequences to target the insertion, we selected intron 1 sites that lacked repeat sequences and were without common single nucleotide polymorphisms (SNPs) which could interfere either with gRNA recognition or HA specificity. We also avoided previously identified regulatory sequences present within intron 1 such as 185 + 10 kb[10] and sites of CCCTC-binding factor (CTCF) occupancy[9] which, if disrupted, could potentially alter regulation of *CFTR* expression. From this initial screening (see "materials and methods"), we identified three sites matching our criteria (Figure S1A; Table S1) and developed a reporter system to enable evaluation of these sites for integration efficiency and level of expression. To quantify insertion efficiency, we included a constitutive expression cassette (*PGK-mScarlet-SV40)* in the donor construct; after performing TI in primary CF basal cells, a flow cytometric assessment of % mScarlet$^+$ cells would give an approximate measure of whether a given site was, in principle, capable of being targeted, and subsequent sorting for mScarlet$^+$ cells would yield an enriched population of TI-positive cells for analysis of expression. The reporter also included *CFTR* exons 2–4 linked to NanoLuc luciferase (*NLuc*); this element was preceded by an SA to facilitate splicing between the native *CFTR* exon 1 and the donor *CFTR* exon 2, and followed by a polyA signal (Figure S1A). Thus, the construct was designed to generate an in-frame chimeric protein comprising the coding regions of CFTR exons 1–4 fused to NLuc (Figure S1A). In addition, we designed and integrated a similar reporter but directly connected to exon 1 as a splicing-independent control (Figure S1A; Table S1). The linkage of a GFP reporter to $CFTR_{2\text{-}4}$-Nluc via the P2A sequence (as shown in Figure S1A) was designed to also readout expression in fully differentiated airway epithelium generated through ALI cultures. However, perhaps due to the very low CFTR expression level in airway epithelium and/or insufficient sensitivity of our flow cytometric analysis, the number of cells with clearly distinguishable GFP expression was very small. Consequently, we utilized luminescence as functional readout of expression. We also investigated whether expression of the reporter transgene would be affected by the choice of polyA signal. To this end, we constructed donors targeting the four selected insertion sites, containing either of three different commonly used polyA signal sequences (bovine growth hormone [BGH], Simian virus 40 [SV40], or rabbit beta globin [rBG]). Our primary selection criterion, provided that the full donor could be efficiently packaged and delivered, was maximization of the luciferase expression level.

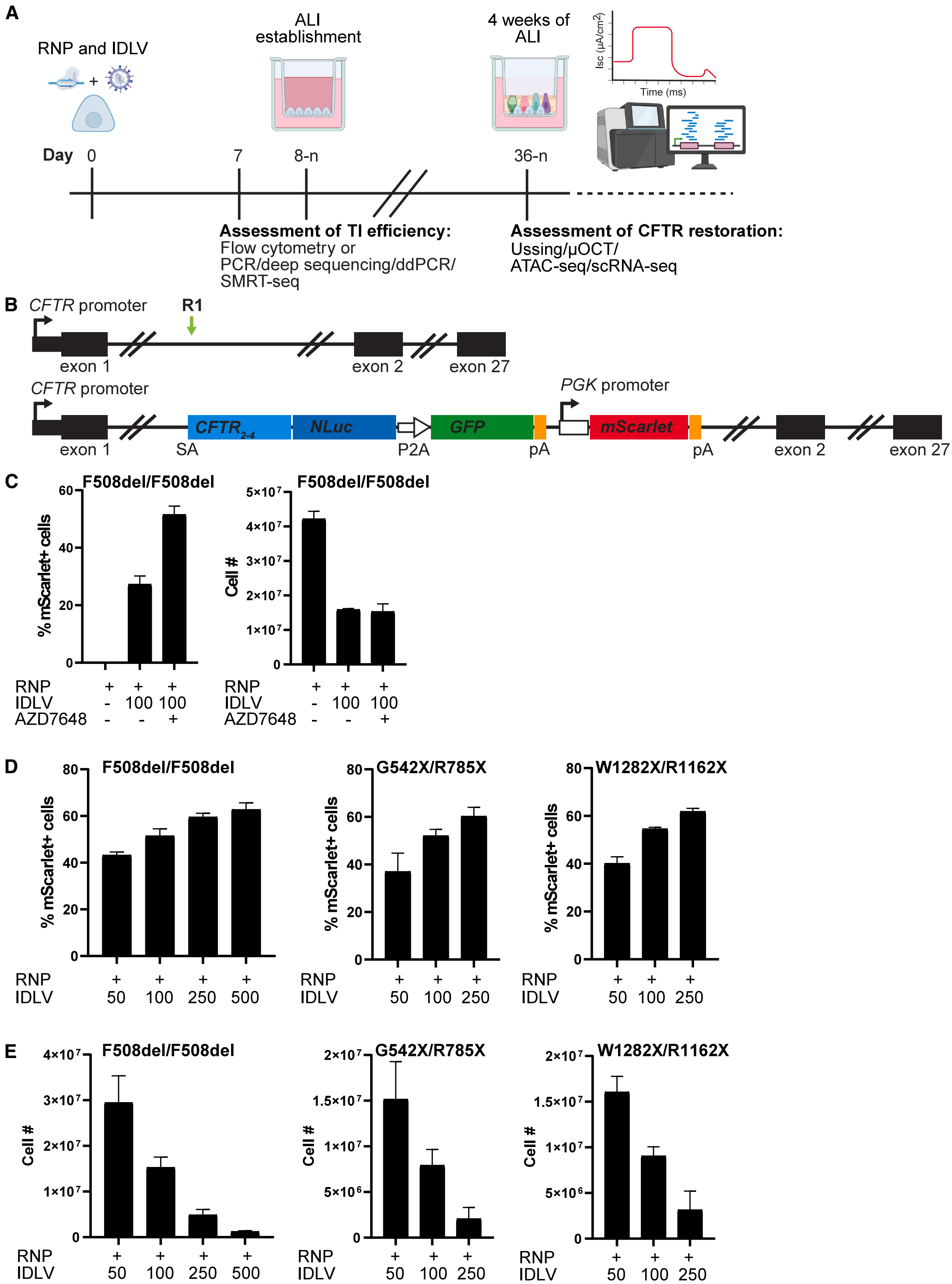

(legend on next page)

Evaluation of intron 1 insertion sites

To test the selected sites for insertion and expression of the aforementioned reporters, RNPs were delivered to primary F508del/F508del airway basal cells via electroporation (Figure S1B). Immediately afterward, we performed AAV-6 mediated delivery of the reporter flanked by HAs (∼250 bp for each HA) specific for each site. After 1 week, we observed ∼15%–30% mScarlet$^+$ cells across all four sites (Figures S1B and S1D); delivery of AAV-6 donor alone yielded no mScarlet$^+$ cells at this time point. mScarlet$^+$ basal cells were sorted from each sample to establish enriched populations of targeted cells; these cells were then differentiated into ALI cultures to yield fully developed airway epithelium (Figures S1B and S1E). Luminescence analysis at 4 weeks of ALI culture demonstrated that integration at all four sites (ex1 [control], R1, R3, and F1) gave rise to luciferase expression. Interestingly, two (R1, R3) of three (R1, R3, F1) intron 1 sites had comparable, or higher, levels of expression than the control (ex1) (Figure S1F). Furthermore, the level of luciferase expression at each site was further modulated by the choice of the polyA signal, with BGH supporting higher expression than SV40 and rBG (Figure S1F). Integration of the reporter at one particular site (R1) gave rise to higher luciferase expression with all three tested polyA signals (Figure S1F). Our results indicate there is some flexibility across intron 1 for selection of integration sites that are compatible with expression of the chimeric *$CFTR_{2\text{-}4}$-luciferase* reporter. Since the R1 site and the BGH polyA signal yielded the highest level of reporter expression, we chose to utilize these elements for integration of the *$CFTR_{2\text{-}27}$* corrective transgene.

AAV-6 delivery of *$CFTR_{2\text{-}27}$* corrective transgene resulted in inefficient TI

In our prior work, we utilized AAV-6 for delivery of *$CFTR_{9\text{-}27}$* (∼4.3 kb including ∼300 bp for each HA). Although the AAV packaging capability is ∼4.7 kb (4.4 kb donor plus inverted terminal repeats),[14,15] there have been reports of successful AAV delivery of up to 5.2 kb.[14,15] Consequently, we first attempted AAV-6 mediated delivery of *$CFTR_{2\text{-}27}$* (Figures S2A and S2B; ∼5 kb, with ∼150 bp for each HA) but did not observe *$CFTR_{2\text{-}27}$* insertion in treated cells (Figure S2C). Subsequent clonal isolation revealed that the insertion efficiency per allele was less than 1% (Figure S2C). Based on these observations, we sought other delivery options.

IDLV donor delivery mediates efficient TI in airway basal stem cells

Integrase-defective lentiviral (IDLV) vectors have previously been shown capable of delivering donor templates to hematopoietic stem progenitor cells (HSPCs) and induced pluripotent stem cells (iPSCs) for HDR-mediated TI.[16,17] In addition to allowing packaging of larger donors (∼10 kb of total DNA with 8–9 kb of donor),[18] they have also been shown in HSPCs to elicit a lower cellular and genomic toxicity in comparison with AAV delivery.[17] To test whether IDLV donor delivery can yield efficient TI in basal cells, immediately following electroporation of RNPs targeting the R1 site, we delivered the same chimeric reporter via IDLV (Figures 1A and 1B; BGH polyA sequence).

At 9 days post-treatment, mScarlet$^+$ cells were present at a frequency of less than 0.5% with donor only and 27.3% with RNP plus donor (Figure 1C; Figure S3B). Although this is already a significant TI frequency, we examined whether it was possible to further increase HDR efficiency by including an inhibitor of non-homologous end joining (NHEJ). In recent work, the DNA-dependent protein kinase (DNA-PK) inhibitor AZD7648 (AZD) increased HDR-mediated TI with AAV donors in a variety of cell types including airway basal cells.[19] When we included the AZD compound in TI experiments with the IDLV donor, the frequency of mScarlet$^+$ cells was increased to 51.5% (Figure 1C).

To maximize the yield of successfully edited cells, the dose of viral vector requires optimization: although higher vector dose generally increases the level of HDR-mediated TI, increased vector dose can result in decreased total yield of modified cells. This decrease can either result from a transient reduction in cell proliferation followed by recovery, or can be due to cytotoxicity. When a range of IDLV doses was examined, we observed the expected dependence of TI-1 frequency on IDLV dose (Figure S3B). Furthermore, for every IDLV dose (measured in titer units [TU]/cell) evaluated, AZD improved TI efficiency approximately 2-fold. A transient IDLV-mediated decrease in cell number was observed at 50–100 TU/cell; however, there was little evidence of cell death at these doses and the cell number recovered. In contrast, virus-induced cell toxicity was significant at 250 TU/cell and even more extreme at 500–1,000 TU/cell, irrespective of AZD presence or absence (Figure S3B). We performed the IDLV-based TI-1 protocol in primary basal cells derived from different PwCF to assess the variation in TI-1 efficiency across donors. Cells from all three donors, in the presence of 0.5 μM AZD, showed similar trends in TI-1 efficiency and cell recovery from the TI procedure (Figures 1D and 1E). Furthermore, when ALI cultures derived from cells subjected to editing were evaluated, no major differences in differentiation capacity (within the tested markers) were observed between total vs. the mScarlet$^+$ population (Figures S3C and S3D). Although the post-editing cell number was approximately one-third less for 100 TU/cell than for 50 TU/cell, we selected the higher dose for insertion of the corrective *$CFTR_{2\text{-}27}$* transgene due to an increased TI frequency.

Figure 1. Evaluation of targeted insertion in primary basal cells using IDLV

(A) Schematic of the targeting protocol, including timeline of molecular and functional assessments. (B) Schematic of *CFTR*, with focused view on intron 1 at the insertion site (top) and after the insertion of the reporter (bottom). (C) Comparison of insertion efficiency measured as % mScarlet+ cells (left) and cell recovery (right) in absence or presence of AZD7648. (D and E) Insertion efficiency measured as % mScarlet+ cells (D) and cell recovery (E) in presence of AZD7648 and with increasing dose of IDLV, across three different cell donors. Mean ± SD of three independent experiments.

Efficient IDLV-mediated targeted insertion of corrective $CFTR_{2-27}$ in basal stem cells

Having demonstrated the ability to target intron 1 with an IDLV-delivered reporter construct, we next turned our attention to target insertion of the corrective $CFTR_{2-27}$ transgene preceded with an SA sequence and followed by the BGH polyA signal; R1 site-specific HAs were included to facilitate HDR-mediated insertion. The corrective $CFTR_{2-27}$ transgene was delivered via IDLV to basal cells immediately following electroporation of RNPs specific for *CFTR* intron 1 R1 (Figure 2A). PCR-amplified genomic DNA from TI-1 treated (hereafter TI-1(t)) basal cells was first analyzed via agarose electrophoresis to confirm the presence of $CFTR_{2-27}$ insertion. Evidence for the desired integration was consistent with the size of amplicons generated with various primer sets (Figure 2A; Table S2): a full insertion was detected through outside-outside PCR and both 5′ and 3′ ends were amplified with the respective inside-outside PCRs (Figure 2B; Figure S4A; Table S2). Semi-quantitative analysis was performed as previously described.[7] This method involves inserting an EcoRI site in the donor downstream of the $CFTR_{2-27}$ polyA and duplicating a forward primer sequence (#7) that is located upstream of the gRNA site, also at the 3′ end of the corrective $CFTR_{2-27}$ transgene (Figure 2A; Table S2). PCR using primers #7 and #4 amplifies unmodified, indel, and TI alleles simultaneously with similarly sized amplicons to avoid length-dependent amplification bias. Since only amplicons derived from TI alleles contain the EcoRI site, digestion of amplicons with EcoRI provides a semi-quantitative estimate of TI allele frequency. This analysis showed a high frequency of TI per allele (~66%–77%) in TI-1(t) basal cells from three CF donor cell samples (Figure 2B; Figure S4A).

To quantitatively measure the TI-1 events, we next performed next-generation sequencing (NGS) analysis utilizing the same primer set (#7/#4) but with NGS adapters (Table S2) and alleles were assigned to unmodified, small indels, or TI according to NGS read-outs (Figure S4B). Since the forward primer (#7) binds only 62 bp upstream of the gRNA cut site, alleles with large deletions that disrupt the primer-binding site or insertions that exceed the extension capacity of PCR are not amplified, thus the NGS analysis (as well as the EcoRI assay described above) likely overestimates the TI-1 efficiency. In fact, recent studies have demonstrated that large indels mediated by NHEJ can occur and have been overlooked in many analyses.[20] To account for alleles not amplified due to large deletions or insertions, we performed an EvaGreen ddPCR copy number assay using the same primer set (#7/#4) (Figure S4C). The copy number of alleles amplified by this primer set was normalized to that of a diploid reference gene (Table S2). This allowed us to estimate the fraction of alleles captured by NGS and the fraction of alleles with larger modifications that failed to amplify (Figure S4C). After correction (Figure 2C), we confirmed high levels of TI-1 (49%–56% of alleles) across three different cell donors. In all three cell donors, we also observed large modifications at frequency 28.5%–31.2% of alleles in RNP treated cells, and at frequency 17.7%–24.1% in TI-1(t) cells (Figure 2C); the reduction in large modifications upon addition of a homologous donor template is consistent with previous reports.[20,21]

While the quantification approach here benefits from unbiased amplification, the short-read NGS assay has certain limitations in detecting and categorizing large insertions in addition to the issue mentioned above. For example, our approach relies on amplification across the TI junction at only the 3′ HA and therefore cannot distinguish alleles with complete TI from those with partial integration or NHEJ at the opposite arm. Such events may be inaccurately classified as TI. To assess the frequency of these events and to more comprehensively characterize other large modification outcomes, we performed long-range PCR (primers #5/#6; Table S2) followed by single molecule, real-time sequencing (SMRT-seq) analysis and used the pipeline shown in Figure S5A. Long-range PCR is subject to length-dependent amplification bias, making it unsuitable for accurate quantification. However, when combined with the high read accuracy of SMRT-seq circular consensus sequencing (CCS), it is well-suited for detecting and mapping a wide range of editing events. For this analysis, we selected one of the F508del/F508del experiments. In basal cells treated with RNP alone, the majority (~82%) of reads were comprised of small indels or unmodified (Figure 2D). Other species were large deletions and insertions. For RNP/IDLV treated cells, the majority of integration events (64.8%) were the desired 4,720 bp precise HDR TI-1 (Figure 2D) with lower levels of partial donor insertions (11.9%) or IDLV capture (6.7%); the remaining 16.6% of SMRT-seq reads reflected unmodified alleles, indels, or larger deletions. The IDLV capture events showed diverse integration patterns such as 5′ HDR with 3′ NHEJ, 5′ NHEJ with 3′ HDR, full-length IDLV capture, and a rare instance (0.02%) of tandem head-to-tail integration of three IDLV copies (~20 kb), consistent with previously reported observations.[22] In Figure S5B, the frequency of SMRT-seq reads of various length are presented, as well as a structure of a representative insertion for each bin. Notably, many IDLV capture events retained a 5′ HDR structure, which would potentially result in expression of the full-length *CFTR* transgene from the endogenous promoter, even though the correctly targeted cassette is followed by additional IDLV-derived vector sequences; examples of this in Figure S5B include molecular species of insertion size 6,808 bp and 12,459 bp. In addition, although their combined frequency was very low, numerous IDLV capture events retained the 3′ HDR structure but not the 5′ HDR structure. Taken together, this molecular characterization provides evidence for highly efficient targeted integration of the complete $CFTR_{2-27}$ at intron 1 in airway basal cells, using a single IDLV donor without requiring selection.

We also assayed potential off-target activity in basal cells treated only with RNPs targeting the R1 site. When *in silico* predicted potential off-target sites were examined for the introduction of indels, 12 locations were identified and tested for their integrity. The NGS analysis showed no evidence of off-target activity at any of the tested sites (Figure S5C; Table S2).

Restoration of CFTR expression and function via TI-1 of corrective $CFTR_{2-27}$ transgene

For *CFTR* alleles with targeted intron 1 insertion of $CFTR_{2-27}$ (flanked at 5′ and 3′ ends by SA and BGH polyA, respectively), the intended consequence in CFTR expressing cells would be an

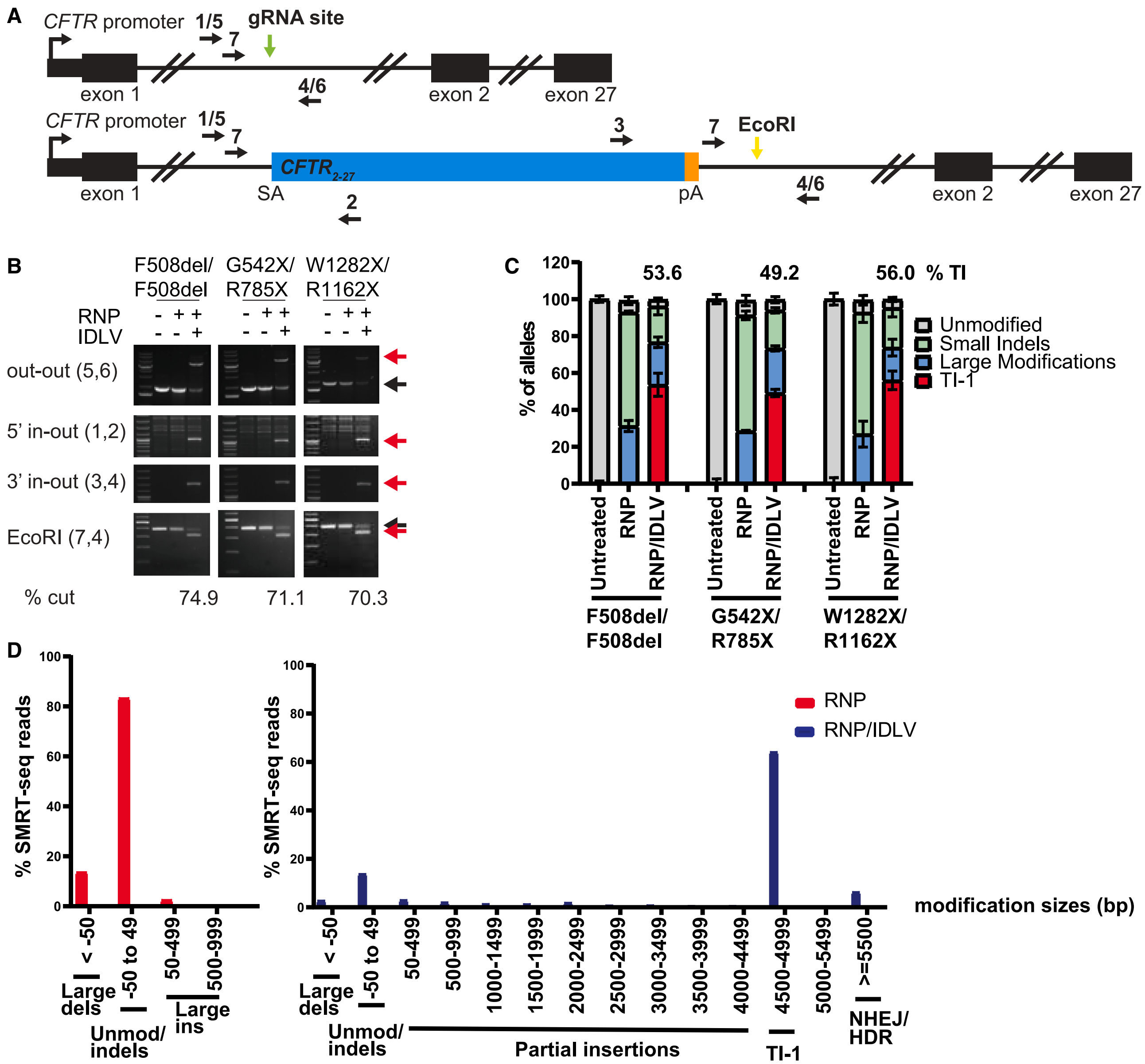

Figure 2. Evaluation of TI-1 using WT *CFTR*$_{2\text{-}27}$ transgene

(A) Schematic of *CFTR* with a focused view on intron 1 at the insertion site (top) and DNA structure after the *CFTR*$_{2\text{-}27}$ insertion (bottom). Horizontal arrows around the gRNA cut site and the inserted *CFTR*$_{2\text{-}27}$ represent the primers used for the molecular detection of the insertion. The numbers refer to the primer list in Table S2. (B) Representative agarose gels from the three cell donors, using the primer pairs shown in (A). From top to bottom: out-out PCR (primers 5 and 6), 5′ in-out PCR (primers 1 and 2), 3′ in-out PCR (primers 3 and 4), and EcoRI detection PCR (primers 7 and 4). The EcoRI detection PCR product was digested with EcoRI, generating a cleaved band specific to the TI-1 allele. % of PCR products cleaved are shown under RNP/IDLV treated samples. Red arrows indicate bands consistent with successful insertion, which are observed only in RNP/IDLV treated samples; black arrows indicate bands from unmodified or small indel alleles. (C) Summary results from deep sequencing analysis (Figure S4B) after correction based on amplicon copy number (Figure S4C). The numbers above the graph represent the fraction of alleles that, based on sequence, were identified as TI-1. Mean ± SD of three independent experiments. (D) Summary of SMRT-seq analysis from a representative experiment in F508del/F508del cells. Plots show the distribution of insertion and deletion sizes: events ranging from 50 bp deletions to 49 bp insertions are classified as unmodified or indels; deletions larger than 50 bp are classified as large deletions; and insertions larger than or equal to 50 bp are classified as large insertions. Deletion lengths are represented as negative values; therefore, values < -50 correspond to deletions larger than 50 bp. The left figure shows RNP only treated cells, and the right figure shows RNP/IDLV treated cells. In RNP/IDLV treated cells, a 4,720 bp insertion corresponds to the TI-1 allele. Large insertions between 50 and 4,499 bp reflect partial IDLV insertions, whereas insertions $\geq$5,500 bp are consistent with IDLV capture with HDR on one junction and NHEJ on the opposite junction.

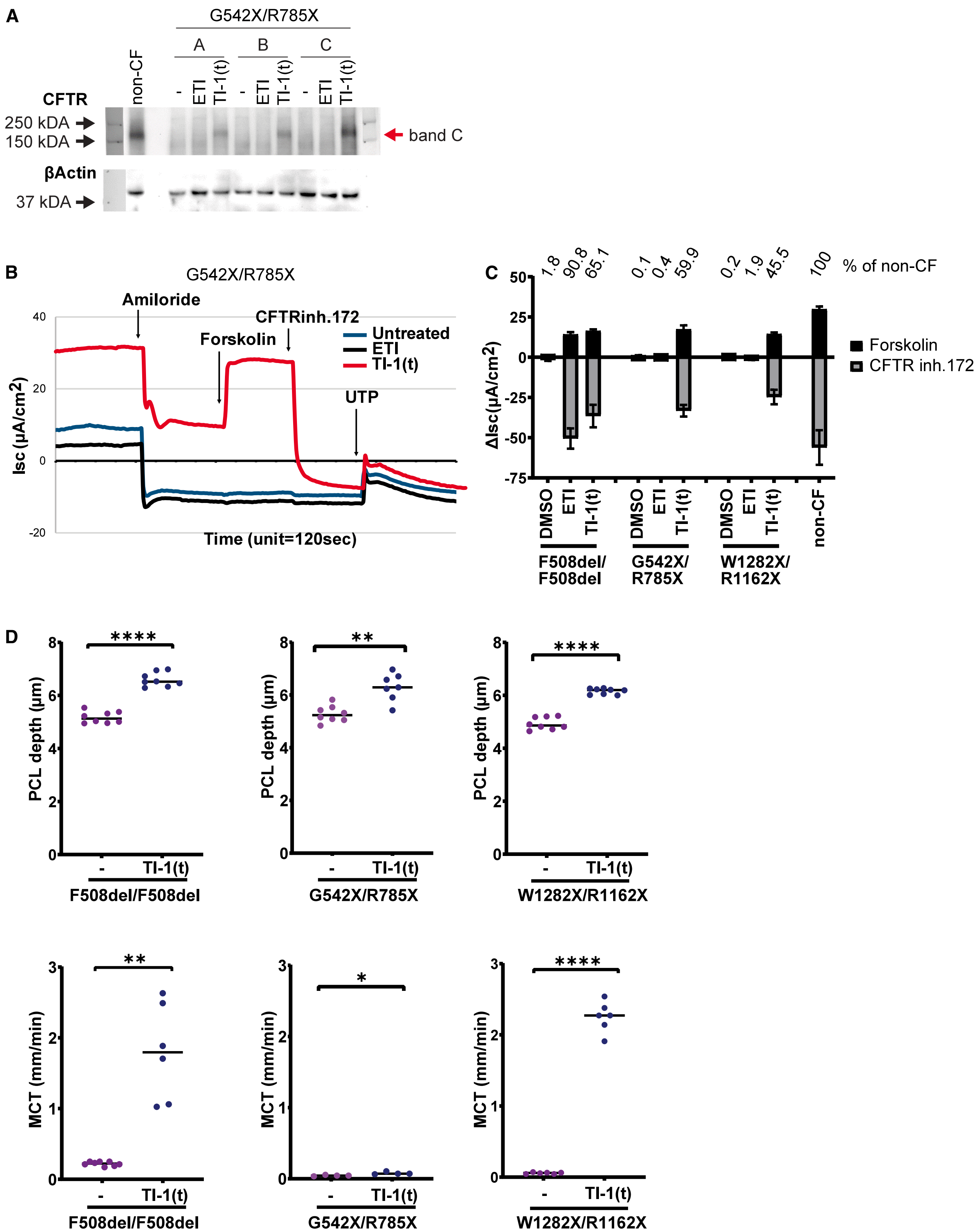

(legend on next page)

mRNA transcript, initiated by the endogenous *CFTR* promoter, that includes native exon 1, corrective *CFTR* exons 2 through 27, and polyadenylated via BGH signal sequences. Later in the "results," we provide evidence consistent with such mRNA transcripts. When translated, these mRNAs should give rise to WT CFTR proteins, able to mature through full glycosylation and migrate to the plasma membrane as functional ion channels. Furthermore, if the restored WT CFTR protein is present at sufficient frequency in the requisite airway epithelial cells, correction of CFTR-mediated ion transport defects should be a consequence.

In Figure 3A, we demonstrate that G542X/R785X basal stem cells, treated in three independent experiments with the TI-1 methodology, upon differentiation to airway epithelium under ALI conditions give rise to fully mature, glycosylated CFTR band C protein. As shown in Figure 3A, parental G542X/R785X cells do not have any detectable CFTR. TI-1(t) cells showed evidence of CFTR band C restoration, estimated by densitometry to be 53%–57% of non-CF protein levels. As an additional negative control, we treated some of the ALI inserts from the parental G542X/R785X cells with ETI 48 h prior to harvesting the cells for western blot. As expected, no CFTR protein resulted from ETI treatment (Figure 3A).

Restoration of CFTR anion transport in TI-1(t)-derived airway epithelium was assessed by Ussing chamber studies. Ussing analysis involved acute treatment with amiloride to inhibit epithelial sodium channel (ENaC), forskolin to stimulate CFTR channel opening, CFTR inhibitor-172 to block CFTR-specific chloride ion transport, and finally UTP to test epithelium integrity in CFTR-independent manner (Figure 3B). The results were reported as change in short circuit current (ΔIsc) upon stimulation with forskolin or inhibition with CFTR inhibitor-172: when three different donor cells were subjected to the TI-1 methodology we observed a functional restoration equivalent to 45.5%–65% of non-CF current (measured relative to the CFTR inhibitable current) (Figure 3C). The assay was validated by treatment with ETI as control. Parental unedited F508del/F508del ALI cultures, showing no channel activity with the DMSO control, responded with up to ~90% of non-CF current with ETI. As expected, unedited CF lines with compound heterozygous PTCs exhibited no CFTR activity either with DMSO or ETI (Figure 3C). These results confirm that the TI-1 strategy restores CFTR channel activity irrespective of the patient genotypes examined.

Finally, utilizing micro-optical coherence tomography (μOCT), we assayed features of functional restoration relevant to airway surface hydration and mucociliary clearance, both of which are clinically relevant to the CF airway phenotype. Periciliary liquid (PCL) depth, a measure of airway surface hydration, showed significant improvement in all three TI-1(t) samples compared to untreated parental cells (Figure 3D). Mucociliary transport (MCT) was significantly improved in TI-1(t) cells from both F508del/F508del and W1282X/R1162X (Figure 3D; Videos S1 and S2). ETI treatment yielded improvement in PCL depth and MCT only in F508del/F508del ALI cultures (Figure S6A). Although we did observe a statistically significant increased MCT in G542X/R785X TI-1(t) cultures, both the baseline mutant and the corrected G542X/R785X cultures exhibited MCT levels significantly reduced with respect to the other cultures. We have, on occasion in unrelated experiments, seen similar results (i.e., reduced MCT in cultures despite detectable CFTR anion channel activity) in other ALI cultures. We note that the inability of cells to mobilize mucus in a cell culture vessel can sometimes result from the formation of large mucus rafts *in vitro* that hamper the MCT assessment, independent of airway surface hydration level.

To demonstrate evidence of TI-1 functional restoration in clonal populations of corrected cells, we applied the TI-1 methodology to iPSCs derived from a CF individual homozygote for W1282X variant. Screening of colonies resulted in the identification of TI-1 CF-iPSC clones, either heterozygous or homozygous for TI-1. iPSC clones were differentiated into airway organoids (Figure S6B) that were subjected to a forskolin-induced swelling (FIS) assay that measures CFTR function as previously described.[23] As shown in Figure S6C, both heterozygote and homozygote TI-1 clones exhibited significant swelling in response to forskolin stimulation providing evidence for CFTR function compared to parental unedited W1282X/W1282X organoids. In summary, we show that TI-1 results in a mature and functional CFTR able to significantly restore ion transport function of CF airway epithelium.

Basal cells subjected to TI-1 methodology retain multipotency

CF basal cells subjected to editing were differentiated into airway epithelium in ALI culture conditions. As seen in Figure 4A, immunostaining of 4-week ALI cultures demonstrated, for both unmanipulated and TI-1(t) conditions, the presence of basal cells (P63, NGFR, and KRT5), secretory cells (SCGB1A1/CC10, MUC5AC, and MUC5B), and ciliated cells (ACT). Furthermore, scRNA-seq analysis of fully differentiated airway epithelium revealed minimal differences in cell-type composition between unedited and TI-1(t) populations—either via UMAP pattern (Figure 4B) or quantified by frequency (Figure 4C). In addition to the predominant cell types

Figure 3. TI-1 results in significant functional restoration of CFTR in TI-1-treated primary cells

(A) Western blot of CFTR and βActin (internal control) derived from ALI cultures of three independent editing experiments with G542X/R785X shown in Figure 2C. (B) Representative traces of Ussing chamber assay performed after 4 weeks of ALI culture. Each trace represents one experimental condition of either TI-1 or 48 h pre-treatment with vehicle control (DMSO) or ETI. Vertical arrows represent acute treatments performed during the recording. (C) Summary of the analysis in (B) from three different cell donors, compared to non-CF. The number above the graph represents the % of non-CF current measured upon CFTR inhibitor-172 treatment. Mean ± SD of three independent experiments. (D) Summary of μOCT measurements of PCL (top) and MCT (bottom) of 4 weeks ALI cultures. Individual measurements of 4 technical replicates (1–2 images each) are shown centered on the median. *$p < 0.05$; **$p < 0.01$; ****$p < 0.0001$.

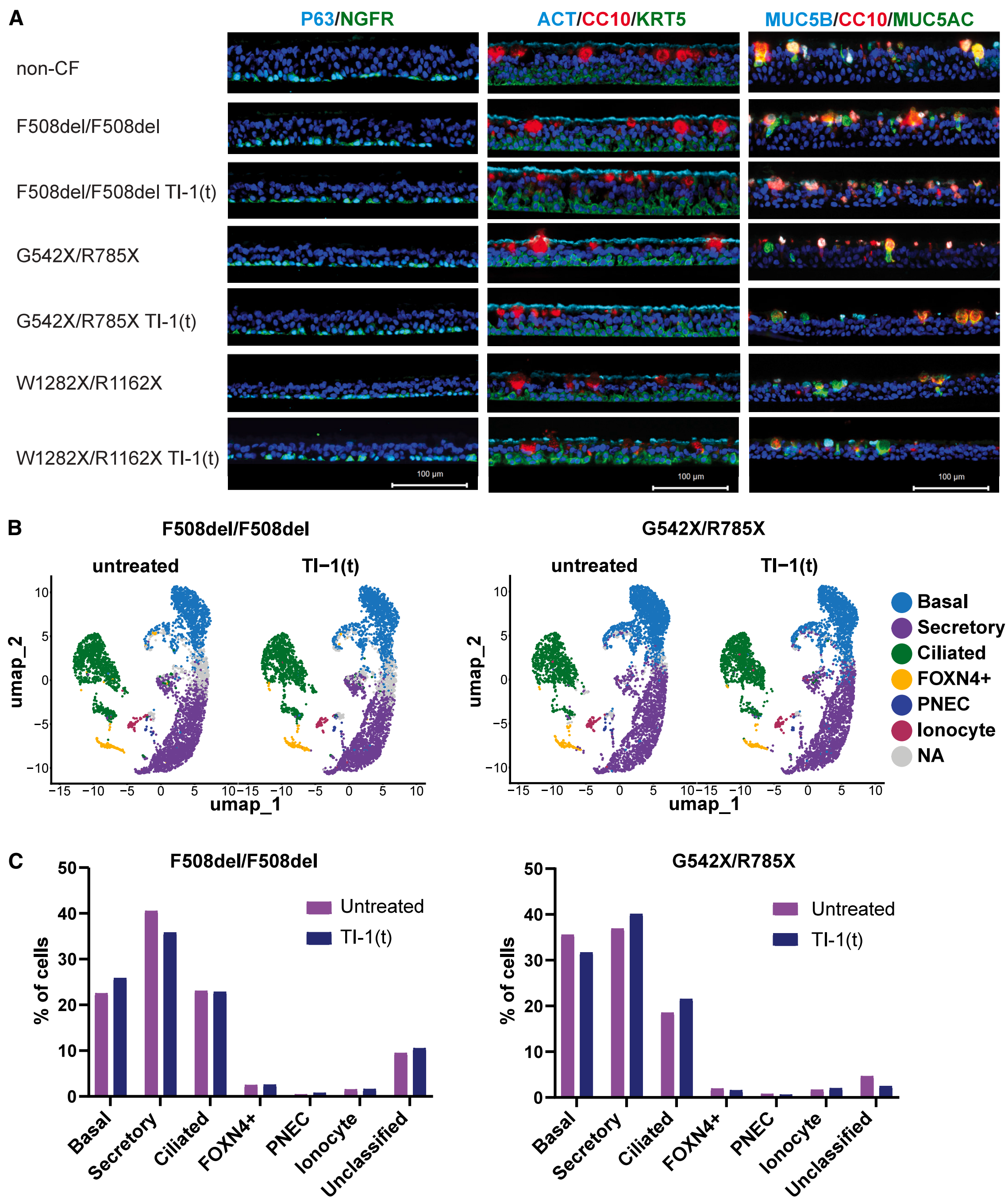

Figure 4. TI-1 does not alter the differentiation capacity of TI-1-treated primary basal stem cells

(A) Immunostaining of 4 weeks ALI cultures stained with markers for basal (P63, NGFR, KRT5), secretory (SCGB1A1/CC10, MUC5B, MUC5AC), and ciliated cells (ACT). Scale bars, 100 μm. (B) UMAPs of cell-type clusters comparing untreated vs. TI-1(t) cells for two cell donors after 4 weeks of ALI culture. (C) Quantification of each cell type in (B) for either untreated or TI-1(t) cells.

(basal, secretory, and ciliated cells), scRNA-seq also identified rare cell populations (FOXN4+, pulmonary neuroendocrine cells [PNEC], and ionocytes) and unclassified populations that were not significantly affected by the manipulation (Figures 4B and 4C; Figure S7A). Moreover, differential gene expression analysis, comparing TI-1(t) with unmanipulated cells, revealed that the TI-1 methodology caused only a small number of genes to be differentially expressed (Figure S7B).

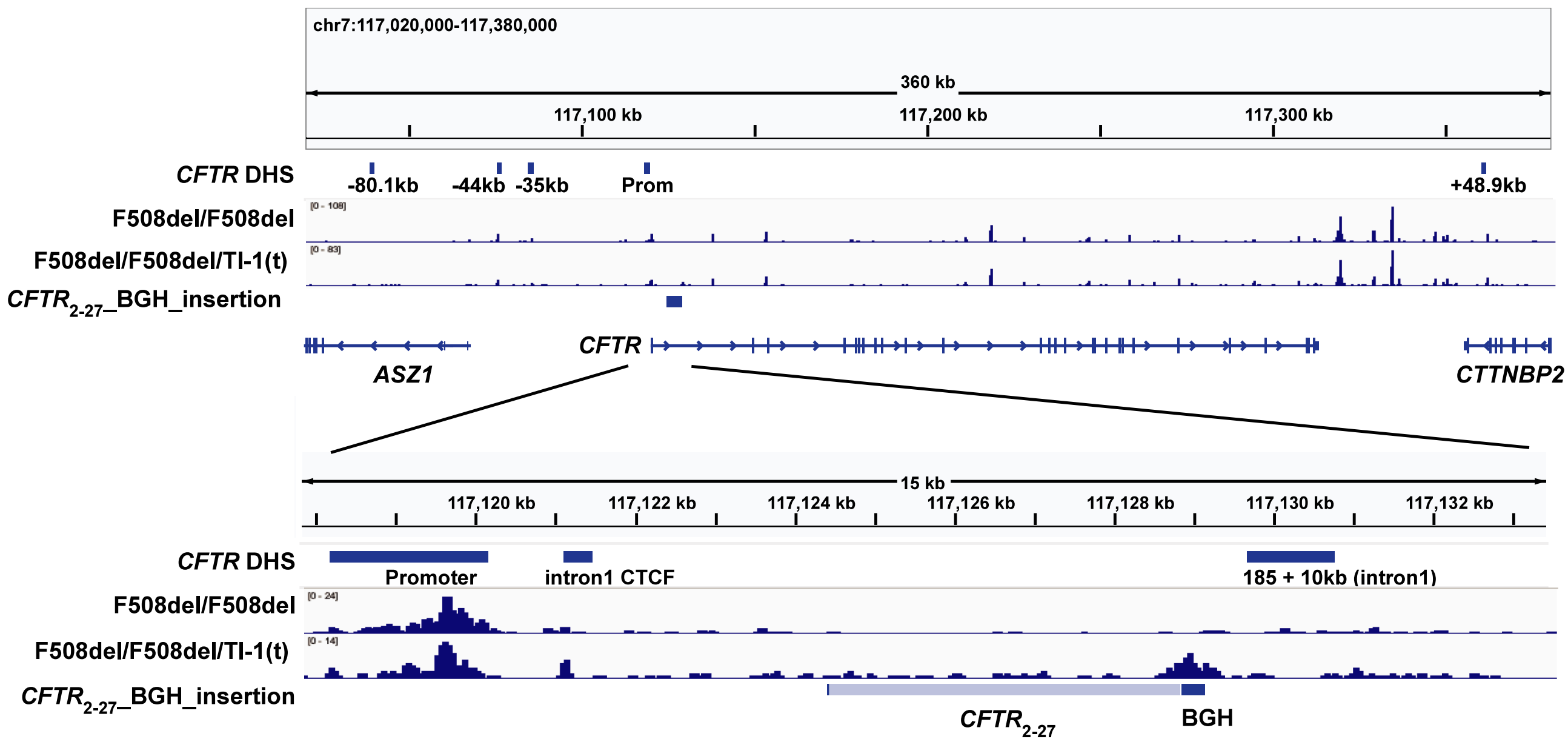

Figure 5. TI-1 does not alter ATAC-seq characterization of the *CFTR* locus
ATAC-seq with focused view on *CFTR* and its regulatory elements. Each peak represents a region of open chromatin. Below, a zoomed-in view of the insertion site.

Insertion of *CFTR*$_{2-27}$ TI-1 has minimal impact on CFTR open chromatin profile

Our rationale for integrating corrective *CFTR*$_{2-27}$ transgene into the endogenous *CFTR* locus was to exploit the native chromatin architecture, promoter activity, and *cis* regulatory elements (CREs) to achieve appropriate cell-type-specific expression. Analysis of open chromatin status via ATAC-seq is a valuable tool for identifying location and activity of relevant tissue- and cell-type-specific regulatory elements in the *CFTR* locus and whether they are significantly impacted by the insertion of the corrective *CFTR*$_{2-27}$ transgene. This analysis, performed on F508del/F508del TI-1(t) (53.6% of alleles TI-1) airway epithelium in comparison with F508del/F508del, showed that the *CFTR* locus open chromatin sites were not significantly altered (Figure 5). For example, previously identified sites −44 kb, −35 kb, promoter, as well as intron 1 specific sites (intron 1 CTCF, 185 + 10 kb) were minimally affected. We note the appearance of a small peak of unknown significance aligned with the BGH polyA sequence, which might reflect a genuine alteration in local chromatin structure or represent an artifact of sequence alignment to other gene(s) expressed in airways (e.g., ORAI2). These results are consistent with our goal for the inserted TI-1 sequences to not significantly alter *CFTR* chromatin context and instead be subject to the normal regulation of *CFTR* expression.

Cell-type-specific expression pattern of corrective *CFTR*$_{2-27}$ replicates that of native CFTR

To determine whether the TI-1 methodology in basal cells altered the cell-type-specific pattern of *CFTR* expression in the derived airway epithelium, we compared, via scRNA-seq, the pattern of *CFTR* expression in unedited-vs. TI-1(t)-derived airway epithelium of genotype F508del/F508del or G542X/R785X. For this analysis, we first considered the expression of total *CFTR* mRNA (irrespective of whether native or corrected). As shown in Figure 6A, *CFTR* mRNA expressing cells were most evident in secretory and ionocyte populations, and to a significantly lesser extent in basal and ciliated cells; in both genotypes, this pattern was quite similar for unedited- and TI-1(t)-derived airway epithelia as highlighted also in violin plots (Figure 6B) depicting the level of total *CFTR* expression per cell. As expected, ionocytes, on average, exhibited the highest level of *CFTR* expression per cell (mean values of 2.34 and 1.8 for F508del/F508del untreated and TI-1(t), respectively), with secretory cells expressing moderate levels per cell (1.11, 0.98), followed by basal (0.87, 0.84) and ciliated (0.89, 0.75) cells. Any differences between unmanipulated cells and cells undergoing the TI-1 methodology appeared to be relatively minor. When the total *CFTR* expression in the various populations of cells was examined, secretory cells were responsible for the vast majority of *CFTR* (e.g., 65.9% for F508del/F508del TI-1(t)), followed by ionocytes (14.8%), and then basal (4.9%) and ciliated (2.6%) cells (Figure 6C).

The use of WT sequence for the corrective *CFTR*$_{2-27}$ transgene rendered the assignment of the majority of *CFTR* scRNA-seq reads to either native or corrected species non-trivial due to excessive internal priming occurring in that gene (binding of oligo-dT primers to nearly homopolymer A stretches distinct from polyA tails). Nevertheless, at specific exons we were able to utilize unique features distinguishing endogenous and corrected mRNA transcripts. For example, the corrective *CFTR*$_{2-27}$ transgene includes an intentional T>C silent SNP in exon 7 corresponding to position chr7:177,536,608 (GRCh38/hg38; amino acid N268) whereas the

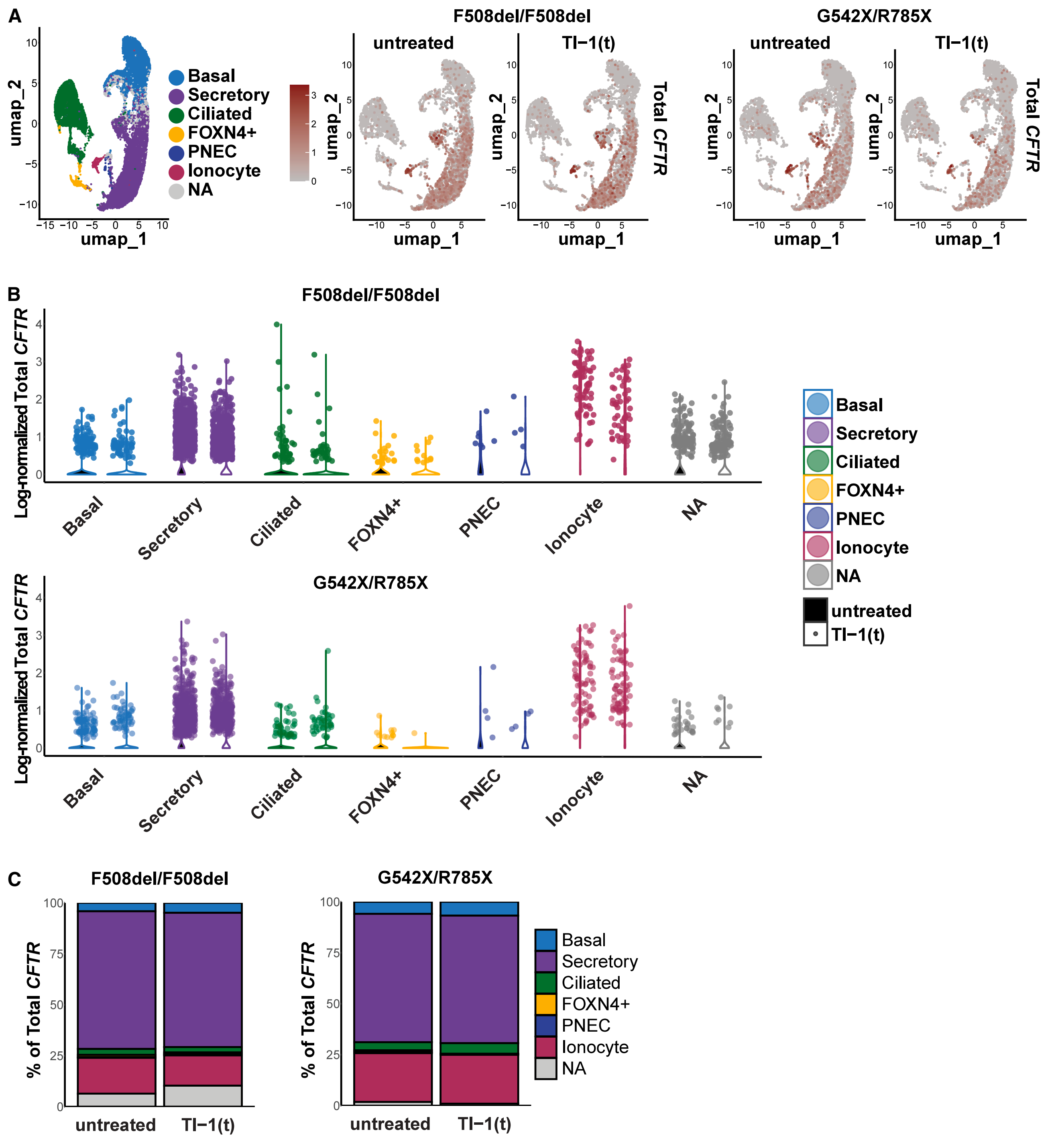

Figure 6. TI-1 does not alter *CFTR* transcriptional regulation in differentiated ALI cultures

(A) Left: UMAP showing the identification of cell clusters across all samples. Middle and Right: UMAPs showing the distribution of total *CFTR* (mutant plus corrected) in each cell cluster and comparing untreated vs. TI-1(t) cells for two cell donors. (B) Violin plots showing log-normalized total *CFTR* expression level across each cluster in A, with comparison of untreated vs. TI-1(t) cells. (C) Distribution of total *CFTR* expression across the clusters in (A).

A

SNP T>C in exon7 (chr7:177,536,608)	total # of reads	# of reads (T)	# of reads (C)	% of reads (T)	% of reads (C)
F508del/F508del	437	435	2	100	0
F508del/F508del TI-1(t)	186	98	87	53	47
G542X/R785X	105	105	0	100	0
G542X/R785X TI-1(t)	167	38	129	23	77

c.1522_1524del (p.Phe508del)	total # of reads	# of reads (mutant)	# of reads (WT)	% of reads (mutant)	% of reads (WT)
F508del/F508del	327	325	0	99	0
F508del/F508del TI-1(t)	452	212	240	47	53
**c.1624G>T (p.Gly542Ter)**	**total # of reads**	**# of reads (mutant)**	**# of reads (WT)**	**% of reads (mutant)**	**% of reads (WT)**
G542X/R785X	112	64	47	57	42
G542X/R785X TI-1(t)	262	23	238	9	91
**c.2353C>T (p.Arg785Ter)**	**total # of reads**	**# of reads (mutant)**	**# of reads (WT)**	**% of reads (mutant)**	**% of reads (WT)**
G542X/R785X	21	11	10	52	48
G542X/R785X TI-1(t)	21	3	18	14	86

B

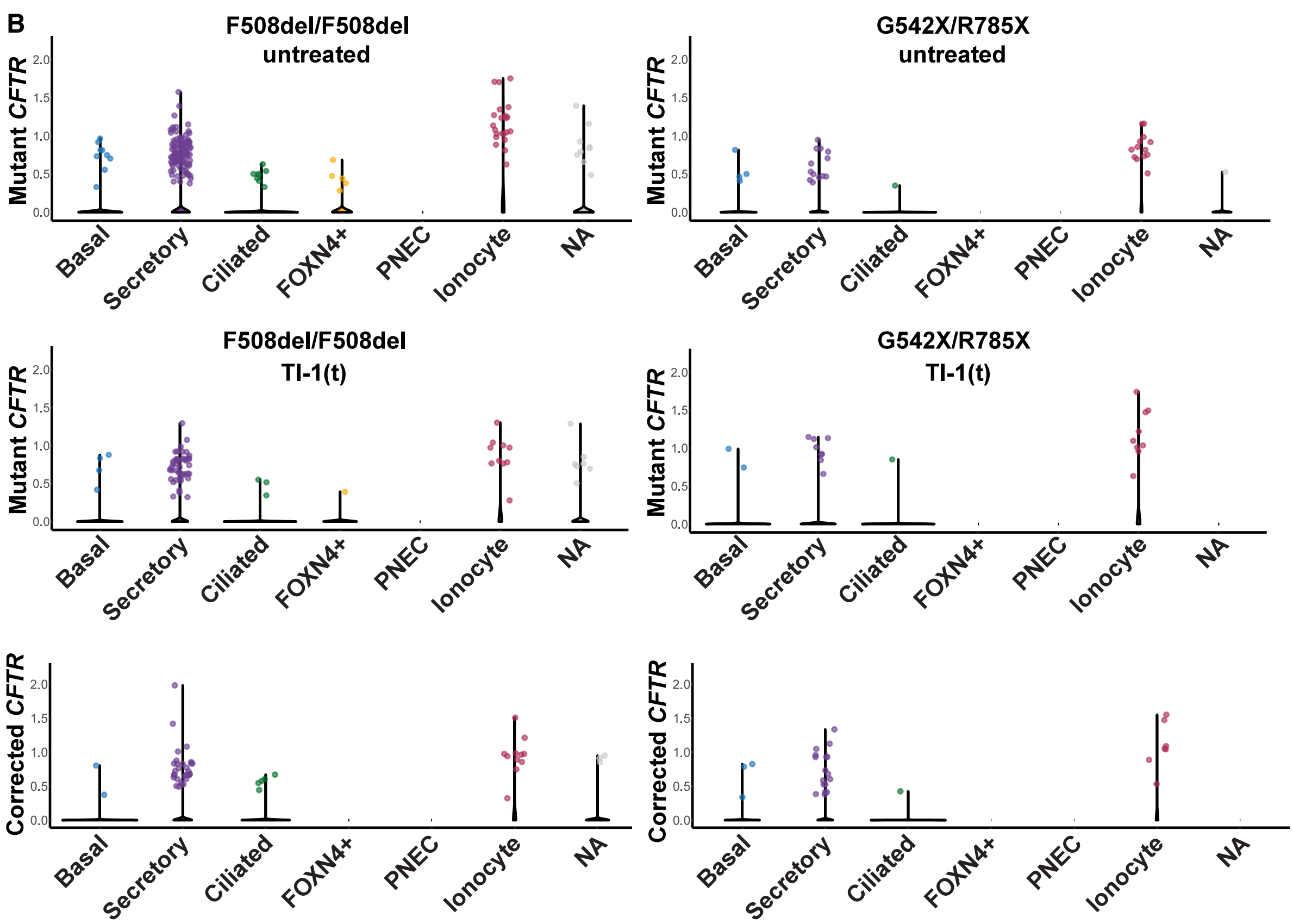

(legend on next page)

endogenous base is a T. As shown in Figure 7A and Figure S8A, in F508del/F508del samples the reads aligning over that position carry the corrected "C" allele at 47% frequency in TI-1(t) cells whereas in the unedited sample the native "T" allele is observed at 100% frequency. We were also able to distinguish corrected from mutant mRNAs by examining reads aligning over the location of the F508del variant in exon 11: in TI-1(t) cells, the reads with WT sequence (no deletion) were observed at 53% frequency and F508del reads at 47% frequency (Figure 7A; Figure S8A). These results demonstrate that the "total" *CFTR* expression in the TI-1(t) F508del/F508del cells is a mixture of corrected and mutant/native *CFTR* mRNA at roughly equal proportions. This is consistent with the TI-1 frequency in F508del/F508del basal stem cells being ∼54% on a per DNA allele basis (Figure 2C).

While G542X and R785X transcripts are subject to degradation by the nonsense-mediated decay (NMD) pathway, *CFTR* mRNA derived from such mutations has been observed in airway cells at a level of ∼20%–30% of WT mRNA.[24,25] In G542X/R785X samples, we were indeed able to observe expression of mutant mRNA. 100% of reads at exon 7 T>C SNP location showed the endogenous T in unedited cells, whereas in the TI-1(t) cells the corrected "C" allele was observed at 77% (Figure 7A; Figure S8A). This high frequency of corrected alleles at the mRNA level is consistent with endogenous PTC-carrying mRNA being subject to NMD, while corrected mRNA transcripts, in principle, should not be subject to this pathway. Thus, the increased abundance of the corrected transcript (77%) vs. the frequency of corrected TI-1 alleles (∼49%) at the genomic DNA level (Figure 2C) is likely due to the increased stability of such transcript vs. the mutant. Further evidence for the preferential stability of corrected mRNA comes from analysis of WT (e.g., G542 or R785) vs. mutant (G542X or R785X) sequences in the scRNA-seq reads. Unedited G542X/R785X cells revealed that 42% of the reads covering the codon for amino acid 542 contained WT sequence (G542) and 48% of the reads overlapping with codon for Arg785 contained the WT sequence (R785). This is close to the expected 50:50 distribution expected at the DNA level based on compound heterozygosity in this PwCF and would be consistent with transcripts from both alleles being similarly (un)stable. Sequence analysis from TI-1(t) G542X/R785X cells exhibited 91% G542 reads and 86% R785 reads (Figure 7A; Figure S8A). This significant simultaneous increase in frequency of WT reads in TI-1(t) cells (relative to unedited) at both regions is consistent with TI-1 correction at the genomic DNA level together with corrected transcripts no longer being subject to NMD. Taken together, the analysis of TI-1(t) F508del/F508del and G542X/R785X cells clearly demonstrates that TI-1 editing resulted in robust expression of the corrected *CFTR* mRNAs.

The relative abundance of endogenous vs. corrected transcripts could also be independently verified based on the following: polyA-selected endogenous *CFTR* transcripts include the native 3′ UTR, whereas the corrected mRNAs terminate with the BGH polyA. The 10X HT 3′ assay generates read sequences within ∼300–400 bp of the oligo dT primer binding site, making *CFTR* reads that originate from binding at the polyA tails and align within the respective UTRs fully distinguishable. Furthermore, the native transcripts result in very low residual coverage in exons 26–27 (due to native UTR length of ∼1.5 kb, Figure S8B), while the short BGH polyA sequence (AATAAA sequence starting at 95 bp from exon 27) allows for a sizable portion of polyA-primed reads originating from corrected transcripts mapping to exons 26–27 (Figure S8B). This provides yet another way of confirming expression of corrected transcript in TI-1(t) cells.

We further used the 3′ end distinguishing features of endogenous mutant vs. corrected transcripts to evaluate the cell-type-specific pattern of *CFTR* expression (Figure 7B). Although the total number of reads per cell type was not sufficient for quantification, we did see the expected expression primarily in the secretory and ionocyte populations and to a lesser extent in basal and ciliated cells when endogenous *CFTR* expression was examined in unedited F508del/F508del and G542X/R785X ALI cultures (Figure 7B). A similar pattern of endogenous *CFTR* expression was also seen in TI-1(t) F508del/F508del and G542X/R785X cells (Figure 7B). Significantly, expression of the corrected *CFTR* mRNA observed across those cell types in the TI-1(t) cells (Figure 7B) closely replicated the cell-type-specific pattern of endogenous *CFTR* expression.

DISCUSSION

We report here the development of a highly efficient *CFTR* intron 1 targeted integration approach in primary CF airway basal stem cells that, without selection for edited cells, restores CFTR expression, and function in the derived airway epithelium. Functional rescue was confirmed by the improvement of clinically relevant phenotypes such as mucus hydration and ion transport, supporting the therapeutic potential of this approach. A query of the Cystic Fibrosis Foundation Patient Registry (CFFPR), which tracks over 34,000 PwCF, did not yield a single individual with two confirmed variants upstream of the intron 1 (R1) insertion site. Given the heterogeneity of variant frequencies across ancestries and geographies, we conservatively estimate that greater than 99.8% of PwCF could benefit from our proposed therapeutic approach, i.e., this approach is almost completely mutation-agnostic. These results are applicable to $CFTR_{2\text{-}27}$ integration via other editing methodologies and, if appropriately vectored, for direct *in vivo* correction.

Figure 7. *CFTR* mRNA derived from corrective WT $CFTR_{2\text{-}27}$ transgene is expressed with comparable cell-type distribution and expression level as the native mutant *CFTR*

(A) Number and percentage of aligned reads for the regions of interest that distinguish mutant vs. corrected transcripts. (B) Violin plots of untreated (top) or TI-1(t) cells (middle and bottom) with log-normalized *CFTR* expression level by cluster identified in Figure 6A. Mutant *CFTR* plots include reads that align with the native *CFTR* 3′ UTR. Corrected *CFTR* plots include reads derived from exons 26–27 (excluding 3′ UTR) (Figure S7B) plus the BGH polyA.

We first utilized a chimeric SA-*CFTR*$_{2\text{-}4}$-luciferase-polyA reporter to survey various locations in *CFTR* intron 1 and determine what flexibility there was for selecting potential insertion sites for *CFTR*$_{2\text{-}27}$. We deliberately chose to target insertion in intron vs. exon sequences for several reasons: (1) to maintain a measure of exon/intron structure shown in mice to increase the level of transgene expression[26]; (2) to avoid introduction of CRISPR-induced indels in exon sequences that could result in generation of additional CFTR mutations and disruption; (3) to preserve responsiveness of the native mutant alleles to modulators in the event of indels instead of corrective *CFTR*$_{2\text{-}27}$ insertion. For example, ALI cultures derived from RNP alone treated F508del/F508del basal cells (~90% alleles with either small or large intron 1 indels, Figures 2C and 2D) were still highly responsive to ETI (data not shown). Should small molecule readthrough drugs for *CFTR* PTC variants eventually become available, residual CRISPR-induced indels in *CFTR* intron 1 from prior TI-1 treatment should not, in principle, adversely affect responsiveness of such alleles.

The number of insertion sites examined was rather limited, due both to pre-screening (e.g., for repeat sequences and SNPs that could interfere with HDR-mediated integration) and simultaneous evaluation of various polyA sequences. Nonetheless, the results suggest freedom in selecting sites that may be efficiently targeted and give rise to corrected CFTR expression. This flexibility in selecting sites may also apply to different insertion methods (e.g., CRISPR-associated transposases)[27] that have different requirements for recognition/guide sequences. It is possible that an examination of additional insertion locations could reveal sites yielding a greater level of expression or, alternatively, absence of expression. In future studies, we will examine ways in which the addition of mini-introns, choice of SA and 3′ UTR sequences, and perhaps codon optimization may influence expression of the corrective *CFTR*$_{2\text{-}27}$ transgene (Yan et al., North American Cystic Fibrosis Conference, 2025). In our prior TI-8 report,[7] we did observe a level of splicing across the corrective transgene to the downstream mutant native sequences. Whether this occurs for TI-1 insertions will be a subject for future study. Our results utilizing *CFTR*$_{2\text{-}27}$ with neither additional intronic sequences nor native *CFTR* exon 27 3′ UTR sequences would suggest that these elements are not required for retention of appropriate cell-type-specific expression in the airway epithelium. However, this requires further examination, not only in the airway, but also in other CFTR-expressing cell types in affected tissues/organs such as the pancreas and intestine.

The present study included a detailed molecular characterization of the consequences—both on target and off target—resulting from the TI-1 editing procedure. As described in the "results" and presented in Figure S5B, although the majority of integrated events were of the desired HDR-mediated TI, there were also a range of other species present at significantly lower frequency including, for example, partial transgene insertion, transgene concatemers, NHEJ capture at the 5′ end with HDR-mediated integration at the 3′ end, and HDR at the 5′ end and NHEJ at the other. In principle, this last class of insertion events would potentially be capable of corrective CFTR expression as long as SA, *CFTR*$_{2\text{-}27}$, and polyA are intact. This diversity of insertion events is not a unique feature of IDLV donor delivery but has been seen for AAV delivery as well.[28] The impact of such insertion events within *CFTR* intron 1 remains to be evaluated, for example, in clonal populations of cells or in a relevant animal transplant model.

A significant impetus for this strategy targeting insertion of the *CFTR*$_{2\text{-}27}$ sequences into the endogenous *CFTR* locus (as opposed to the full *CFTR* transgene integrated randomly or at a safe harbor locus, or maintained in an unintegrated state) was to replicate the exquisite cell-type-specific expression pattern of the native *CFTR* with a corrective transgene. The ATAC seq analysis strongly suggested that TI-1 preserved the location of open chromatin at sequences known to play key roles regulating *CFTR* transcription. Furthermore, scRNA-seq analysis of airway epithelium differentiated from TI-1(t) basal stem cells demonstrates that cell-type-specific expression patterns of the corrective transgene and the native *CFTR* gene are highly similar. Although this was expected to be the case for single mutation-specific editing (e.g., via base editing or prime editing) in which only the mutant bases are replaced with the correct ones without altering the rest of the endogenous *CFTR* locus, it was not known a priori whether targeted integration of such a large *CFTR* transgene into the native *CFTR* locus would necessarily yield the same result. The ATAC-seq and scRNA-seq results indicate that *CFTR* regulation is not affected by the targeted integration procedure and that the native *CFTR* promoter and regulatory sequences are sufficient to maintain appropriately regulated expression of *CFTR*$_{2\text{-}27}$ in the airway.

Through this study, we provide important evidence that targeting integration of corrective *CFTR* transgene in intron 1 can correct the fundamental cause of CF in a physiological manner and has a potential to attenuate CF lung pathophysiology. We first demonstrated restoration of CFTR protein and functional channel activity at levels approximately 53%–57% (CFTR protein) and 45%–65% (CFTR current), of our non-CF control. This quantitative level of restoration roughly matches the frequency of *CFTR* DNA alleles exhibiting TI-1 in basal cells (49%–56%); furthermore, in correction of the F508del/F508del cells, we observed corrected mRNA reads at 53% of total at the F508del position and 47% of total at the T>C SNP position. Since our TI-1(t) cells, being unselected for only corrected cells, are a mixture of homozygous TI-1, heterozygous TI-1, indel containing, and unmodified cells, it is difficult to predict precisely what level of functional correction would be expected to result from ~50% of *CFTR* genomic alleles exhibiting TI-1. Quantitative comparisons with WT CFTR function are further complicated by the considerable variability in CFTR anion channel activity observed in primary human bronchial epithelial cells, both among non-CF donors and among individuals with CF carrying the same genotype. In any case, we note that the level of TI-1 anion channel restoration observed in this study, as a function of correction efficiency, is consistent with that previously seen in site-specific correction of

F508del primary human bronchial epithelial cells both by our group[7] and Vaidyanathan et al.[29]

In addition to restored channel activity, we assayed restored CFTR function via μOCT imaging. One of the hallmark CF phenotypes in lungs is the inability of cilia to clear mucus from the airway lumen.[30] With μOCT images and measurements, we confirmed that hydration of the mucus layer produced by CF primary basal cell-derived airway epithelium was significantly improved upon editing, as judged by an increased capacity of cilia to mobilize secretions. In previous studies evaluating the effect of modulator therapies on *CFTR* variants, an increase of PCL depth from ∼4 to 6 μm or more was observed using *in vitro* models[31] as well as in longitudinal studies in PwCF.[32] This magnitude of response has been found to correlate with levels of mucus layer parameters that are clinically meaningful.[31,32] Our findings therefore suggest that insertion of $CFTR_{2\text{-}27}$ in CFTR intron 1 has the potential to yield clinically relevant benefits.

Recent reports by Vaidyanathan et al. have demonstrated successful rescue of CFTR expression and function by co-delivery of two AAVs that include the full *CFTR* transgene split into two fragments.[33] Although successful recombination between the two AAVs in transduced cells was a relatively low frequency event, the incorporation of a selectable marker in one of the AAVs allowed for high level enrichment of the desired complete *CFTR*. The end product of the targeted insertion was an intron-less construct directly linking the upstream regulatory sequences and promoter with *CFTR* exons 1–27, and terminating with a BGH polyA sequence connected to a constitutively expressed *PGK-CD19-SV40* cassette for selection. Due to the selection step, this strategy is applicable primarily to *ex vivo* correction of CF primary cells followed by autologous transplantation. Although we have similarly focused our experimental work on *ex vivo* correction of CF primary airway basal stem cells, the development of our strategy has been strongly influenced by recognition that the approach must be consistent with eventual *in vivo* delivery—either directly to the airways or systemically. This led to an emphasis on single vector delivery without the need for enrichment of corrected cells. Nonetheless, translation of an *ex vivo* approach (that currently entails separate delivery of RNP and donor to cycling basal cells) to an *in vivo* editing therapeutic that has as its target predominantly quiescent basal cells within a pseudostratified airway epithelium, will require significant development. Perhaps foremost among the challenges will be the efficient non-viral or viral co-delivery of various editing components required for site-specific targeted insertion of the corrective transgene. Furthermore, it is very possible that future targeted insertion technologies will not involve introduction of a dsDNA break (which incurs the risk of extended deletions or translocations and likely increases the incidence of NHEJ-mediated capture events). Rather than utilizing HDR, editing tools also active in quiescent, non-dividing cells such as PASTE (programmable addition via site-specific targeting elements),[34] PASSIGE (prime-editing-assisted site-specific integrase gene editing),[35] or CRISPR-associated transposases[27] may offer significant advantages. At the present time, it is difficult to determine which approach (direct *in vivo* editing targeting basal cells or *ex vivo* basal cell editing followed by autologous transplantation) will prove to be the most tractable approach to achieve a cure for CF lung disease.

Although this study focused exclusively on the CF airway, our targeted integration approach is also designed with the goal of restoring CFTR expression and function in other CF-affected organs such as pancreas and intestine. However, this would require validating that intron 1 targeting is also consistent with the desired physiological CFTR expression in other tissues. While our initial goal using this strategy will be to provide a treatment to those individuals who do not benefit from ETI or similarly developed modulator therapies, once demonstrated as safe and effective in such PwCF, an eventual goal would be to bring a long-term treatment or a cure from one-time treatment to all PwCF.

MATERIALS AND METHODS

Cell culture of primary airway basal cells

Primary airway basal stem cells (BCs), coded as KK003K (F508del/F508del CFTR; p.Phe508del),[36] KKD023N (G542X/R785X CFTR; p.Gly542Ter/p.Arg785Ter),[36] KKD030O (W1282X/R1162X CFTR; p.Trp1282Ter/p.Arg1162Ter),[36] and DD023J (non-CF), were provided by Dr. Scott Randell from the Marsico Lung Institute at University of North Carolina at Chapel Hill. The UAB IRB has reviewed and determined the study is considered not Human Subjects Research (IRB-300012957). BCs were cultured as previously described.[7,8] Briefly, cells were maintained on 804G-conditioned medium-coated plates and fed with PneumaCult-Ex Plus (#05040, Stemcell technologies, Vancouver, Canada) supplemented with 1 μM A83-01, 1 μM DMH1, and 10 μM Y27632 (#2939/10; #4126/10; #1254/50; Tocris Bioscience, Bristol, UK), hereafter PSY. BCs were routinely passaged by treating 0.05% Trypsin-EDTA (#25300062, Thermo Fisher Scientific, Waltham, MA, USA) for 5 min at 37°C. Trypsin was neutralized with 10% fetal bovine serum (FBS, #A5670701, Thermo Fisher Scientific) in IMDM (#10-016-CV, Corning, Corning, NY, USA), spun at 300 g for 5 min, and expanded at 1:10 ratio or counted for editing experiments.

Clonal isolation of primary airway basal cells

Primary BCs were detached and resuspended in FACS buffer containing PBS (#21-040-CV, Corning), 2% FBS, 25 mM HEPES, and 2 mM EDTA (#H0887, #1556487, Thermo Fisher Scientific), and filtered through 40 μm strainer. Propidium iodide (#421301, Biolegend, San Diego, CA, USA) was used for dead/live staining. Live single cells were sorted at 1 event/well in a 96 well/plate in presence of irradiated 3T3-J2 feeder (kindly provided by Dr. Scott Randell from the Marsico Lung Institute at University of North Carolina at Chapel Hill) and PSY, using single cells mode on BD FACSMelody Cell Sorter (BD Biosciences, Franklin Lanes, NJ, USA) at the Flow Cytometry Service Center at the University of Texas Health Science Center at Houston. Four plates were initially seeded for each clonal experiment and cultured till a well was confluent. Each growing clone was passaged to one well of 24 well/plate coated with 804-G conditioned medium as described above and cultured till confluent. Subsequently, each

growing clone was passaged into one 6 cm dish, followed by one 10 cm dish. Clones that reached at least the 6 cm dish step, were tested by PCR for evidence of integration. For some clones that stopped growing at the 6 cm dish step, only a partial characterization was performed due to a limited amount of genomic DNA collected.

Donor DNA generation and virus production

Plasmid DNA carrying the chimeric reporter donor was generated using pENTR2331-CFTR (#BC156254, Transomic Technolgies, Huntsville, AL, USA), pCDH CMV Nluc-P2A-CopGFP-T2A-puro (#73037, Addgene, Watertown, MA, USA) and in-house pDNAs as templates for PCR or enzyme digestions. Genomic DNA from donor BCs was used to amplify the HAs. Each element was subcloned through In-Fusion (#638952, Takara, San Jose, CA, USA). The final donor was subcloned into AAV-2 ITR-containing plasmid backbone (pAAV2-MCS from VPK-410, Cell Biolabs, San Diego, CA, USA) or HIV LTR; cPPT/CTS; RRE; HIV-1-Ψ-containing backbone (#78852, Addgene) through enzyme ligation with T4 ligase (#M0202S, New England Biolabs, Ipswich, MA, USA) or In-Fusion. $CFTR_{2\text{-}27}$-BGH was generated using pENTR2331-CFTR as template for $CFTR_{2\text{-}27}$ cDNA, while BGH was amplified with PCR using one of the BGH-containing reporter pDNA. The final donor was subcloned into pAAV2 or HIV backbone. Recombinant AAV-6 vectors were generated and titered by Vigene Biosciences (Rockville, MD, USA). Recombinant IDLV vectors were generated and titered by the University of Iowa Viral Vector Core (https://vectorcore.medicine.uiowa.edu/). IDLV titration was performed by transducing HT1080 cells which were then harvested after 72 h for DNA isolation. ddPCR on isolated DNA was performed to quantify the lentiviral genome from successfully transduced particles. Results were then normalized to a control LV-GFP whose titer was also measured by flow cytometry. The resulting measure was defined as titer unit (TU)/mL.

Target site screening

In order to identify potential target sites within intron 1, UCSC Genome browser GRCh/hg38 (https://genome.ucsc.edu/) was used to exclude repetitive regions or hotspots for common SNPs. Repetitive regions were evaluated by turning on the "RepeatMasker" track located in the "Repeats" tab. SNPs were evaluated by using the "dbSNP 155" track in the "Variation" tab. Lastly, to avoid proximity to regulatory regions, "ENCODE cCREs" track from the "Regulation" tab was used.

Regions selected for gRNA and HA design were sequenced in airway basal cells from two of the CF donors for validation. Only the F1 site showed SNPs in the HA region that are reported in CF population.

Gene editing in CF primary airway basal stem cells

BCs from passages 2 through 4 were harvested as described above. Editing was performed through electroporation of Cas9/gRNA complexes in the form of RNPs using ECM 830 electroporator (BTX Technologies, Hawthorne, NY, USA), followed by viral delivery of the donor. For each BTX 2 mm cuvette (#45-0125, BTX Technologies), 400,000 cells were resuspended in 100 μL of BTX solution (#45-0805, BTX Technologies) previously equilibrated at room temperature, and treated with 24 μg of Alt-R S.p. HiFi Cas9 Nuclease V3 protein (#1081060, Integrated DNA Technologies, Newark, NJ, USA) complexed with 12.4 μg of custom designed single gRNA (Integrated DNA Technologies) (see Table S1 for the sequences). BCs were electroporated with BTX electroporator at the following settings: 250 V, 5 ms, single pulse. Immediately after electroporation, 100,000 cells were used for each virus treatment.

If AAV-6 was used to deliver the donor DNA, BCs were exposed to a dose of 100,000–500,000 vg/cell (viral genome/cell) for 20 min in a 1.5 mL tube at 37°C. After the initial incubation, BCs were plated in small airway epithelial cell growth medium (SAGM, # CC-3118, Lonza, Basal, Switzerland) supplemented with 1 μM A83-01, 1 μM DMH1, and 10 μM Y27632, and incubated for an additional 2 h at 37°C. The AAV-6 was then gently removed to avoid disturbing the cells which were again cultured in PSY till ready for passaging.

If IDLV was used to deliver the donor DNA, BCs were exposed to a dose of 100 TU/cell (titer units/cell) in a 1.5 mL tube at 37°C, in presence of 200 μL of PSY supplemented with 8 μg/mL polybrene (#TR-1003, Millipore-Sigma, Burlington, MA, USA) with or without 0.5 μM AZD7648 (#50-217-3399, Fisher Scientific, Pittsburgh, PA, USA). BCs were then plated in PSY supplemented with 8 μg/mL polybrene with or without 0.5 μM AZD7648, and incubated an additional 4 h at 37°C. The IDLV was then removed, and cells were cultured in PSY supplemented with or without 0.5 μM AZD7648. When AZD7648 was used, medium was changed after 24 h from the initial treatment of the IDLV as previously described.[37] Cells were then maintained in PSY till ready for passaging.

After 7–10 days, the cells were harvested for flow cytometry, in case of reporter donors, or DNA isolation, in case of $CFTR_{2\text{-}27}$ donors, to assess the genetic modification.

ALI culture

After ∼1 week from editing, 100,000–150,000 cells were plated for each insert of 6.4 mm transwell (#3470, Corning) in PSY (both top and bottom chambers). At confluence, the medium was switched to PneumaCult ALI (#05001, Stemcell technologies) in both top and bottom chambers. After 24 h, the medium was removed from the top chamber and ALI conditions were established. Cells were maintained in ALI culture for 4 weeks before further analyses. Mucus was washed once a week by adding ∼200 μL of PBS in the top chamber and incubating for 5 min at room temperature. The PBS was then gently and completely aspirated without pipetting.

Flow cytometry

Flow cytometry was used to assess the percentage of BCs positive for the mScarlet integration. If AAV-6 was used, most of mScarlet expression derived from the non-integrated donor DNA disappeared after 1 week from the editing: the cells treated with AAV-6 donor

without induction of DSB at the target site had ~1% or less of mScarlet positivity. The frequency of mScarlet+ cells was assessed at this point and was sorted for establishment of ALI culture. The frequency of $mScarlet^+$ cells in the derived airway epithelium was reassessed after 4 weeks of ALI culture prior to luciferase assay. If IDLV was used, 9–10 days was required for exclusion of the non-integrated donor DNA, thus assessment of mScarlet positivity was performed at this time. Four weeks post-IDLV treatment, ALI cultures from TI-1(t) cells were analyzed via flow cytometry to determine the frequency of $mScarlet^+$ cells and to assess differentiation into basal ($NGFR^+CD66c^-$), secretory ($NGFR^-CD66c^+$), and other cell types ($NGFR^-CD66^-$) such as ciliated cells. Cells were stained on ice for 20 min using the following antibodies: FITC-CD66c (#551775, BD Pharmingen, San Jose, CA, USA) and APC-NGFR (#345108, Biolegend).

All dissociated cells, stained with antibodies where indicated, were resuspended in FACS buffer and filtered through 40 μm strainer. Propidium iodide was used for dead/live staining. $mScarlet^+$ cells from AAV-6 treatment were analyzed and sorted with BD FACSMelody Cell Sorter at the Flow Cytometry Service Center at the University of Texas Health Science Center at Houston. Gating strategy is shown in Figure S1C. IDLV experiments were analyzed with LSR Fortessa (BD Biosciences) at Flow Cytometry and Single Cell Core Facility at University of Alabama at Birmingham. Gating strategies are shown in Figures S3A and S3C.

Flow cytometry was also used to sort live single cells for scRNA-seq at 4 weeks of ALI culture. Briefly, epithelia were treated with Accutase (#07922, Stemcell technologies) for 1–2 h at 37°C. Every 20–30 min, cells were checked and gently pipetted to facilitate dissociation. Dissociated clumps were transferred to 1.5–2 mL tube and further incubated at 37°C until most of the cells were single cells. Dissociated cells were diluted with PBS to inhibit further digestion and spun at 300 g for 5 min. Single live cells isolated from 4-week-old ALI cultures, resuspended in FACS buffer and propidium iodide only, were sorted on ARIAII (BD Biosciences) at Flow Cytometry and Single Cell Core Facility at University of Alabama at Birmingham.

Luciferase assay

ALI cultures derived from the Luciferase reporter integrated in *CFTR* exon 1 or intron 1 ($mScarlet^+$) via AAV-6 were dissociated with Accutase as described above and split in two aliquots: one for flow cytometry prepared as described above, and one for luciferase assay. For luciferase assay, the cells were spun at 300 g for 5 min and resuspended in 100 μL of Pneumacult ALI. 50 μL of cell suspension was aliquoted in each well of 96 well/plate and each insert was analyzed in replicate. 50 μL of NanoGlo luciferase assay buffer+substrate (#N1110, Promega, Madison, WI) was aliquoted in each well and after 3 min the luminescence was read with a Cytation5 plate reader (Agilent Technologies, Santa Clara, CA, USA). The results are shown as average between the readings and relative to the control (ex1-BGH in Figure S1F).

gDNA isolation

Genomic DNA was isolated using GeneJet kit (#K0722, Thermo Fisher Scientific) following the manufacturer instructions.

Confirmation of on-target donor integration by PCR

We performed qualitative assessment of donor DNA integration using PCRs with primer pairs amplifying from outside of both 5′ and 3′ HAs (outside-outside) or between inside of donor and outside of an HA (inside-outside): primers were designed either upstream of the 5′ HA (5′ outside primer), downstream of the 3′ HA (3′ outside primer), or inside of the CFTR donor (schematic in Figure 2A). The primer sequences are indicated in Table S2. Outside-outside PCR was performed using GXL polymerase (#R050A, Takara) with an initial denaturation at 98°C for 30 s, followed by denaturation at 98°C for 10 s, annealing at 60°C for 15 s, extension 68°C for 5 min repeated for 35 cycles, and a final extension at 68°C for 3 min. PCR products were run on a 1% agarose gel (#50004, Lonza) at 120 V for 45 min, Generuler 1 kb plus ladder (#SM1332, Thermo Fisher Scientific) was used to identify the bands on the gel. The inside-outside PCRs were performed using GXL polymerase with an initial denaturation at 98°C for 30 s, followed by denaturation at 98°C for 10 s, annealing at 60°C for 15 s, extension 68°C for 1 min repeated for 30 cycles, and a final extension at 68°C for 3 min. PCR products were run on a 1% agarose gel at 120 V for 30 min, Generuler 100 bp plus ladder (#SM0321, Thermo Fisher Scientific) was used to identify the bands on the gel. In addition, we performed a semi-quantitative analysis of the targeting efficiency using the detection method described in Suzuki et al.[7] Briefly, a forward primer was designed on the 5′ HA and its sequence was added downstream of the $CFTR_{2\text{-}27}$-*BGH*. A reverse primer was designed downstream of the 3′ HA. An EcoRI restriction enzyme site was also added to the *CFTR* donor downstream of the added forward primer, to generate 62 bp + 337 bp bands upon digestion. The primer sequences are indicated in Table S2. This PCR was performed using GXL polymerase with an initial denaturation at 98°C for 30 s, followed by denaturation at 98°C for 10 s, annealing at 60°C for 15 s, extension at 68°C for 10 s repeated for 30 cycles, and a final extension at 68°C for 3 min. PCR products were digested with EcoRI-HF restriction enzyme in the provided CutSmart buffer (#R3101M, New England Biolabs) at 37°C for 1 h. Digested PCR products were run on a 3% agarose gel at 120 V for 45 min, Generuler 100 bp plus ladder was used to identify the bands on the gel. EcoRI digested bands were analyzed through densitometry with ImageJ (National Institute of Health, Bethesda, MD, USA) using the following formula: (cut band1+cut band2)/(uncut band+cut band1+cut band2)*100 = % of targeted alleles.

NGS-based quantification of on-target donor integration at 3′ HA

Using the primer sites described for the above semi-quantitative method which relies on size-unbiased amplification of the unmodified and correctly edited alleles (at the 3′ junction), NGS primers were designed and to generate a 399 bp amplicon from both alleles (Table S2). Alleles containing small indels that do not disrupt the primer-binding sites are also amplified but produce shorter

amplicon sizes. NGS libraries were prepared by performing 25 cycles of initial PCR, followed by a 10-cycle indexing PCR using Q5® High-Fidelity 2X Master Mix (M0492S, New England Biolabs). PCR products were pooled and sequenced on a NextSeq 2000 platform using a NextSeq 1000/2000 P1 XLEAP-SBS Reagent Kit (600 Cycles, Illumina, San Diego, CA, USA), at the Genetic Design and Engineering Center (GDEC), Rice University. Demultiplexed sequencing reads were analyzed using CRISPResso2[38] with the following parameters:

–amplicon_seq ACAAAGGCCAATATGTGTTGAACAATTATATTGTGATATACAACTGCCAAGCATTCCCGATTACCATGACTCCATTTAGTCAGTCCATGGGCAAATGCCATCAATGAGGACAGCCCAGGGTTTCCATATTCTCTCTTGGCTTTACATCCTATAGGAATTGGAGGGGCCCACCTCTGGGATAGGAGCCCTTCTGTCTTGAACAATGTTGTCTGAACACTAACAAATGTTGACTTTCTACCACCAGTCCCTCAATAGTCTTTTCTATTTATCCTTTTGCTGACCATGTTTTGTTATTACACAGTTGAGATTTTTCAGCTGGGAATCTGTGTTAATTTTGTATTAATTTTGATTAGCTTAACTCTCAGAGTTCTAAAAGTACCTCCTGTACCTGATATATGAC –expected_hdr_amplicon_seq ACAAAGGCCAATATGTGTTGAACCGCGACGAGGGGCCCACACCGAGGAAGTAGACTGTGCACGTTGGGGTGAATTCTAGTCAGTCCATGGGCAAATGCCATCAATGAGGACAGCCCAGGGTTTCCATATTCTCTCTTGGCTTTACATCCTATAGGAATTGGAGGGGCCCACCTCTGGGATAGGAGCCCTTCTGTCTTGAACAATGTTGTCTGAACACTAACAAATGTTGACTTTCTACACCAGTCCCTCAATAGTCTTTTCTATTTATCCTTTTGCTGACCATGTTTTGTTATTACACAGTTGAGATTTTTCAGCTGGGAATCTGTGTTAATTTTGTATTAATTTTGATTAGCTTAACTCTCAGAGTTCTAAAAGTACCTCCTGTACCTGATATATGAC –guide_seq ACTCCATTTAGTCAGTCCAT –default_min_aln_score 60 –amplicon_min_alignment_score 30 –flexiguide_homology 80 quantification_window_size 10 –quantification_window_center -3 –ignore_substitutions

Quantification of intact *CFTR* alleles by EvaGreen-based ddPCR

The forward primer used for NGS-based quantification of on-target TI at 3′ HA binds 62 bp upstream, and the reverse primer binds 288 bp downstream of the R1 CRISPR/Cas9 cut site. Large deletions resulting from CRISPR-induced DSBs can remove these primer-binding sites, rendering affected alleles undetectable by NGS.[20] To quantify the proportion of alleles missed by NGS, we developed an EvaGreen-based ddPCR copy number assay. This assay quantifies both the percentage of *CFTR* alleles with large modifications missed by NGS and intact *CFTR* alleles that are successfully amplified and detected by NGS.

Primers flanking the R1 gRNA target site (the same NGS primers without adaptor) and primers targeting a non-targeted reference gene (RPP30) were used (Table S2). Reactions were prepared in a final volume of 20 μL containing 15 ng genomic DNA, QX200 ddPCR EvaGreen Supermix (#186-4033, Bio-Rad, Hercules, CA, USA), 200 nM of each primer pair, and 10 U of KpnI-HF restriction enzyme (#R3142L, New England Biolabs). The PCR conditions were as follows: 40 cycles of 55°C for 30 s and 72°C for 1 min. In untreated cells, the target-to-reference copy number ratio is 1:1; reductions in this ratio indicate copy loss due to CRISPR-mediated genomic deletions eliminating the primer-binding sites.

The NGS-based quantification above was adjusted with the quantified intact *CFTR* alleles (shown in Figure S4C) for a more accurate calculation of genetic outcomes by accounting for alleles missed by NGS. The adjusted NGS-based quantification is calculated by multiplying (% intact *CFTR* alleles measured by ddPCR) by (% measured by NGS/100). With an example of W1282X/R1162X TI-1(t) replica C in Figure 2C; Figure S4B, first the % of large modifications (14.4%) and intact *CFTR* (85.6%) were assigned based on ddPCR measurement. Then, the proportion of intact *CFTR* (85.6%) was further broken down based on % measured by NGS (% TI = 61.8, % small indels = 31.4, and % unmodified = 6.5). Thus, the adjusted TI, small indels, and unmodified alleles values were calculated as 52.9% (85.6 × 61.8/100), 26.9% (85.6 × 31.4/100), and 5.6% (85.6 × 6.5/100), respectively.

Off-target analysis

COSMID,[39] a web-based tool for identifying CRISPR/Cas9 off-target sites, was utilized to predict potential off-target sites for the R1 gRNA, allowing up to three mismatches or one mismatch combined with a 1 bp indel, and identified 12 candidate off-target sites. Indel frequencies at the on-target site and the 12 COSMID-predicted off-target sites were analyzed. Mock and RNP-edited samples from F508del/F508del donor (n = 3 independent experiments per group, primers sequences in Table S2) underwent NGS library preparation and sequencing as described previously. Indel frequencies were analyzed using CRISPResso2.[38]

SMRT sequencing

Single molecule real-time (SMRT) sequencing was performed using PCR products amplified from gDNA of F508del/F508del untargeted, F508del/F508del + RNP, and F508del/F508del + RNP/IDLV samples from replica C. PCR reactions contained 100 ng of gDNA and 200 nM of primers in a final reaction volume of 100 μL using LongAmp Hot Start Taq 2× Master Mix (#M0533L, New England Biolabs). The PCR conditions included an initial denaturation at 94°C for 2 min, followed by 25 cycles of denaturation at 94°C for 30 s, annealing at 60°C for 30 s, and extension at 65°C for 8 min.

Following PCR, 500 ng of the purified PCR products from each sample were used for PacBio library preparation (Pacific Biosciences, Menlo Park, CA, USA). Libraries were prepared using the SMRTbell prep kit 3.0 (#102-141-700, Pacific Biosciences), including DNA damage repair, end repair/A-tailing, and SMRTbell adaptor ligation (Barcoded Overhang Adapter Kit- 8B, #101-685-100, Pacific Biosciences) according to the manufacturer’s instructions. Sequencing was performed on a PacBio Revio platform at the Baylor College of Medicine Human Genome Sequencing Center (BCM-HGSC). PacBio subreads were converted to HiFi reads, and CCS reads with average Q40 were utilized for downstream analyses.

HiFi reads were aligned to the *CFTR* reference sequence using minimap2[40] and analyzed using a custom MATLAB script.

Western blot

CFTR expression was determined by western blotting as described previously.[41] Briefly, cells grown in ALI condition were lysed with RIPA buffer containing 50 mM Tris/HCl (pH 8.0), 1% Nonidet P40, 0.5% deoxycholate, 0.1% SDS, 150 mM NaCl, and Complete Protease Inhibitor (#04693159001, Roche, Basal, Switzerland). Proteins were separated by SDS/PAGE (4%–12% Bis-Tris gel; # NP0336Box, Thermo Fisher Scientific) and transferred onto PVDF membranes (#162-0177, Bio-rad). CFTR was detected by immunoblotting with monoclonal antibody 596 (University of North Carolina at Chapel Hill) at 1:1,000 dilution. β-actin was detected with polyclonal antibody A2066 (Millipore-Sigma) at 1:2,000 dilution. Proteins were visualized by enhanced chemiluminescence (Pierce Fast Western Kit, SuperSignal West Pico, Mouse, # 35060, Thermo Fisher Scientific).

μOCT

Forty-eight hours prior to the μOCT imaging, 4-week-old ALI inserts were washed with PBS to remove accumulated mucus as described above to image freshly re-established mucus layer within 48 h. After removing the mucus, the medium in the bottom chamber was supplemented with either DMSO (#D2650, Sigma) or a combination of 5 μM VX445 (Elexacaftor), 3 μM VX661 (Tezacaftor), and 1 μM VX770 (Ivacaftor) (ETI, #S8851, #S7059, #S1144, Tocris Biosciences). In addition, all inserts were pretreated with 20 μM forskolin (#ICN19066910, Fisher Scientific). For each condition, four inserts were prepared, and two random fields were imaged at a rate of 40 frames per second and at 512 lines per frame as previously described.[42] PCL depths were quantified directly by z-projected images using ImageJ software (National Institute of Health).[42,43] MCT rate was measured using time elapsed and distance traveled of the mucus layer over multiple frames. Data are shown as individual data points lined on the median. The statistical analysis was performed using unpaired t test with Welch's correction and significance was indicated as follow: ns, not significant; $^{*}p < 0.05$; $^{**}p < 0.01$; $^{***}p < 0.001$; $^{****}p < 0.0001$.

Chloride ion transport measurement: Ussing chamber analysis

Ussing chamber experiments were conducted using the EasyMount Ussing Chamber System in voltage-clamp mode (Physiologic Instruments, Venice, FL, USA). Data acquisition and analysis were conducted with Physiologic Instruments' Acquire & Analyze software. Transwell inserts in the chambers were submerged in two different Ringer's solutions: an apical low-chloride solution (1.2 mM NaCl, 120 mM Na-gluconate, 25 mM $NaHCO_3$, 3.33 mM KH_2PO_4, 0.83 mM K_2HPO_4, 1.2 mM $CaCl_2$, 1.2 mM $MgCl_2$, and 10 mM glucose) and a standard basolateral solution (120 mM NaCl, 25 mM $NaHCO_3$, 3.33 mM KH_2PO_4, 0.83 mM K_2HPO_4, 1.2 mM $CaCl_2$, 1.2 mM $MgCl_2$, and 10 mM glucose).

While at a stable baseline, amiloride (100 μM, #08-901-00, Tocris Biosciences) was administered into the chambers to fully inhibit functionality of the epithelial sodium channel (ENaC). This was accompanied by the addition of forskolin (10 μM) to induce vectoral chloride ion transport. To evaluate CFTR-specific activity, CFTR inhibitor-172 (10 μM, #S7139, Selleck chemicals, Houston, TX, USA) was added to the apical surface, followed by the UTP (100 μM, #67-0150, Millipore-Sigma) apically to assess epithelial integrity. The changes in short-circuit current (ΔIsc) were quantified by Acquire and Analysis software. Additionally, ALI-cultured cells underwent a 48 h pre-treatment with DMSO or ETI to modulate CFTR expression and functionality before Ussing chamber evaluation. Data were presented as mean ± SD from independent experiments.

Immunostaining

After Ussing chamber assay, some of the inserts were treated with 4% PFA (#50-980-488, Fisher Scientific) overnight at 4°C and then washed with cold PBS. Fixed inserts were submitted to the Pathology Core at University of Alabama at Birmingham for paraffinization and sectioning. Sections were processed with a standard immunostaining protocol. Briefly, the sections were deparaffinized by warming up the slides at 62°C for 2 h, followed by 3 times of 3 min washes in Xylene (#X3P-1GAL, Fisher Scientific), 1 min wash in 100% ethanol (#BP2818500, Fisher Scientific), 2 times of 1 min washes in 90% ethanol, 1 min wash with 70% ethanol, and rinse in water. Slides were then incubated for 15 min at 95°C in a pressure cooker (instant-pot) immersed in 1X Vector Tris-based antigen retrieval solution (#H-3301-250, Vector labs, Newark, A, USA). The slides were then rinsed with water and PBS, and permeabilized with 0.3% Triton X-100 (#T8787, Millipore-Sigma) for 15 min at room temperature. Then, we blocked non-specific antigens with 2% BSA (#130-091-376, Milyenyi, Bergisch Gladbach, Germany) + 0.1% tween 20 (#P7949, Millipore-Sigma) in PBS for 1 h at room temperature. The primary antibodies (Table S3) were treated overnight at 4°C in blocking solution. The following day, the slides were washed three times with PBS to remove unbound antibodies, and secondary antibodies (Table S3) were treated at room temperature for 1 h in dark. After washing three times with PBS and 0.1% tween 20, Prolong Gold Antifade Mountant with DAPI (#P36935, Thermo Fisher Scientific) and cover slides were added on the slides which were then cured overnight at room temperature. Images were taken with BZ-X810 fluorescent microscope (Keyence, Osaka, Japan) through the BZ-800 viewer software (Keyence), and contrast was adjusted using BZ-800 analyzer software (Keyence).

iPSCs editing, differentiation, and FIS assay

W1282X/W1282X iPSCs were generated as part of a previous study.[44] The UAB IRB has reviewed and determined the study is considered not Human Subjects Research (IRB-300012957). iPSC were maintained on hES-qualified Matrigel (#354277, Corning) coated plates with mTeSR1 medium (#85850, StemCell Technologies) and routinely passaged with ReLeSR (#100-0483, StemCell Technologies). To perform TI-1 editing, iPSCs were detached with Accutase and gene-edited as described above for BCs but in presence of mTeSR1 supplemented with polybrene and AZD7648. After 5 days, iPSCs were dissociated into a single cell suspension and

replated at very low density (100 cells in one 10 cm dish) in presence of mTeSR1 supplemented by a cocktail of 50 nM Chroman1 (#HY-15392/CS-0775, Tocris Biosciences), 5 μM Emricasan (#S7775, Tocris Biosciences), 1X Polyamine Supplement (#P8483, Millipore-Sigma), and 0.7 μM *Trans*-ISRIB (#10082139, Tocris Biosciences) (CEPT) to isolate individual clones as previously described.[45] After about 1 week, colonies were picked and further expanded. To identify successful TI-1 clones, each clone was genotyped with qualitative PCR and gel electrophoresis analysis as described above. Parental W1282X/W1282X, one heterozygote TI-1, and one homozygote TI-1 clones were differentiated into airway progenitors as previously described.[23] On day 30 of the differentiation protocol, organoids were replated into 6 wells/clone of 96 well/plate with 10–20 organoids/well into 3.5 μL of Growth Factor Reduced (GFR) Matrigel (#354230, Corning) and maintained in growth medium without IBMX and cAMP (#I5879, B7880, Millipore-Sigma) for 24 h. FIS assay was performed by incubating the organoids into 10 μM forskolin and initiating time-lapse imaging every 20 min over the course of 8 h using Bio-Tek LionHearth microscope and Agilent BioTek Gen5 software (Agilent). Images were processed using the Agilent BioTek Gen5 software to calculate the area changes over time. Objects that were not observed in all images (fusion between adjacent objects, objects with not clear border due to background intensity, or plate noise) were manually omitted by the total area calculation. For each sample, at least 10 organoids were quantified. Data are reported as relative change over time from the total area at time 0 and averaged between at least four wells. Statistical analysis was performed using unpaired t test with Welch's correction for each TI-1 vs. parental clone, and significance was indicated as follow: $^{****}p < 0.0001$.

ATAC-seq

Airway epithelial cells cultured under ALI conditions were first washed twice with PBS to remove residual medium and debris, and then harvested using 0.25% trypsin/0.1% EDTA (#25053CI, Corning). For each sample, cell viability was assayed by Trypan Blue (#5250061, Thermo Fisher Scientific) and Omni-ATAC-seq performed on 7.5×10^4 cells, as described previously,[46] using the Diagenode Tn5 Transposase kit (#C01080001). Omni-ATAC-seq libraries were purified with Agencourt AMPure XP magnetic beads (#A63881, Beckman Coulter, Brea, CA, USA) with a sample to bead ratio of 1:1.2. Library size distributions were visualized by TapeStation (Agilent) and quantified using the KAPA Library Quantification Kit (#KK4824, Roche). Libraries were pooled and sequenced on a NovaSeqX platform (Illumina).

A modified human hg19 genome, hg19_CFTR$_{2\text{-}27}$+BGH, was constructed by inserting the *CFTR* transgene sequence into intron 1 of the *CFTR* gene at position chr7:117124405 using R v4.5.0[46] and the Biostrings v2.76.0[47] package. A corresponding genome index file was generated using SAMtools v1.10.[48]

ATAC-seq data were processed using the ENCODE ATAC-seq pipeline (v2.2.3)[49] using default settings. Briefly, single-end FASTQ files were adapter-trimmed and aligned to the custom genome assembly hg19_CFTR$_{2\text{-}27}$+BGH using Bowtie2. Aligned reads were filtered to remove low-quality alignments, PCR duplicates, and mitochondrial reads. Quality control metrics—including fragment length distribution, library complexity, and transcription start site (TSS) enrichment—were generated automatically by the pipeline to assess data quality. Peaks were identified using MACS2 on each sample, and reproducible peaks were determined using the irreproducible discovery rate (IDR) framework. For each sample with technical replicates, the replicate BAM files were merged using SAMtools v1.10.[48] DeepTools v3.2.07[50] was used to generate bigWigs for each BAM file.

sc-RNA-seq: Library preparation and sequencing

Single cells suspension from 4 weeks ALI cultures were prepared as described above. The libraries were generated by the Flow Cytometry and Single Cell Core Facility core at University of Alabama at Birmingham using 10x Genomics 3′ V4 kit (#PN-1000691, 10× Genomics, Pleasanton, CA, USA) and following the company's guide. Briefly, sorted single cells suspension, 10× barcoded gel beads (#PN 2001128), and partitioning oil (#PN 2001213) were loaded into 10× Genomics GEM-X 3′ Chip (#PN 2001097) to capture single cells in nanoliter-scale oil droplets by 10x ChromiumX instrument and generate gel beads-in-emulsions (GEMs). Loaded cells were captured in oil droplets, each with a gel bead containing oligos bearing a bead-unique barcode and a 3′ end poly T sequence. Single cells were lysed in droplets, the released mRNA binded to barcoded poly T, and full length of single-strand cDNA from each single cells were synthesized by "template switch" mechanism. GEMs containing single cell barcoded-cDNA were broken and all single cells cDNA libraries were pooled together. Pooled cDNA were cleaned up by DynaBeads MyOne Silane beads (#PN 2000048) and then preamplified by PCR to generate sufficient mass for sequencing library construction. The sequencing libraries were constructed as 3′-biased whole transcriptome libraries by following steps of cDNA fragmentation, end repair and A-tailing adaptor ligation, and sample index PCR amplification. The final constructed 3′-biased single cell libraries were sequenced by Illumina NextseqX plus machine (Illumina), targeting for 20,000 read pairs/cell, and the sequencing cycles consisted of 28 bp for read 1, 90 bp for read 2 and 10 bp for i7 and i5.

sc-RNA-seq: Data analysis

10× scRNA data were processed with CellRanger 9.0.1 using an hg38 reference transcriptome with an additional chromosome containing the last 351 bases of the 3′-end of the *CFTR$_{2\text{-}27}$-BGH* construct for identification of reads that mapped unambiguously to the *CFTR$_{2\text{-}27}$-BGH* derived transcript (corrected *CFTR*). The default setting, include-introns true, was used for cellranger count. Reads aligning to *CFTR* were labeled as originating from mutant transcript if they (1) overlapped the native 3′-UTR or any intronic positions beyond the insertion of the *CFTR$_{2\text{-}27}$-BGH* or (2) carried any mutant allele (F508del, G542X, R785X, in the respective samples). All remaining reads that could derive from either species of transcript were not assigned. Total *CFTR* counts were computed as the sum of corrected *CFTR*, mutant *CFTR*, and unassigned *CFTR*.

For each sample, 5,000 cells were randomly selected to facilitate processing. Data analysis was then performed with Seurat 5.0.[51,52] Cells with mitochondrial reads exceeding 15% of reads or with fewer than 200 genes were removed. Normalization was performed with SCTransform with regression on the percent of mitochondrial genes and percent of ribosomal genes. Analysis included principal component analysis, clustering with the Leiden algorithm (cluster resolution 0.5) and 2D UMAPs generated based on 15 principal components. All samples (including one non-CF) were integrated with integration anchors based on SCT normalization (20 principal components, with untreated samples as reference sample). 2D UMAPs for the integration were generated based on 20 principal components.

Cell-type classification: we used 50 signature genes for each cell type, basal, secretory, ciliated, FOXN4+ cells, ionocytes, and PNECs, previously selected using pairwise inter-cell-type differential expression on annotated primary BCs in Pneumacult ALI.[53] Cell-type signature scores were then computed for each cell following Tirosh et al.[54] For each cell type, signature scores were fitted to a Gaussian mixture model of up to 3 components using the expectation maximization (EM) algorithm and the Bayesian inference criteria. The final components for a cell type represent cells of that cell type, cells not of that cell type, and potentially transitional cells. These models were used to assign cell types to each cell in the current data, prior to integration. Assigned cell types were then mapped to the integration.

For each cell type, differential expression was performed by applying Seurat's FindMarkers (V5) to log-normalized data to compare the TI-1(t) sample to the matched, untreated control.

For violin plots, normalization was performed with the Seurat NormalizeData function with normalization.method set "LogNormalize" and scale.factor set to 10,000.

Statistics

Statistics analysis was performed with Prism software (GraphPad by Dotmatics, Boston, MA, USA). Used tests and *p* values are specified for each assay in "materials and methods" section above. All graph were prepared using Prism software, except for Figure S1 which were prepared using Excel (Microsoft, Redmond, WA, USA).

DATA AND CODE AVAILABILITY

All data needed to evaluate the conclusions in the paper are present in the paper and/or the supplemental information. PacBio and Illumina sequencing data are accessible at the Sequence Read Archive (SRA): PRJNA1381737. ATAC-seq data are accessible at Gene Expression Omnibus (GEO) repository: GSE318445. Sc-RNA-seq data are accessible at SRA: PRJNA1420171. Analysis scripts and donor DNA sequences are available upon request to the lead contact, Brian R. Davis, at bdavis34@uab.edu.

ACKNOWLEDGMENTS

This work was funded by the Cystic Fibrosis Foundation (CFF) with awards DAVIS19XX0, DAVIS22G0-COLLAB, and DAVIS24R0 to B.R.D.; HARRIS23XX0 to A.H.; BAO19XX0 and BAO19XX1 to G.B. Timeline schematics and graphic abstract were prepared with Biorender. We would like to thank Dr. Lianwu (Karl) Fu from the Gregory Fleming Cystic Fibrosis research center at UAB for performing the western blot.

AUTHOR CONTRIBUTIONS

Conceptualization, S.S. and B.R.D.; investigation, C.B., S.S., S.H.P., D.N., H.W., M.A.H., D.N., A.U., S.-H.L., Z.L., and A.R.; formal analysis, C.B., S.H.P., K.L., A.S., and B.T.; supervision, R.Z., H.C.V., E.J.S., G.M.S., M.M., J.P.C., A.H., G.B., and B.R.D.; writing – original draft, C.B., S.S., and B.R.D.; writing – revision, C.B., S.S., S.H.P., A.S., B.T., E.J.S., G.M.S., J.P.C., A.H., G.B., and B.R.D.

DECLARATION OF INTERESTS

H.C.V. and M.M. are inventors on a patent application related to the construction and use of partial *CFTR* cDNA as therapeutic approach. E.J.S. is an inventor on patent applications related to the development of compounds that can modulate CFTR protein and serves as one of board directors of the Cystic Fibrosis Foundation, a not-for-profit organization. A.R. is an inventor on patent applications related to the development of compounds that can modulate CFTR protein.

SUPPLEMENTAL INFORMATION

Supplemental information can be found online at https://doi.org/10.1016/j.omtn.2026.103123.

REFERENCES

1. Anderson, M.P., Gregory, R.J., Thompson, S., Souza, D.W., Paul, S., Mulligan, R.C., Smith, A.E., and Welsh, M.J. (1991). Demonstration That CFTR Is a Chloride Channel by Alteration of Its Anion Selectivity. Science *253*, 202–205. https://doi.org/10.1126/science.1712984.

2. Kerem, B., Rommens, J.M., Buchanan, J.A., Markiewicz, D., Cox, T.K., Chakravarti, A., Buchwald, M., and Tsui, L.C. (1989). Identification of the cystic fibrosis gene: genetic analysis. Science *245*, 1073–1080. https://doi.org/10.1126/science.2570460.

3. (2024). CFTR2. https://cftr2.org/mutations_history.

4. Plasschaert, L.W., MacDonald, K.D., and Moffit, J.S. (2024). Current landscape of cystic fibrosis gene therapy. Front. Pharmacol. *15*, 1476331. https://doi.org/10.3389/fphar.2024.1476331.

5. Krishnamurthy, S., Traore, S., Cooney, A.L., Brommel, C.M., Kulhankova, K., Sinn, P.L., Newby, G.A., Liu, D.R., and McCray, P.B. (2021). Functional correction of CFTR mutations in human airway epithelial cells using adenine base editors. Nucleic Acids Res. *49*, 10558–10572. https://doi.org/10.1093/nar/gkab788.

6. Sousa, A.A., Hemez, C., Lei, L., Traore, S., Kulhankova, K., Newby, G.A., Doman, J.L., Oye, K., Pandey, S., Karp, P.H., et al. (2025). Systematic optimization of prime editing for the efficient functional correction of CFTR F508del in human airway epithelial cells. Nat. Biomed. Eng. *9*, 7–21. https://doi.org/10.1038/s41551-024-01233-3.

7. Suzuki, S., Crane, A.M., Anirudhan, V., Barillà, C., Matthias, N., Randell, S.H., Rab, A., Sorscher, E.J., Kerschner, J.L., Yin, S., et al. (2020). Highly Efficient Gene Editing of Cystic Fibrosis Patient-Derived Airway Basal Cells Results in Functional CFTR Correction. Mol. Ther. *28*, 1684–1695. https://doi.org/10.1016/j.ymthe.2020.04.021.

8. Barillà, C., Suzuki, S., Rab, A., Sorscher, E.J., and Davis, B.R. (2022). Targeted Gene Insertion for Functional CFTR Restoration in Airway Epithelium. Front. Genome Ed. *4*, 847645. https://doi.org/10.3389/fgeed.2022.847645.

9. Gosalia, N., Neems, D., Kerschner, J.L., Kosak, S.T., and Harris, A. (2014). Architectural proteins CTCF and cohesin have distinct roles in modulating the higher order structure and expression of the CFTR locus. Nucleic Acids Res. *42*, 9612–9622. https://doi.org/10.1093/nar/gku648.

10. Smith, A.N., Barth, M.L., McDowell, T.L., Moulin, D.S., Nuthall, H.N., Hollingsworth, M.A., and Harris, A. (1996). A regulatory element in intron 1 of the cystic fibrosis transmembrane conductance regulator gene. J. Biol. Chem. *271*, 9947–9954. https://doi.org/10.1074/jbc.271.17.9947.

11. Carraro, G., Langerman, J., Sabri, S., Lorenzana, Z., Purkayastha, A., Zhang, G., Konda, B., Aros, C.J., Calvert, B.A., Szymaniak, A., et al. (2021). Transcriptional analysis of cystic fibrosis airways at single-cell resolution reveals altered epithelial cell states and composition. Nat. Med. *27*, 806–814. https://doi.org/10.1038/s41591-021-01332-7.

12. Montoro, D.T., Haber, A.L., Biton, M., Vinarsky, V., Lin, B., Birket, S.E., Yuan, F., Chen, S., Leung, H.M., Villoria, J., et al. (2018). A revised airway epithelial hierarchy

includes CFTR-expressing ionocytes. Nature *560*, 319–324. https://doi.org/10.1038/s41586-018-0393-7.

13. Okuda, K., Dang, H., Kobayashi, Y., Carraro, G., Nakano, S., Chen, G., Kato, T., Asakura, T., Gilmore, R.C., Morton, L.C., et al. (2021). Secretory Cells Dominate Airway CFTR Expression and Function in Human Airway Superficial Epithelia. Am. J. Respir. Crit. Care Med. *203*, 1275–1289. https://doi.org/10.1164/rccm.202008-3198OC.

14. Kosaka, M., Sajiki, A.F., Fujita, K., Yamada, K., Kawano, K., Hirazawa, K., Matsuno, T., Takada, M., Shimozawa, N., Inoue, K.I., et al. (2025). Evaluation of the loading capacity and patterns of packaged DNA in AAV genomes of different sizes using long-read sequencing. Mol. Ther. Methods Clin. Dev. *33*, 101474. https://doi.org/10.1016/j.omtm.2025.101474.

15. Wu, Z., Yang, H., and Colosi, P. (2010). Effect of genome size on AAV vector packaging. Mol. Ther. *18*, 80–86. https://doi.org/10.1038/mt.2009.255.

16. Cai, Y., Laustsen, A., Zhou, Y., Sun, C., Anderson, M.V., Li, S., Uldbjerg, N., Luo, Y., Jakobsen, M.R., and Mikkelsen, J.G. (2016). Targeted, homology-driven gene insertion in stem cells by ZFN-loaded 'all-in-one' lentiviral vectors. eLife *5*, e12213. https://doi.org/10.7554/eLife.12213.

17. Ferrari, S., Jacob, A., Cesana, D., Laugel, M., Beretta, S., Varesi, A., Unali, G., Conti, A., Canarutto, D., Albano, L., et al. (2022). Choice of template delivery mitigates the genotoxic risk and adverse impact of editing in human hematopoietic stem cells. Cell Stem Cell *29*, 1428–1444.e9. https://doi.org/10.1016/j.stem.2022.09.001.

18. Dong, W., and Kantor, B. (2021). Lentiviral Vectors for Delivery of Gene-Editing Systems Based on CRISPR/Cas: Current State and Perspectives. Viruses *13*, 1288. https://doi.org/10.3390/v13071288.

19. Stack, J.T., Rayner, R.E., Nouri, R., Suarez, C.J., Kim, S.H., Kanke, K.L., Vetter, T.A., Cormet-Boyaka, E., and Vaidyanathan, S. (2024). DNA-PKcs inhibition improves sequential gene insertion of the full-length CFTR cDNA in airway stem cells. Mol. Ther. Nucleic Acids *35*, 102339. https://doi.org/10.1016/j.omtn.2024.102339.

20. Park, S.H., Cao, M., Pan, Y., Davis, T.H., Saxena, L., Deshmukh, H., Fu, Y., Treangen, T., Sheehan, V.A., and Bao, G. (2022). Comprehensive analysis and accurate quantification of unintended large gene modifications induced by CRISPR-Cas9 gene editing. Sci. Adv. *8*, eabo7676. https://doi.org/10.1126/sciadv.abo7676.

21. Wen, W., Quan, Z.J., Li, S.A., Yang, Z.X., Fu, Y.W., Zhang, F., Li, G.H., Zhao, M., Yin, M.D., Xu, J., et al. (2021). Effective control of large deletions after double-strand breaks by homology-directed repair and dsODN insertion. Genome Biol. *22*, 236. https://doi.org/10.1186/s13059-021-02462-4.

22. Wang, Y., Wang, Y., Chang, T., Huang, H., and Yee, J.K. (2017). Integration-defective lentiviral vector mediates efficient gene editing through homology-directed repair in human embryonic stem cells. Nucleic Acids Res. *45*, e29. https://doi.org/10.1093/nar/gkw1057.

23. Berical, A., Lee, R.E., Lu, J., Beermann, M.L., Le Suer, J.A., Mithal, A., Thomas, D., Ranallo, N., Peasley, M., Stuffer, A., et al. (2022). A multimodal iPSC platform for cystic fibrosis drug testing. Nat. Commun. *13*, 4270. https://doi.org/10.1038/s41467-022-31854-8.

24. McHugh, D.R., Steele, M.S., Valerio, D.M., Miron, A., Mann, R.J., LePage, D.F., Conlon, R.A., Cotton, C.U., Drumm, M.L., and Hodges, C.A. (2018). A G542X cystic fibrosis mouse model for examining nonsense mutation directed therapies. PLoS One *13*, e0199573. https://doi.org/10.1371/journal.pone.0199573.

25. Sanderlin, E.J., Keenan, M.M., Mense, M., Revenko, A.S., Monia, B.P., Guo, S., and Huang, L. (2022). CFTR mRNAs with nonsense codons are degraded by the SMG6-mediated endonucleolytic decay pathway. Nat. Commun. *13*, 2344. https://doi.org/10.1038/s41467-022-29935-9.

26. Choi, T., Huang, M., Gorman, C., and Jaenisch, R. (1991). A generic intron increases gene expression in transgenic mice. Mol. Cell Biol. *11*, 3070–3074. https://doi.org/10.1128/mcb.11.6.3070-3074.1991.

27. Witte, I.P., Lampe, G.D., Eitzinger, S., Miller, S.M., Berríos, K.N., McElroy, A.N., King, R.T., Stringham, O.G., Gelsinger, D.R., Vo, P.L.H., et al. (2025). Programmable gene insertion in human cells with a laboratory-evolved CRISPR-associated transposase. Science *388*, eadt5199. https://doi.org/10.1126/science.adt5199.

28. Pille, M., Avila, J.M., Park, S.H., Le, C.Q., Xue, H., Haerynck, F., Saxena, L., Lee, C., Shpall, E.J., Bao, G., et al. (2024). Gene editing-based targeted integration for correction of Wiskott-Aldrich syndrome. Mol. Ther. Methods Clin. Dev. *32*, 101208. https://doi.org/10.1016/j.omtm.2024.101208.

29. Vaidyanathan, S., Salahudeen, A.A., Sellers, Z.M., Bravo, D.T., Choi, S.S., Batish, A., Le, W., Baik, R., de la O, S., Kaushik, M.P., et al. (2020). High-Efficiency, Selection-free Gene Repair in Airway Stem Cells from Cystic Fibrosis Patients Rescues CFTR Function in Differentiated Epithelia. Cell Stem Cell *26*, 161–171.e4. https://doi.org/10.1016/j.stem.2019.11.002.

30. Mall, M.A., Burgel, P.-R., Castellani, C., Davies, J.C., Salathe, M., and Taylor-Cousar, J.L. (2024). Cystic fibrosis. Nat. Rev. Dis. Primers *10*, 53. https://doi.org/10.1038/s41572-024-00538-6.

31. Birket, S.E., Chu, K.K., Houser, G.H., Liu, L., Fernandez, C.M., Solomon, G.M., Lin, V., Shastry, S., Mazur, M., Sloane, P.A., et al. (2016). Combination therapy with cystic fibrosis transmembrane conductance regulator modulators augment the airway functional microanatomy. Am. J. Physiol. Lung Cell. Mol. Physiol. *310*, L928–L939. https://doi.org/10.1152/ajplung.00395.2015.

32. Vijaykumar, K., Leung, H.M., Barrios, A., Wade, J., Hathorne, H.Y., Nichols, D.P., Tearney, G.J., Rowe, S.M., and Solomon, G.M. (2024). Longitudinal improvements in clinical and functional outcomes following initiation of elexacaftor/tezacaftor/ivacaftor in patients with cystic fibrosis. Heliyon *10*, e29188. https://doi.org/10.1016/j.heliyon.2024.e29188.

33. Vaidyanathan, S., Baik, R., Chen, L., Bravo, D.T., Suarez, C.J., Abazari, S.M., Salahudeen, A.A., Dudek, A.M., Teran, C.A., Davis, T.H., et al. (2022). Targeted replacement of full-length CFTR in human airway stem cells by CRISPR-Cas9 for pan-mutation correction in the endogenous locus. Mol. Ther. *30*, 223–237. https://doi.org/10.1016/j.ymthe.2021.03.023.

34. Yarnall, M.T.N., Ioannidi, E.I., Schmitt-Ulms, C., Krajeski, R.N., Lim, J., Villiger, L., Zhou, W., Jiang, K., Garushyants, S.K., Roberts, N., et al. (2023). Drag-and-drop genome insertion of large sequences without double-strand DNA cleavage using CRISPR-directed integrases. Nat. Biotechnol. *41*, 500–512. https://doi.org/10.1038/s41587-022-01527-4.

35. Pandey, S., Gao, X.D., Krasnow, N.A., McElroy, A., Tao, Y.A., Duby, J.E., Steinbeck, B.J., McCreary, J., Pierce, S.E., Tolar, J., et al. (2025). Efficient site-specific integration of large genes in mammalian cells via continuously evolved recombinases and prime editing. Nat. Biomed. Eng. *9*, 22–39. https://doi.org/10.1038/s41551-024-01227-1.

36. (2024). ClinVar. https://www.ncbi.nlm.nih.gov/clinvar/.

37. Stack, J.T., Rayner, R.E., Nouri, R., Suarez, C.J., Kim, S.H., Kanke, K.L., Vetter, T.A., Cormet-Boyaka, E., and Vaidyanathan, S. (2024). DNA-PKcs Inhibition Improves Sequential Gene Insertion of the Full-Length CFTR cDNA in Airway Stem Cells. Mol Ther Nucleic Acids *35*, 102339. https://doi.org/10.1016/j.omtn.2024.102339.

38. Clement, K., Rees, H., Canver, M.C., Gehrke, J.M., Farouni, R., Hsu, J.Y., Cole, M.A., Liu, D.R., Joung, J.K., Bauer, D.E., and Pinello, L. (2019). CRISPResso2 provides accurate and rapid genome editing sequence analysis. Nat. Biotechnol. *37*, 224–226. https://doi.org/10.1038/s41587-019-0032-3.

39. Cradick, T.J., Qiu, P., Lee, C.M., Fine, E.J., and Bao, G. (2014). COSMID: A Web-based Tool for Identifying and Validating CRISPR/Cas Off-target Sites. Mol. Ther. Nucleic Acids *3*, e214. https://doi.org/10.1038/mtna.2014.64.

40. Li, H. (2018). Minimap2: pairwise alignment for nucleotide sequences. Bioinformatics *34*, 3094–3100. https://doi.org/10.1093/bioinformatics/bty191.

41. Chen, J., Thrasher, K., Fu, L., Wang, W., Aghamohammadzadeh, S., Wen, H., Tang, L., Keeling, K.M., Falk Libby, E., Bedwell, D.M., and Rowe, S.M. (2023). The synthetic aminoglycoside ELX-02 induces readthrough of G550X-CFTR producing superfunctional protein that can be further enhanced by CFTR modulators. Am. J. Physiol. Lung Cell. Mol. Physiol. *324*, L756–L770. https://doi.org/10.1152/ajplung.00038.2023.

42. Liu, L., Chu, K.K., Houser, G.H., Diephuis, B.J., Li, Y., Wilsterman, E.J., Shastry, S., Dierksen, G., Birket, S.E., Mazur, M., et al. (2013). Method for quantitative study of airway functional microanatomy using micro-optical coherence tomography. PLoS One *8*, e54473. https://doi.org/10.1371/journal.pone.0054473.

43. Birket, S.E., Chu, K.K., Liu, L., Houser, G.H., Diephuis, B.J., Wilsterman, E.J., Dierksen, G., Mazur, M., Shastry, S., Li, Y., et al. (2014). A functional anatomic defect of the cystic fibrosis airway. Am. J. Respir. Crit. Care Med. *190*, 421–432. https://doi.org/10.1164/rccm.201404-0670OC.

44. Li, C., Liu, Z., Anderson, J., Liu, Z., Tang, L., Li, Y., Peng, N., Chen, J., Liu, X., Fu, L., et al. (2023). Prime editing-mediated correction of the CFTR W1282X mutation in iPSCs and derived airway epithelial cells. PLoS One *18*, e0295009. https://doi.org/10.1371/journal.pone.0295009.

45. Tristan, C.A., Hong, H., Jethmalani, Y., Chen, Y., Weber, C., Chu, P.-H., Ryu, S., Jovanovic, V.M., Hur, I., Voss, T.C., et al. (2023). Efficient and safe single-cell cloning of human pluripotent stem cells using the CEPT cocktail. Nat. Protoc. *18*, 58–80. https://doi.org/10.1038/s41596-022-00753-z.

46. R Foundation for Statistical Computing, R Core Team (2025). R: A Language and Environment for Statistical Computing. https://www.R-project.org/.

47. Pagès, H.A.P., Gentleman, R., and DebRoy, S. (2025). Biostrings: Efficient manipulation of biological strings. https://bioconductor.org/packages/Biostrings.

48. Danecek, P., Bonfield, J.K., Liddle, J., Marshall, J., Ohan, V., Pollard, M.O., Whitwham, A., Keane, T., McCarthy, S.A., Davies, R.M., and Li, H. (2021). Twelve years of SAMtools and BCFtools. GigaScience *10*, giab008. https://doi.org/10.1093/gigascience/giab008.

49. Hitz, B.C., Jin-Wook, L., Jolanki, O., Kagda, M.S., Graham, K., Sud, P., Gabdank, I., Strattan, J.S., Sloan, C.A., Dreszer, T., et al. (2023). The ENCODE Uniform Analysis Pipelines. Preprint at bioRxiv. https://doi.org/10.1101/2023.04.04.535623.

50. Ramírez, F., Ryan, D.P., Grüning, B., Bhardwaj, V., Kilpert, F., Richter, A.S., Heyne, S., Dündar, F., and Manke, T. (2016). deepTools2: a next generation web server for deep-sequencing data analysis. Nucleic Acids Res. *44*, W160–W165. https://doi.org/10.1093/nar/gkw257.

51. Hao, Y., Hao, S., Andersen-Nissen, E., Mauck, W.M., III, Zheng, S., Butler, A., Lee, M.J., Wilk, A.J., Darby, C., Zager, M., et al. (2021). Integrated analysis of multimodal single-cell data. Cell *184*, 3573–3587.e29. https://doi.org/10.1016/j.cell.2021.04.048.

52. Hao, Y., Stuart, T., Kowalski, M.H., Choudhary, S., Hoffman, P., Hartman, A., Srivastava, A., Molla, G., Madad, S., Fernandez-Granda, C., and Satija, R. (2024). Dictionary learning for integrative, multimodal and scalable single-cell analysis. Nat. Biotechnol. *42*, 293–304. https://doi.org/10.1038/s41587-023-01767-y.

53. Suzuki, S., Acosta Sandoval, N.V., Doddipalli, S., Hassan, D.M., Barillà, C., Rab, A., Huang, M.F., Avila, J.M., Tabak, B., Lu, J., et al. (2025). Generation of Region-specific Airway Basal Stem Cells from Human Pluripotent Stem Cells via Regulation of BMP-NOGGIN Axis. Preprint at bioRxiv. https://doi.org/10.1101/2025.10.01.657190.

54. Tirosh, I., Izar, B., Prakadan, S.M., Wadsworth, M.H., Treacy, D., Trombetta, J.J., Rotem, A., Rodman, C., Lian, C., Murphy, G., et al. (2016). Dissecting the multicellular ecosystem of metastatic melanoma by single-cell RNA-seq. Science *352*, 189–196. https://doi.org/10.1126/science.aad0501.

OMTN, Volume *37*

Supplemental information

Targeted insertion of partial *CFTR* cDNA replicates native *CFTR* expression with restored channel function in CF airway epithelium

Cristina Barillà, Shingo Suzuki, So Hyun Park, Divyasri Nalluri, Hui Wen, Mohamed A. Hanafy, Daniel Betancourth, Ayushi Umrigar, Shih-Hsing Leir, Katreya Lovrenert, Andrey Sivachenko, Barbara Tabak, Zhong Liu, Rui Zhao, Hillary C. Valley, Andras Rab, Eric J. Sorscher, George M. Solomon, Martin Mense, John P. Carulli, Ann Harris, Gang Bao, and Brian R. Davis

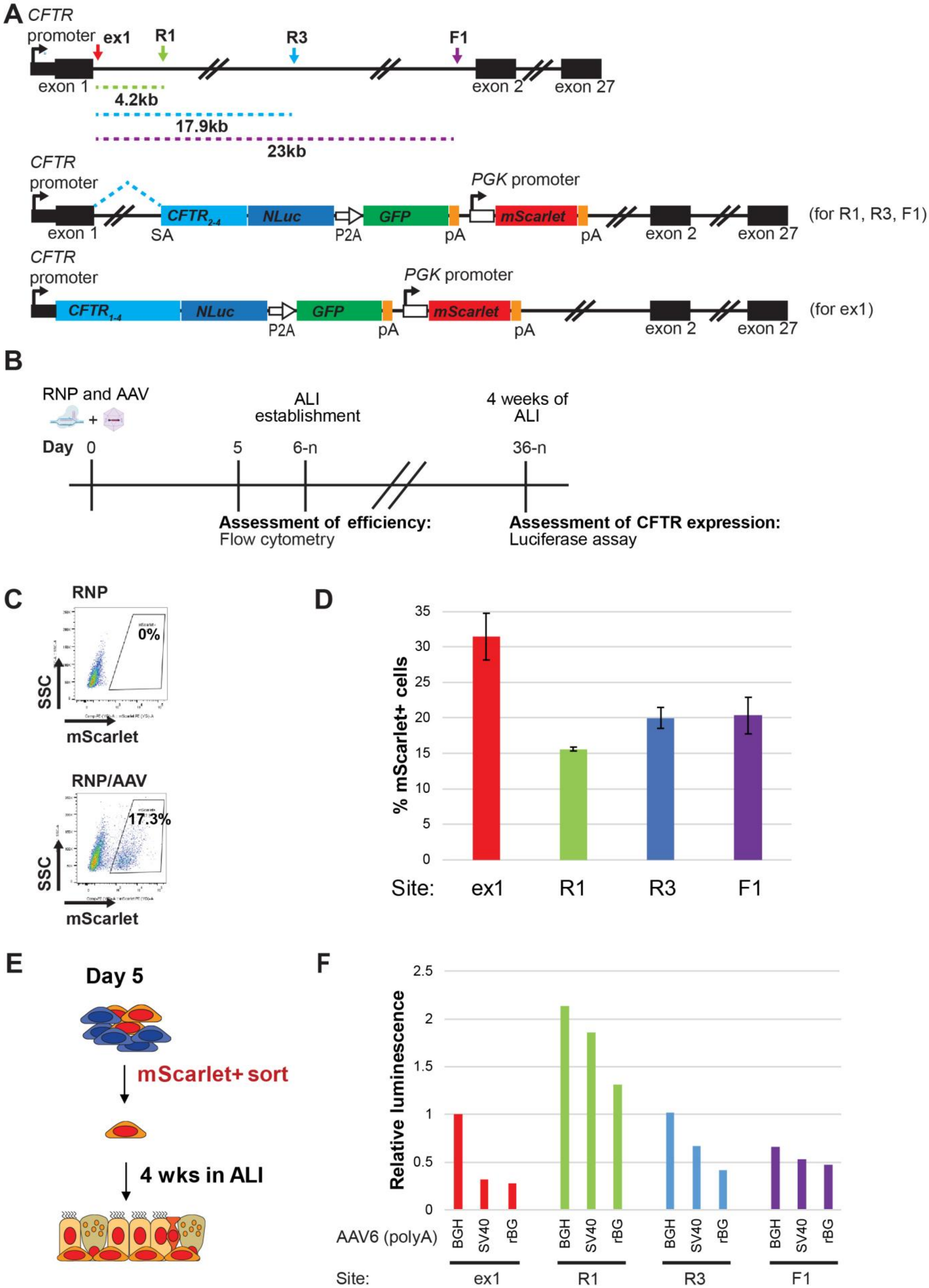
A
CFTR promoter
ex1
R1
R3
F1
exon 1
exon 2
exon 27
4.2kb
17.9kb
23kb
CFTR promoter
PGK promoter
CFTR2-4
NLuc
GFP
mScarlet
SA
P2A
pA
(for R1, R3, F1)
CFTR1-4
(for ex1)
B
RNP and AAV
ALI establishment
4 weeks of ALI
Day 0
5
6-n
36-n
Assessment of efficiency:
Flow cytometry
Assessment of CFTR expression:
Luciferase assay
C
RNP
0%
SSC
mScarlet
RNP/AAV
17.3%
D
% mScarlet+ cells
Site: ex1 R1 R3 F1
E
Day 5
mScarlet+ sort
4 wks in ALI
F
Relative luminescence
AAV6 (polyA)
BGH SV40 rBG
Site: ex1 R1 R3 F1

Figure S1: **Evaluation of *CFTR* intron 1 insertion sites and polyA sequences**. A) Schematics of *CFTR* with focused view on intron 1. The top schematic shows the native structure of intron 1 with vertical arrows marking the position of different gRNAs tested. The middle schematic shows the DNA structure after insertion of the reporter at one of the intron 1 sites. The blue dashed line represents the splicing event required to achieve expression of the *CFTR-NanoLuc* chimera. The bottom schematic shows the DNA structure after insertion of the same reporter but inserted at exon 1. B) Experiment workflow and timeline to assess different reporter transgenes. C) Flow cytometric gating to identify mScarlet+ targeted cells after selection of single and live cells. D) Frequency of mScarlet+ cells at each insertion site measured seven days after transduction. Mean±SD for each insertion site (ex1, R1, R3, F1) reflects the pooled results of three donors -- differing in their polyA signal -- targeted via homology arms to each insertion site. E) Strategy to generate ALI cultures derived from enriched edited cells. F) Summary of luciferase assay, performed at 4 weeks of ALI culture, showing the activity at each insertion site (ex1, R1, R3, F1) for donors DNA differing in their polyA signal culture. The data are reported relative to ex1 site with BGH.

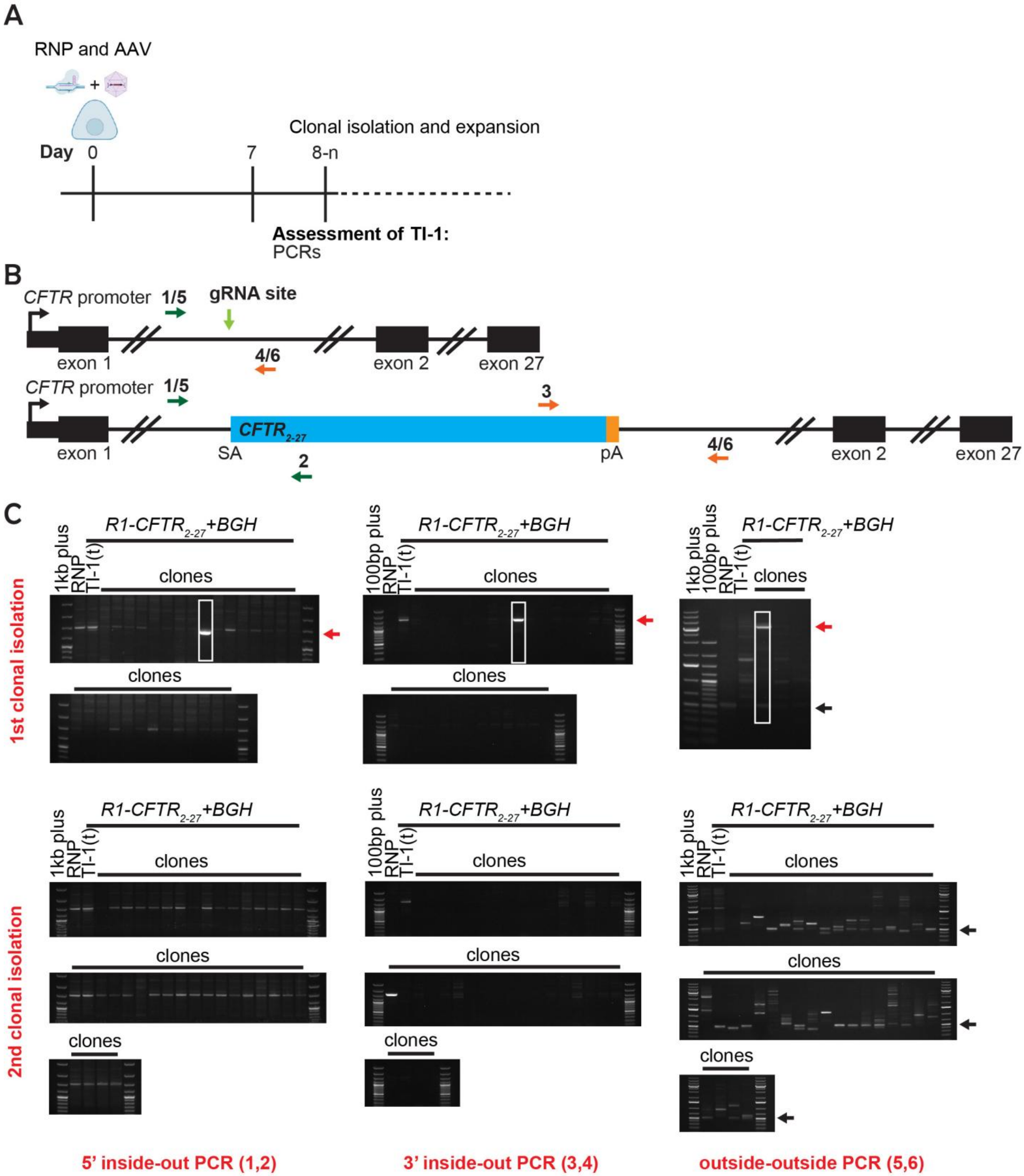

Figure S2: **Inefficient targeted insertion of AAV-6 delivered *CFTR*$_{2\text{-}27}$.** A) Experiment workflow and timeline for AAV-6 delivery for assessing insertion efficiency through AAV-6 delivery of the *CFTR*$_{2\text{-}27}$ transgene. B) Schematic of *CFTR* exon 1, intron 1, and exon 2 highlighting the PCR primers used for the molecular characterization (see Fig 2A). The green arrows represent primers used for the 5' inside-outside PCR; orange arrows represent primers used for the 3' inside-outside PCR; the

green arrow most left and the orange arrow at the most right represent primers used outside-outside PCR. C) Agarose gels showing PCR amplification of either prior or after clonal isolation (from two separate rounds of clonal isolation) of RNP/AAV treated basal cells. A red arrow marks amplicon band consistent with TI-1; a black arrow signifies amplified band from unmodified allele or with small indels. The white rectangle highlights the only positive sample (heterozygous for TI-1) from the three PCR analyses.

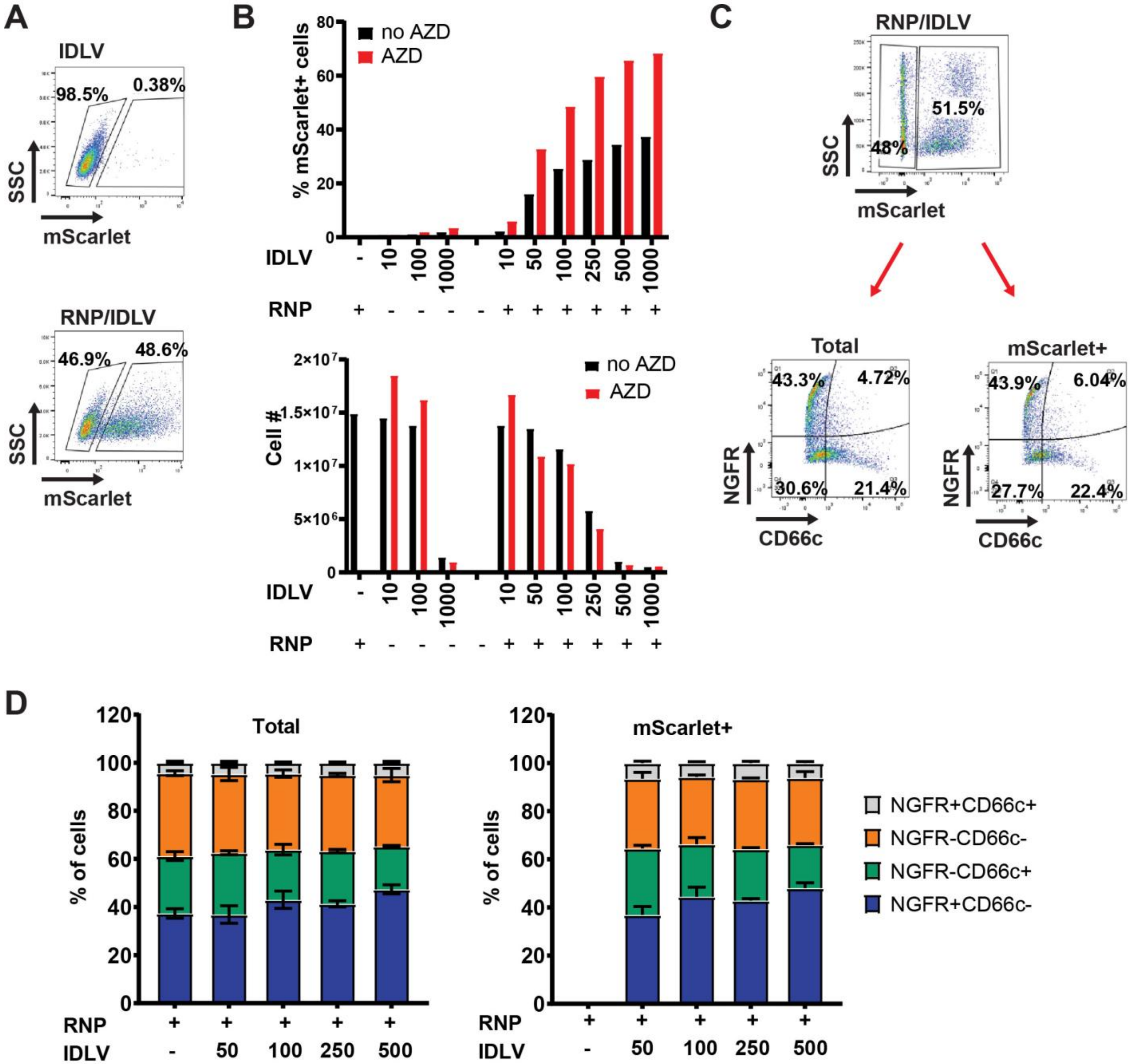

Figure S3: **IDLV delivery facilitates efficient insertion of the reporter construct, in the presence or absence of AZD7648**. A) Flow cytometry gates for identification of mScarlet+ cells after selection of single and live cells. B) Quantification of mScarlet+ cells (%) (top) and cell recovery (#) (bottom) with increasing doses of IDLV nine days post transduction. C) Flow cytometric analysis to examine expression of basal (NGFR+/CD66c-), secretory (NGFR-CD66c+), and other cells (for example, ciliated) (NGFR-CD66c-) markers in either total cells or in the mScarlet+ population. Pre-gating was performed to only show single live cells. D) Quantification of the cell types from C.

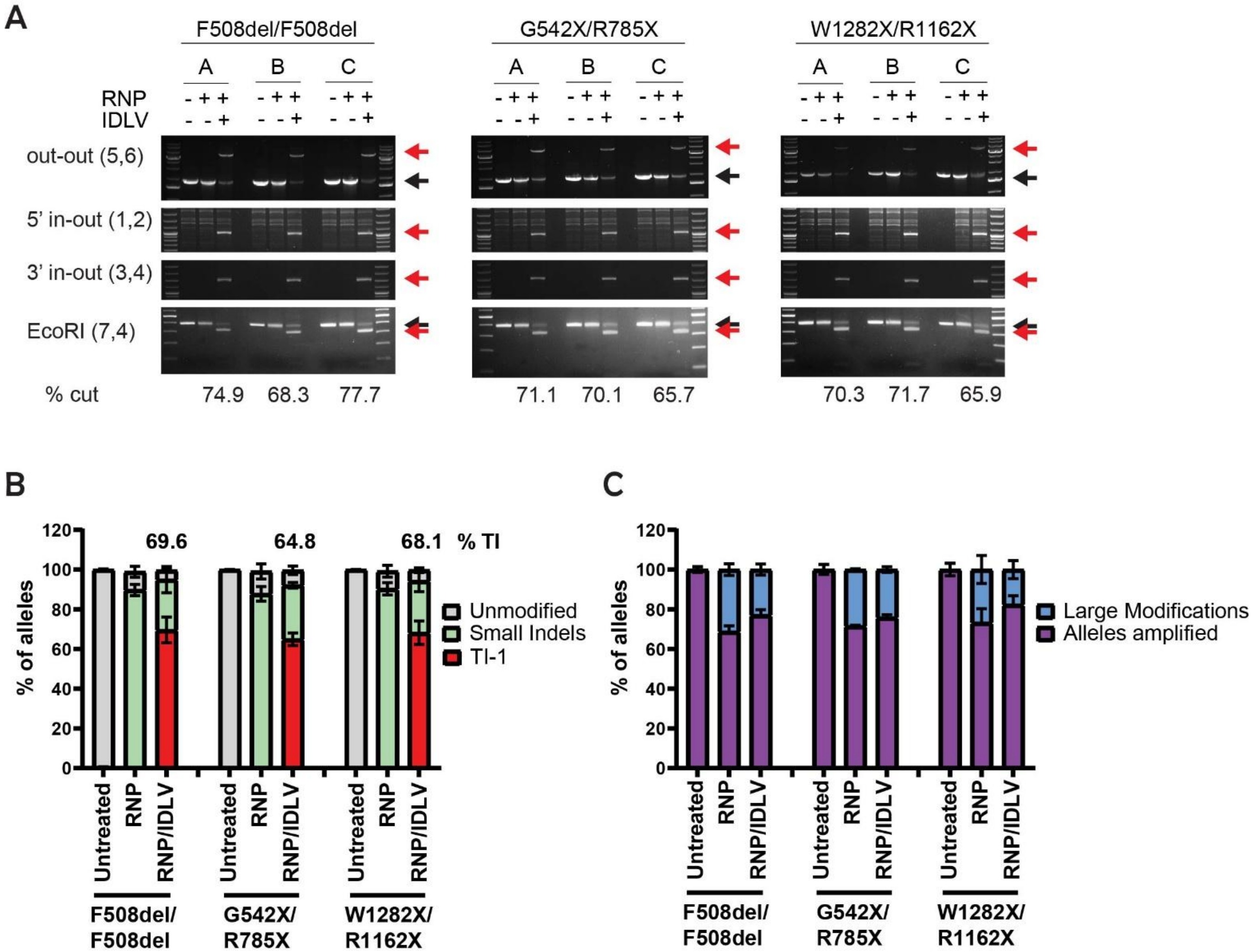

Figure S4: **Evaluation of editing efficiency with IDLV-delivered *CFTR$_{2\text{-}27}$* transgene**. A) Agarose gels from the three cell donors, showing detection of *CFTR$_{2\text{-}27}$* insertion in three independent editing experiments (labeled A, B, and C) through PCR using the primers in Fig 2A. Red arrows indicate bands consistent with successful insertion; black arrows indicate bands from unmodified or small indel alleles. B-C) Summary of deep sequencing results (B) and ddPCR copy number assay (C). ddPCR was used to quantify the fraction of PCR-amplifiable alleles measured by NGS. This value was then used to calculate the true proportions of unmodified alleles, small indels, and TI-1 events, as well as the fraction of large genomic modifications that are not amplifiable by PCR, as shown in Fig 2C. Mean±SD of three independent experiments.

A

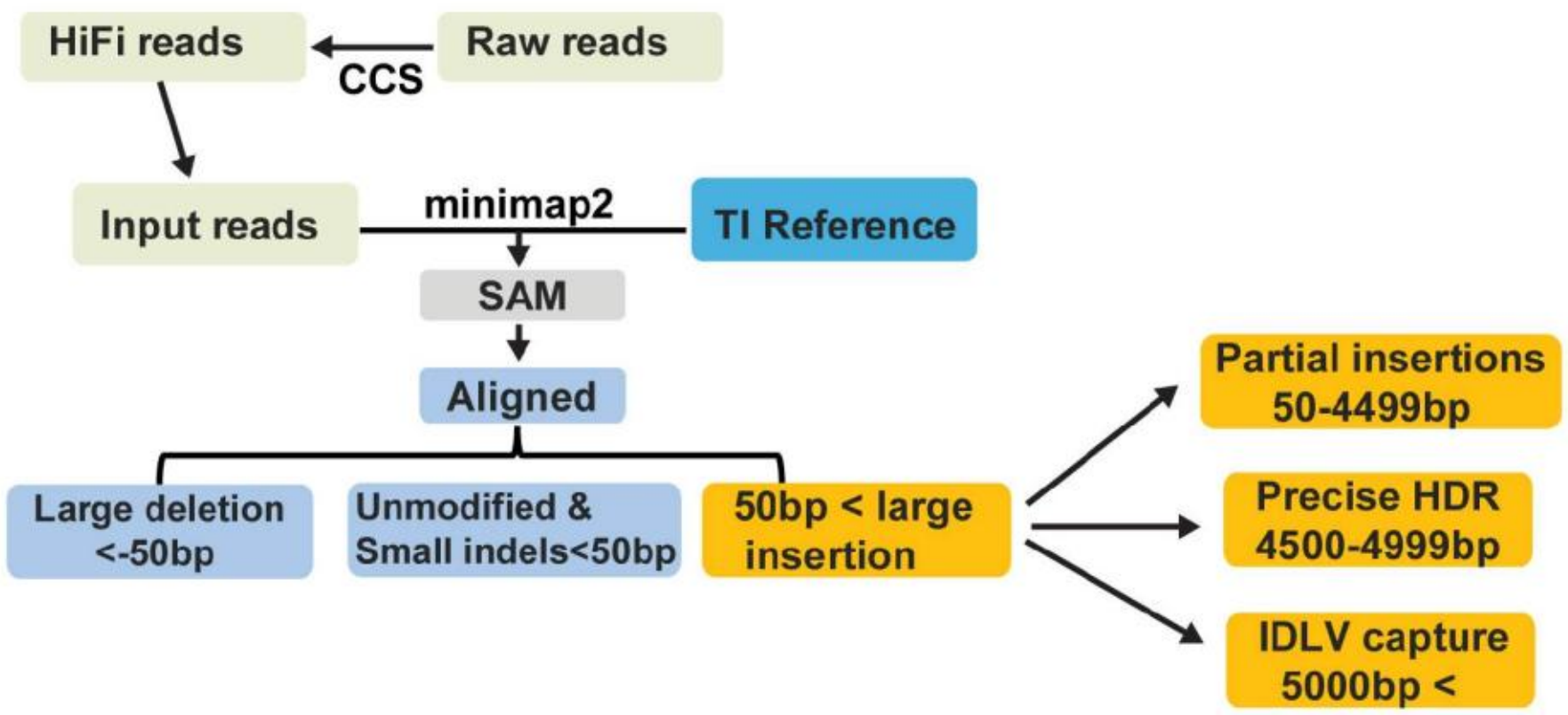

C

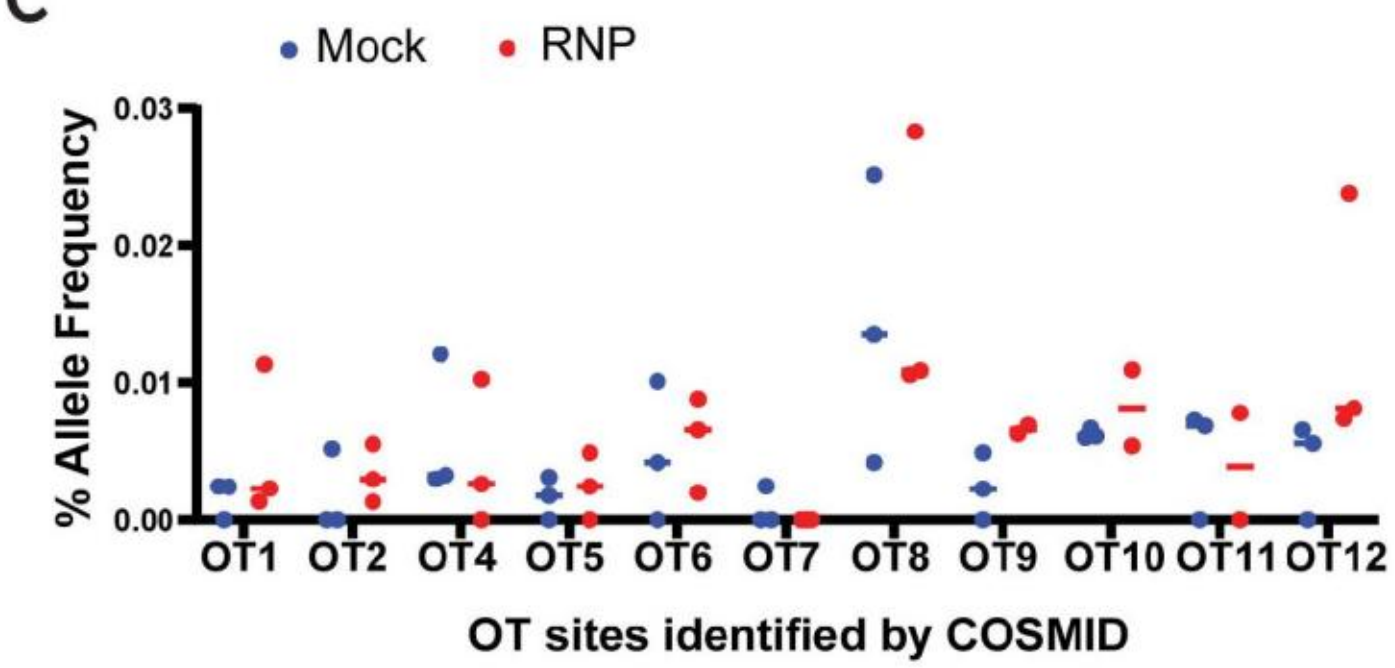

B

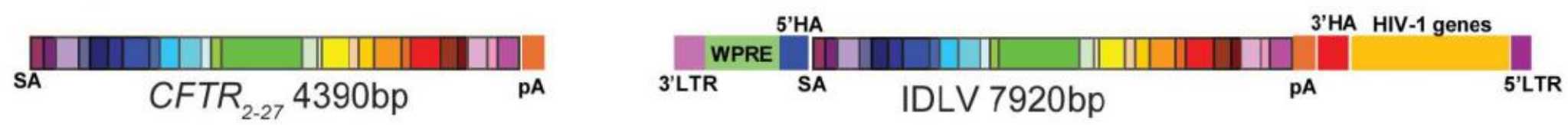

Bin size (bp)	% reads in bin
50-499	3.00
500-999	2.06
1000-1499	2.69
2000-2999	2.69
3000-3999	1.50
4000-4999	64.78
5000-5999	1.28
6000-6999	0.99
7000-7999	0.97
8000-8999	0.29
9000-9999	0.25
10000-10999	0.23
11000-11999	0.10
12000-12999	2.49
13000-13999	0.04
14000-14999	0.01
15000-15999	0.02
20000-20999	0.02

Most common insertion size (bp)	5'	3'	Representative insertion structure
382			
991			$CFTR_{23-27}$
1258			$CFTR_{22-27}$
2321			
3465			
4720 (TI-1)	HDR	HDR	
5889		HDR	
6808	HDR		
7977			
8094	HDR		
9102		HDR	
10061	HDR		
11012	HDR		
12459	HDR		
13626			
14535	HDR		
15015		HDR	
20198	HDR		

Figure S5: **Analysis of insertion events and undesired outcomes**. A) Variant calling pipeline for SMRT-seq analysis used to identify the modification events in Fig 2D. SMRT-seq generated HiFi reads via Circular Consensus Sequencing (CCS), which were aligned to the TI-1 reference sequence using minimap2. Reads were classified based on insertion and deletion sizes: events ranging from 50 bp deletions to 49 bp insertions were classified as unmodified or indels; deletions > 50 bp were classified as large deletions; and insertions ≥ 50 bp were classified as large insertions. A 4,720 bp insertion corresponds to the TI-1 allele. Large insertions between 50 and 4,499 bp reflect partial IDLV insertions. Reads classified as large insertions were further extracted and analyzed using alignment software to identify diverse IDLV capture patterns, including partial, full-length, and concatemeric IDLV capture. B) Representative insertion structures identified by SMRT-seq. The top schematics show color-coded of *CFTR*$_{2\text{-}27}$ transgene (left; each color represents a different exon) and IDLV donor (right). SMRT-seq reads were grouped by insertion size with a resolution of 1,000 bp bin, and representative structures are shown. The table indicates the size of the most common insertion within a given bin, whether the 5′ and/or 3′ junction retained an HDR structure, and a schematic representation of the most common insertion. Because IDLV capture events were highly heterogeneous, the structures shown represent the most common read within each insertion-size bin rather than all observed configurations. Many IDLV capture events retained a 5′ HDR structure, which, in principle, would allow the allele to express the full-length *CFTR* transgene from the endogenous promoter, even though the correctly targeted cassette is followed by additional IDLV-derived vector sequences. In addition, many IDLV capture events retained the 3′ HDR structure but not the 5′ HDR structure. These alleles would have been classified as TI-1 alleles by the NGS assay because they retain the primer-binding sites and generate the same expected amplicon size as a true TI allele. However, although we detected multiple alleles in this category, their combined frequency was very low. Therefore, these rare outcomes are unlikely to have substantially affected the overall NGS- or ddPCR-based quantification of targeted integration. C) Off-target analysis was performed by deep sequencing to quantify indel frequencies across the top 12 sites identified by COSMID.

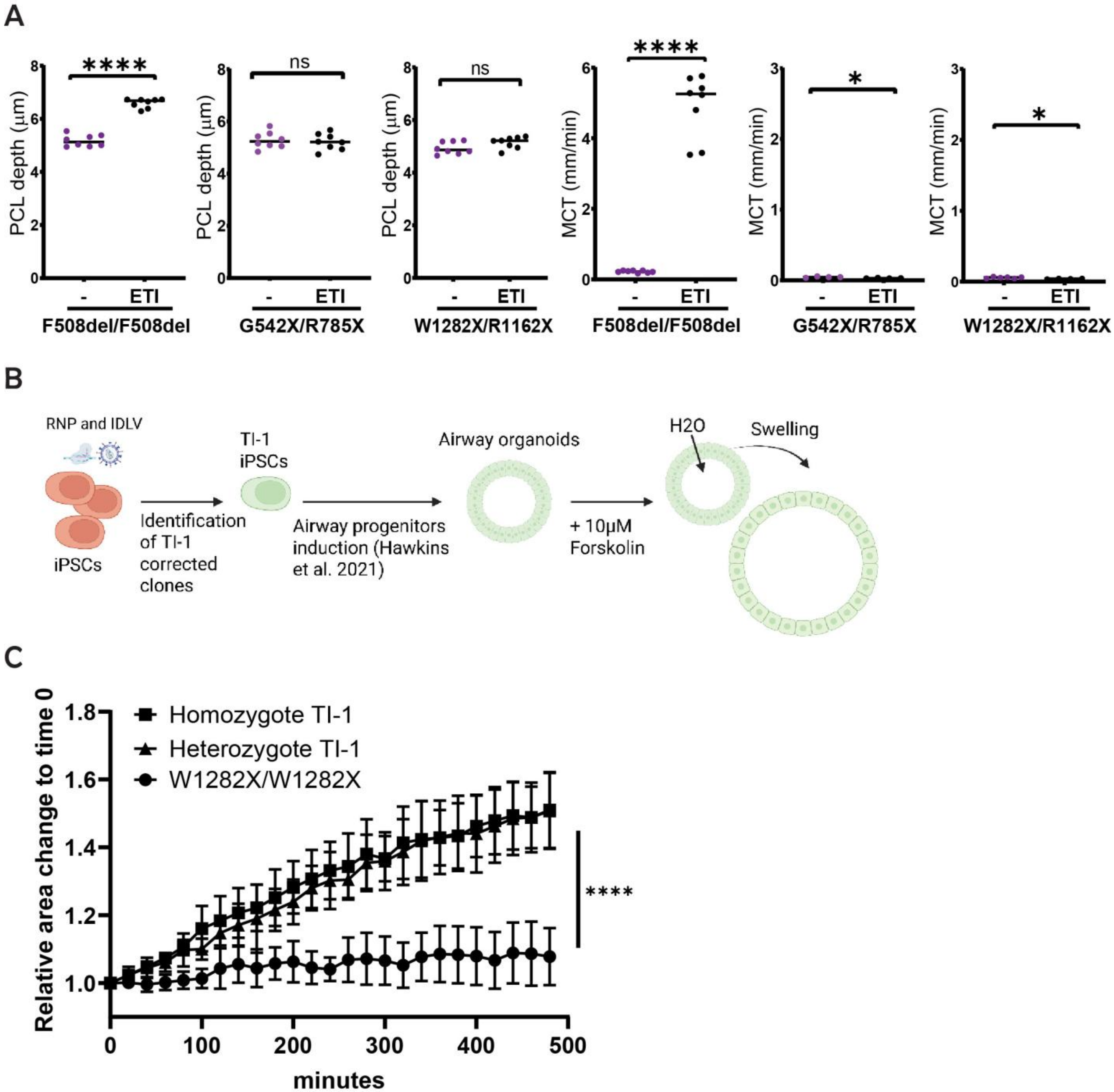

Figure S6: **CFTR functional assays**. A) μOCT summary data for PCL and MCT comparing untreated vs ETI (corresponding to Fig 3D). Data are presented as individual data points from 4 technical replicates (1-2 images each) distributed around the median. B) Schematic for TI-1 and functional evaluation in iPSCs. C) Forskolin induced swelling assay in clonal populations of edited cells vs parental, unedited cells after 30 days of differentiation of iPSCs to airway progenitors. The data are shown relative to time 0. Mean±SD from 5-6 wells.

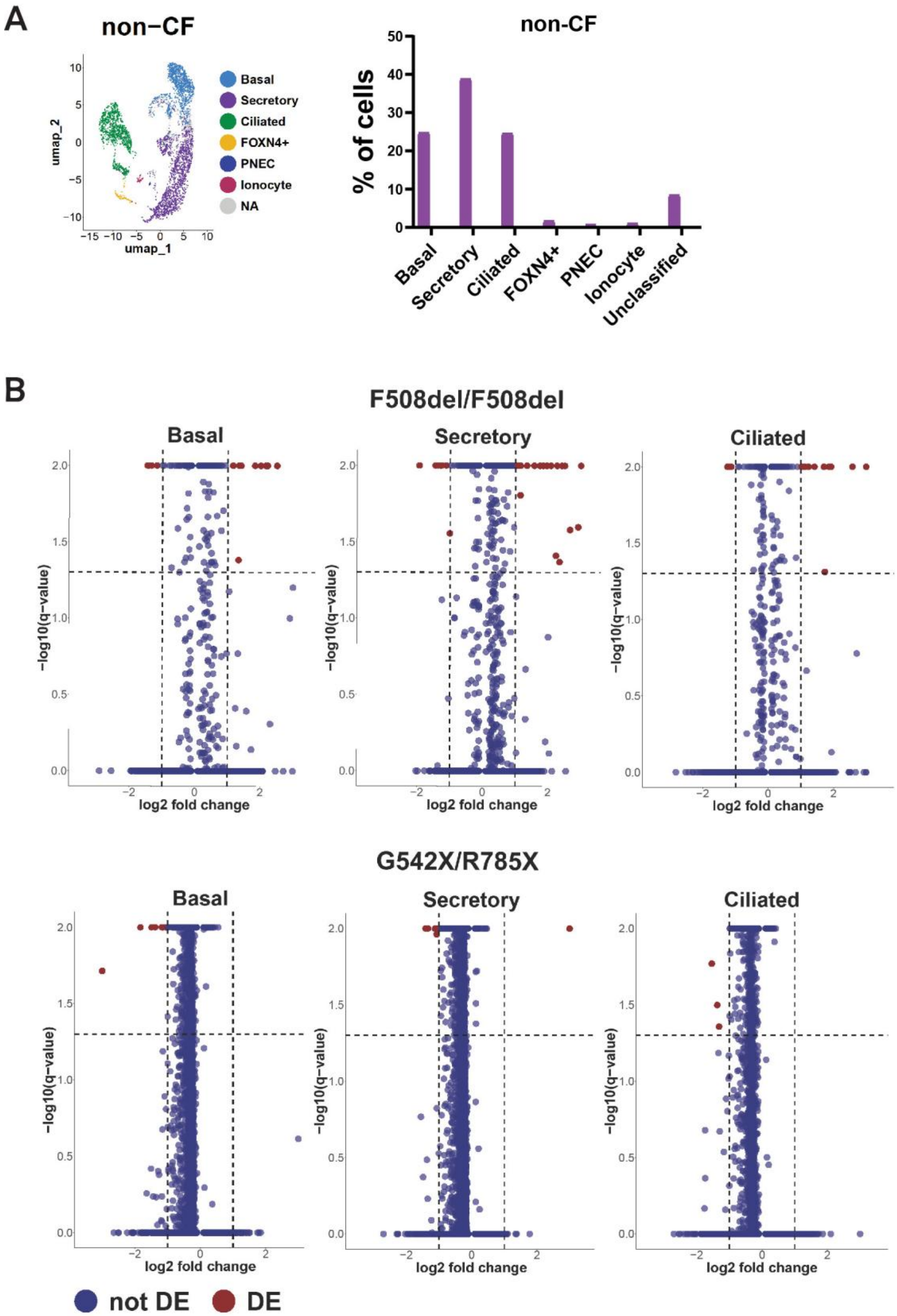
A
non−CF
Basal
Secretory
Ciliated
FOXN4+
PNEC
Ionocyte
NA
umap_1
umap_2
non-CF
% of cells
Basal
Secretory
Ciliated
FOXN4+
PNEC
Ionocyte
Unclassified
B
F508del/F508del
Basal
Secretory
Ciliated
−log10(q−value)
log2 fold change
G542X/R785X
Basal
Secretory
Ciliated
not DE
DE

Figure S7: **TI-1 treatment in basal cells minimally affects transcriptional profile of derived airway epithelium.** A) Identification and quantification of cell types in non-CF ALI cultures analyzed in parallel to samples in Fig 4B-C. UMAPs (left) and quantification (right) of each cell cluster in non-CF. B) Comparison of gene expression pattern for major cell types in untreated vs bulk edited CF ALI cultures. Volcano plot showing total gene expression in major cell types of ALI cultures comparing untreated and bulk edited primary cells. Genes that are not differentially expressed are in blue, differentially expressed are in red.

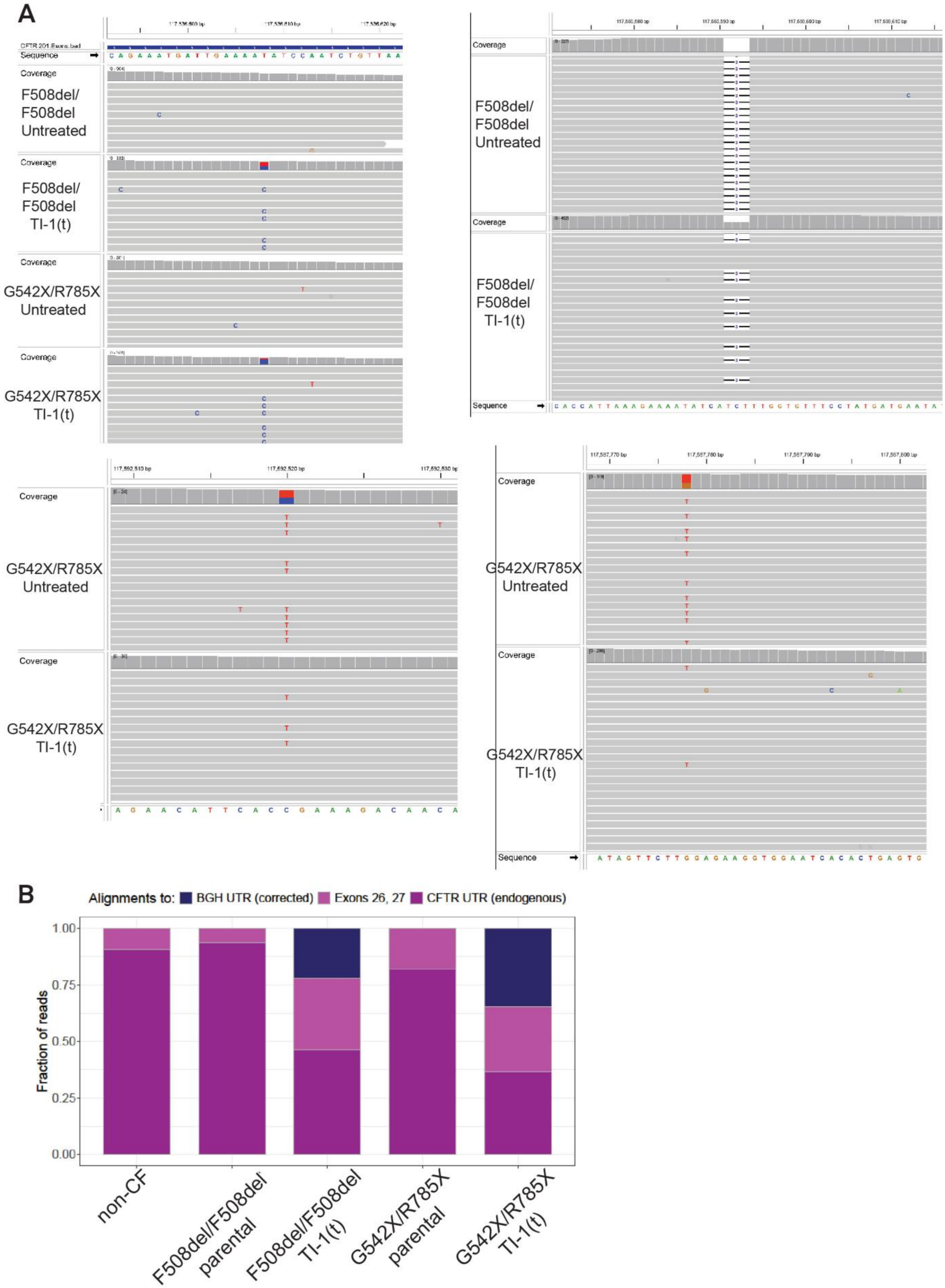
A
CFTR.201.Exons.bed
Sequence
Coverage
F508del/ F508del Untreated
F508del/ F508del TI-1(t)
G542X/R785X Untreated
G542X/R785X TI-1(t)
B
Alignments to: BGH UTR (corrected) Exons 26, 27 CFTR UTR (endogenous)
Fraction of reads
1.00
0.75
0.50
0.25
0.00
non-CF
F508del/F508del parental
F508del/F508del TI-1(t)
G542X/R785X parental
G542X/R785X TI-1(t)

Figure S8: A**lignment of sequence reads in regions that distinguish between mutant and corrected *CFTR* mRNAs**. A) Comparison of reads in unedited vs TI-1(t) cells: IGV view of the SNP (T>C) in exon 7 (top left), CTT deletion causing F508del (top right), C>T variant responsible for G542X (bottom left), and G>T variant causing R785X (bottom right). The number and percentage of reads for each are summarized in Fig 7A. B) Fraction of the reads aligning within the native 3'UTR, BGH UTR, and *CFTR* exons 26-27 used for Fig 7B.

Table S1: gRNA sequences used in this study with spCas9.

**gRNA name**	**sequences 5' -> 3'**	**PAM**
CFTRgRNA-ex1	GAAGGTGGCCAACCGAGCTT	CGG
CFTRgRNA-R1	ACTCCATTTAGTCAGTCCAT	GGG
CFTRgRNA-R3	TCCAGGATTTACAACCATAC	AGG
CFTRgRNA-F1	AAAGTTATGATCCTACCCTC	AGG

Table S2: Sequence of primers used in this study.

Table S3: List of antibodies used for immunostaining.

**Antibody**	**Cat#**	**Company**
Mouse monoclonal anti-Acetylated Tubulin (ACT) (6-11B-1)	T7451	Millipore-Sigma
Uteroglobin/SCGB1A1 Antibody (394324)	MAB4218-SP	Novus Biologicals
Rabbit monoclonal anti-MUC5AC (E309I)	61193:	Cell Signaling Technologies
Mouse monoclonal anti-MUC5B (A-3)	Sc-393952	Santa Cruz
Rabbit monoclonal anti-p75NTR/NGFR (D4B3)	8238	Cell Signaling Technologies

Mouse monoclonal anti-TP63 (4A4)	CM163A	Biocare
Rabbit monoclonal anti-KRT5 (D4U8Q)	25807	Cell Signaling Technologies
Donkey anti-mouse IgG Alexa Fluor Plus 647 Highly cross-absorbed	A32787	Thermo Fisher Scientific
Donkey anti-rabbit IgG Alexa Fluor Plus 488 Highly cross-absorbed	A32790	Thermo Fisher Scientific
Donkey anti-rat IgG Alexa Fluor Plus 555 Highly cross-absorbed	A48270	Thermo Fisher Scientific

Video S1. **F508del/F508del TI-1(t) mucus movement**. Video generated by live imaging of a representative ALI culture at 4 weeks of differentiation. Images were captured at 40 frames/second and 512 lines/frame using uOCT and used for quantifications showed in Fig. 3D.

Video S2. **W1282X/R1162X TI-1(t) mucus movement**. Video generated by live imaging of a representative ALI culture at 4 weeks of differentiation. Images were captured at 40 frames/second and 512 lines/frame using uOCT and used for quantifications showed in Fig. 3D.
